# Investigating molecular crowding within nuclear pores using polarization-PALM

**DOI:** 10.7554/eLife.28716

**Published:** 2017-09-26

**Authors:** Guo Fu, Li-Chun Tu, Anton Zilman, Siegfried M Musser

**Affiliations:** 1 Department of Molecular and Cellular Medicine, College of MedicineThe Texas A&M University Health Science CenterCollege StationUnited States; 2 Department of PhysicsUniversity of TorontoTorontoCanada; 3 Institute for Biomaterials and Biomedical EngineeringUniversity of TorontoTorontoCanada; ETH ZurichSwitzerland

**Keywords:** nuclear pores, super-resolution microscopy, PALM, polarization PALM, rotational diffusion, Human

## Abstract

The key component of the nuclear pore complex (NPC) controlling permeability, selectivity, and the speed of nucleocytoplasmic transport is an assembly of natively unfolded polypeptides, which contain phenylalanine-glycine (FG) binding sites for nuclear transport receptors. The architecture and dynamics of the FG-network have been refractory to characterization due to the paucity of experimental methods able to probe the mobility and density of the FG-polypeptides and embedded macromolecules within intact NPCs. Combining fluorescence polarization, super-resolution microscopy, and mathematical analyses, we examined the rotational mobility of fluorescent probes at various locations within the FG-network under different conditions. We demonstrate that polarization PALM (p-PALM) provides a rich source of information about low rotational mobilities that are inaccessible with bulk fluorescence anisotropy approaches, and anticipate that p-PALM is well-suited to explore numerous crowded cellular environments. In total, our findings indicate that the NPC’s internal organization consists of multiple dynamic environments with different local properties.

## eLife digest

Most of the genetic material inside an animal cell is enclosed within a compartment called the nucleus. This compartment is separated from the rest of the cell by the nuclear envelope, a double-membrane structure containing thousands of pores that selectively allow certain molecules (collectively referred to as cargo) to enter and exit the nucleus.

The movement of cargo through the pores is controlled by large groups of proteins called nuclear pore complexes. The pore is at the center of the complex and is filled by a selective barrier made of an extensive network of flexible proteins known as the FG-network. Other proteins known as nuclear transport receptors bind to the proteins in the FG-network and carry cargos through the barrier.

The properties of the nuclear pore barrier and how it rapidly selects the right cargos have been difficult to study, in part, because the barrier network is constantly changing and is crowded with hundreds of transport receptors. New techniques are needed to investigate such highly crowded environments inside cells. Now, Fu et al. use a technique called polarization photoactivated localization microscopy (p-PALM) to explore the molecular crowding within the nuclear pore barrier in human cells. This technique measures the freedom with which a single molecule embedded in the network can rotate, providing information about the local environment. In a crowded environment, it is harder for the probe molecule to rotate as it is more likely to bump into other molecules.

Fu et al. found that there are different levels of crowding within the barrier. This is consistent with previous ideas of how the pore barrier could work, which propose that the nuclear transport receptors are less tightly packed in the center of the FG-network. This enables transport receptor and cargo complexes to move more rapidly through the center of the pore. The molecular crowding in the barrier of nuclear pores parallels that observed in other cellular compartments that also rely on assemblies of proteins with flexible structures. Thus, future work using p-PALM is expected to reveal more details about the biophysical properties of nuclear pores as well as those of other structures inside cells.

## Introduction

Intracellular environments are highly crowded, with typical local macromolecular concentrations of ~80–400 mg/mL, and some cellular environments contain only ~50% water (Kuznetsova et al., 2014). Under crowded conditions, excluded volume effects and local interactions can change protein activities by over an order of magnitude compared with the 'dilute' solutions typically used for most in vitro studies (Aumiller et al., 2014). Crowded conditions can affect protein folding, structure, shape, conformational stability and dynamics, binding interactions, and enzymatic activity (Kuznetsova et al., 2014; Zhou et al., 2008). Biological polymers play central roles in generating a variety of crowded environments. For example, the polymers in mucus, the extracellular matrix, the cytoskeleton, the vitreous humor of the eye, and the Nuclear Pore Complex (NPC) produce complex environments that restrict diffusion and trap molecules (Leterrier, 2001; Lieleg and Ribbeck, 2011). In addition, the numerous distinct bodies/granules within the nucleus and the cytoplasm have been interpreted to form via a phase separation-like mechanism due to high local concentrations of self-cohesive nucleic acid and/or intrinsically disordered protein polymers (Aumiller et al., 2014; Toretsky and Wright, 2014). Characterization of the physical, structural, dynamical, and functional properties of these crowded environments remains challenging due to the dearth of appropriate tools that are needed to investigate the complexity and heterogeneity of these environments on the nanoscale.

One example of a crowded environment is the pore of the NPC, which mediates bidirectional traffic between the cytoplasm and the nucleoplasm of eukaryotic cells. The translocation passageway of the NPC is occupied by hundreds of intrinsically disordered polypeptides (Lim et al., 2008; Peleg and Lim, 2010; Suntharalingam and Wente, 2003), 50–100 nuclear transport receptors (NTRs) (Lowe et al., 2015; Tokunaga et al., 2008), and protein and nucleic acid cargo complexes moving in opposite directions. NTRs are classified as importins or exportins, reflecting their ability to carry cargos into or out of the nucleus, respectively (for reviews, see [Chook and Süel, 2011; Güttler and Görlich, 2011; Jamali et al., 2011; Stewart, 2007; Wente and Rout, 2010]). On the nuclear side, RanGTP promotes disassembly of NTR/cargo import complexes, freeing the cargo and allowing NTRs to diffuse back to the cytoplasm (Chook and Blobel, 2001; Izaurralde et al., 1997; Rexach and Blobel, 1995; Siomi et al., 1997). NTR/cargo/RanGTP export complexes are disassembled on the cytoplasmic side after GTP hydrolysis, which results from interactions with RanGAP and a Ran-binding protein (RanBP) (Bischoff and Görlich, 1997; Bischoff et al., 1994; Güttler and Görlich, 2011; Kutay et al., 1997a; Okamura et al., 2015). Many of these assembly and disassembly reactions are coordinated to occur at the cytoplasmic and nucleoplasmic exits of the NPC’s central pore (Sun et al., 2013; Sun et al., 2008). Exactly how cargo complexes are specifically recognized and yet rapidly migrate in milliseconds (Dange et al., 2008; Grünwald and Singer, 2010; Kubitscheck et al., 2005; Tu et al., 2013; Yang et al., 2004; Yang and Musser, 2006a) through the NPC’s crowded environment remains enigmatic.

NPCs are large (~60–120 MDa) structures with octagonal rotational symmetry. They are comprised of ~30 different nuclear pore proteins (nucleoporins, or Nups), each of which are thought to be present in an integer multiple of eight copies (Cronshaw et al., 2002; Fahrenkrog and Aebi, 2003; Mi et al., 2015; Ori et al., 2013; Rout and Aitchison, 2001). The vertebrate NPC has an outer diameter of ~120 nm, and extends ~200 nm along the transport axis (Fahrenkrog and Aebi, 2003; Stoffler et al., 1999). Eight flexible filaments extend ~50 nm into the cytoplasm, and an additional eight filaments extend ~75 nm into the nucleoplasm and terminate in a ring to form the nuclear basket (Fahrenkrog and Aebi, 2003; Stoffler et al., 2003). In humans, the hourglass-shaped central pore has a minimum diameter of ~50 nm and a length of ~85 nm (Maimon et al., 2012). Within this large pore and decorating its openings is a network of ~200–250 intrinsically disordered polypeptides, which generates a permeability barrier impeding macromolecular transport (Lim et al., 2008; Ori et al., 2013; Peleg and Lim, 2010; Suntharalingam and Wente, 2003) and which is particularly selective against larger cargos (Mohr et al., 2009; Popken et al., 2015; Ribbeck and Görlich, 2001; Timney et al., 2016). These disordered polypeptides contain, in total, 3000–4000 phenylalanine-glycine (FG) repeats to which NTRs transiently bind as they carry cargos through NPCs (Cronshaw et al., 2002; Denning et al., 2003; Rout et al., 2000; Strawn et al., 2004; Tran and Wente, 2006). We term this assembly of intrinsically disordered FG-containing polypeptides the FG-network.

Each FG-containing nucleoporin (FG-Nup) has a globular anchor domain that is embedded in or attached to the NPC scaffold, and thus, it acts as an anchor point for the flexible and mobile FG-domain. The FG-repeat motifs are separated by short (~10–20 amino acid residues), largely hydrophilic segments (Denning and Rexach, 2007; Yamada et al., 2010). The FG-domains do not form readily recognizable secondary structures, but rather are more appropriately described as flexible polymers with alternating hydrophobic and hydrophilic domains (Lim et al., 2006; Yamada et al., 2010). The FG-network is sufficiently fluid and mobile that it is rapidly displaced by transporting cargos, which can be up to ~40 nm in diameter (Frey and Görlich, 2009; Hough et al., 2015; Lim et al., 2007; Milles et al., 2015; Panté and Kann, 2002).

The ‘polymer brush’ (Lim et al., 2006; Peleg and Lim, 2010) and ‘hydrogel’ (Frey and Görlich, 2007; Frey et al., 2006) models are the most widely cited descriptions of the biophysical nature of FG-polypeptide assemblies. These models are two extremes in the model space describing the potential morphologies and properties of the FG-Nup assemblies within the NPC (Eisele et al., 2013; Vovk et al., 2016). The polymer brush model postulates that the FG-polypeptides are largely non-interacting (beyond steric repulsion), relatively extended and minimally entangled (Lim et al., 2006; Peleg and Lim, 2010), and their spatial assemblies are stabilized mostly by entropic forces (Vovk et al., 2016). The hydrogel model posits that the FG-polypeptides exhibit significant inter- and intra-strand cohesiveness via FG-FG interactions, which results in a connected dense network (Frey and Görlich, 2007; Frey and Görlich, 2009; Frey et al., 2006; Hülsmann et al., 2012). A hybrid, two-gate model postulates brush-like structures on both cytoplasmic and nuclear sides of the NPC, suitable for binding and (dis)assembly reactions, and a central cohesive structure in the center of the pore that provides the permeability barrier (Patel et al., 2007). The spatial distribution of functional activities in this two-gate model is supported by single molecule transport results (Sun et al., 2013; Tu et al., 2013). Quantitative modeling of FG-polypeptide behavior predicts a smooth transition between brush-like and gel-like behaviors in response to relatively small changes in physical properties and favors a picture intermediate between a brush and a gel (Vovk et al., 2016). The magnitude of the inter- and intra-chain cohesiveness that differentiates these two descriptions could be different for different FG-polypeptides, or different segments of the same FG-polypeptides, in distinct spatial locations within the NPC (Vovk et al., 2016). Avidity calculations indicate that the multivalent affinities of NTRs depend critically upon the local free FG-repeat concentration (Tu et al., 2013). In agreement with these predictions, experimental results indicate that some sub-populations of NTRs have very long dissociation times, and therefore, they potentially can form an integral part of the permeability barrier (Kapinos et al., 2014; Lowe et al., 2015; Schleicher et al., 2014). Taken together, these findings suggest that the FG-polypeptides and NTRs act together to form different local environments with different properties within the NPC (Coalson et al., 2015; Ghavami et al., 2014; Lowe et al., 2015; Eskandari Nasrabad et al., 2016; Osmanović et al., 2013; Tagliazucchi et al., 2013; Yamada et al., 2010).

Considering the uncertainty in the structural arrangement of and interactions between FG-polypeptides, and knowing that many tens to over a hundred macromolecules (including NTRs, Ran, and cargos) interact with the FG-network during steady-state transport (Abu-Arish et al., 2009; Lowe et al., 2015; Tokunaga et al., 2008), developing a general picture of FG-polypeptide distributions and local crowding conditions, and discerning their functional effects on cargo transport, is a challenging problem, but nevertheless essential for establishing the mechanism of nucleocytoplasmic transport and its implications. Here, we used the super-resolution approach photoactivated localization microscopy (PALM) (Betzig et al., 2006) to probe the locations of a number of FG-polypeptides and transport-related proteins within the NPC. Our main focus, however, was on using polarization PALM (p-PALM) (Gould et al., 2008) to measure rotational mobility, which is sensitive to local crowding conditions, and which enables probing of the local properties of crowded macromolecular assemblies that are currently inaccessible by other means. Crucially, we developed a theoretical model that enables detailed analysis of the experimental p-PALM data in terms of rotational diffusion constants. While numerous previous super-resolution approaches on NPCs utilized fixed samples, and most concentrated on scaffold structural questions (Löschberger et al., 2014, 2012; Lowe et al., 2015; Otsuka et al., 2016; Pleiner et al., 2016; Szymborska et al., 2013; Winterflood and Ewers, 2014), the NPCs in our samples were fully functional since our goal was to probe the properties of the FG-network, which is intrinsically dynamic. The results of our analysis of protein localization and local mobility within the NPC demonstrate that the FG-network is heterogeneous with regard to molecular crowding and that this can be influenced by the presence of embedded proteins, which argues for a remarkable complexity in nucleocytoplasmic trafficking pathways and their regulation.

## Results

### The polarization PALM (p-PALM) method

#### Motivation for the p-PALM method

The mobility of many molecules varies widely and often during their lifetime within cells, dependent on viscosity, crowding and local interactions. Most often measured is translational mobility, and numerous super-resolution light microscopy approaches have been developed over the past decade suitable for this purpose (see [Huang et al., 2010; Huang et al., 2009] for reviews). Highly localized effects, such as those produced by multiple binding interactions or increases in crowding, often produce small changes in translational mobility (nanometer-scale step sizes in milliseconds) that are very difficult, if not impossible, to detect by these methods. However, these environmental changes can produce significant and detectable changes in *rotational* mobility. We surmised that the rotational mobility of a probe within the FG-network of NPCs would be strongly influenced by the densities of the FG-polypeptides and other macromolecules, such as NTRs, that increase crowding and decrease mobility of the FG-polypeptides, and therefore, we developed a method that could detect differences in rotational mobility.

**Figure 1.**
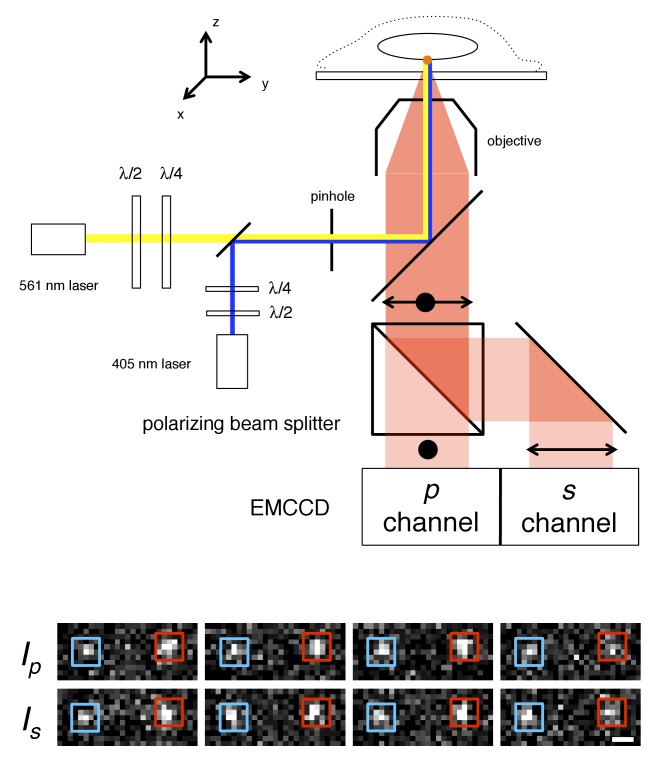
p-PALM imaging. The fluorescent protein mEos3 was photoactivated by UV illumination (405 nm), and excited by linearly or circularly polarized 561 nm light. The mEos3 fluorescence emission was separated by a 50:50 polarizing beam splitter and detected on two halves of an EMCCD camera. The images show four successive frame-pairs in which two molecules (*red* and *blue* boxes) of Pom121-mEos3 (see Figure 1—figure supplements 1,2 for all mEos3 fusion protein constructs used in this work) were detected at the bottom of the nucleus in a permeabilized HeLa cell using circular excitation (see Video 1). Fluctuating emission intensities (*I_p_* and *I_s_*) result from changes in the molecules’ average orientation during the image integration time (*t* = 10 ms). The λ/2 and λ/4 waveplates were used to rotate the angle of linear polarization and to adjust the ellipticity, respectively, of the excitation beams. A 300 µm pinhole was used to reduce the illumination area to ~7 µm (narrow-field epifluorescence [Yang et al., 2004]). Scale bar: 1 µm.

Our basic approach was to genetically attach a photoactivatable fluorescent protein to different FG-polypeptides, and at different locations within an FG-polypeptide, in order to determine the rotational mobility of this fluorescent probe in different environments within the FG-network. Fluorescence polarization measurements are often used for probing rotational motion and are readily applied at the single molecule level (Forkey et al., 2000; Forkey et al., 2005; Ha et al., 1999; Harms et al., 1999; Lakowicz, 2006; Loman et al., 2010; Testa et al., 2008). However, single molecule polarization measurements within the NPC pose a special challenge: since the NPC has eight-fold rotational symmetry, any Nup genetically tagged with a fluorescent protein (or chemically tagged with a dye) will be present in numerous copies, and therefore, due to their proximity, the diffraction-limited emission from individual fluorescent tags will overlap significantly, thereby complicating analysis. Consequently, we combined single molecule polarization measurements with PALM (Betzig et al., 2006), in which probe molecules are stochastically and individually photoactivated. In this approach, termed polarization PALM (p-PALM), single fluorescent protein molecules were activated as in PALM, but the emission was split by a polarizer onto separate halves of an EMCCD camera (Figure 1), enabling polarization measurements to be made on individual molecules. As we show, there are significant advantages of this single molecule approach over ensemble fluorescence polarization methods.

**Figure 1—figure supplement 1.**
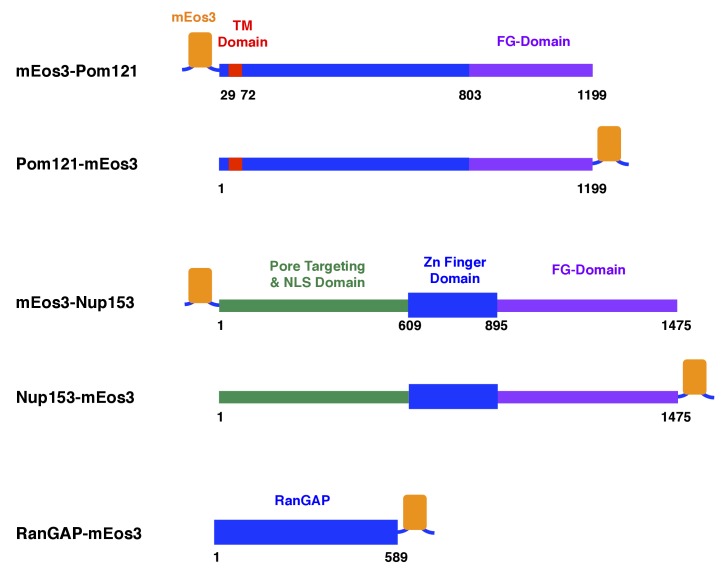
mEos3 fusion protein constructs with Pom121, Nup153, and RanGAP. Domain structures for Pom121 and Nup153 were adapted from (Söderqvist et al., 1997) and (Nakielny et al., 1999), respectively.

**Figure 1—figure supplement 2.**
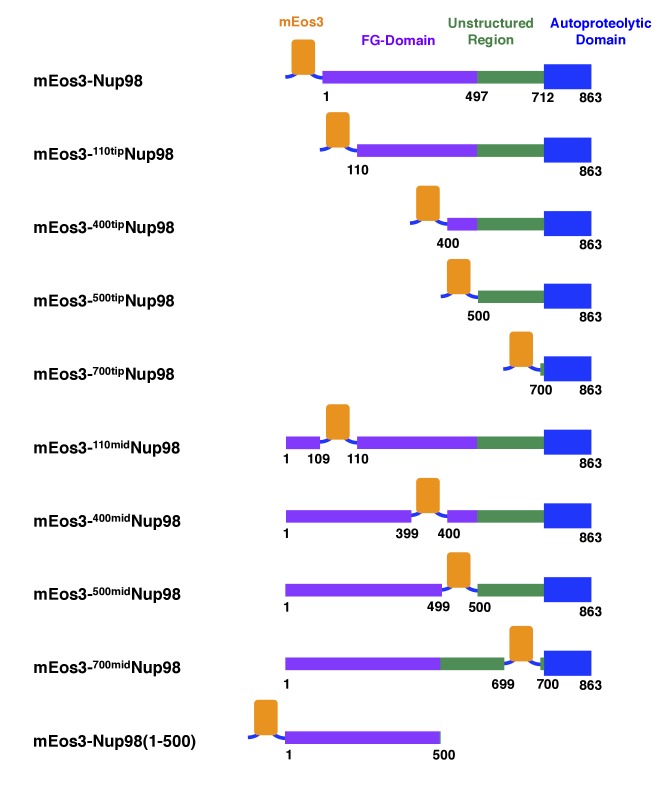
mEos3 fusion protein constructs with Nup98. Domain structure adapted from (Ren et al., 2010).

**Video 1.** p-PALM imaging of Pom121-mEos3 at the bottom of the nucleus. The top half is the *p*-channel and the bottom half is the *s*-channel. The round illumination area created by the narrow-field epifluorescence imaging is clearly detectable within the center of the fields. Fluorescent spots that appear and disappear arise from single mEos3 molecules and are clearly correlated between the two channels. *t* = 10 ms; 240 nm square pixels (see Figure 1).

Similar approaches to the p-PALM method described herein were used previously to detect changes in rotational mobility (Gould et al., 2008; Testa et al., 2008). However, a quantitative relationship between rotational diffusion constants and p-PALM measurements has not been reported. In our analysis, we explored the different rotational time regimes and now more fully describe the parameter space, which is essential to interpret the results and provides numerous additional insights into the power of the approach. Rotational random walk simulations were used to determine the effect of imaging speed, fluorescence lifetime, anisotropic rotational diffusion, dipole orientation, thresholding, noise level, and numerical aperture over ~10 orders of magnitude of the average rotational diffusion constant, *D_r_*. These simulations revealed that p-PALM can detect changes occurring on timescales that are largely inaccessible by other means. The details of the experimental approach and simulations are described in the **Materials and methods** section. An overview of the approach and the general results of our analysis are summarized in the following sections.

#### Outline of the p-PALM method

**Figure 2.**
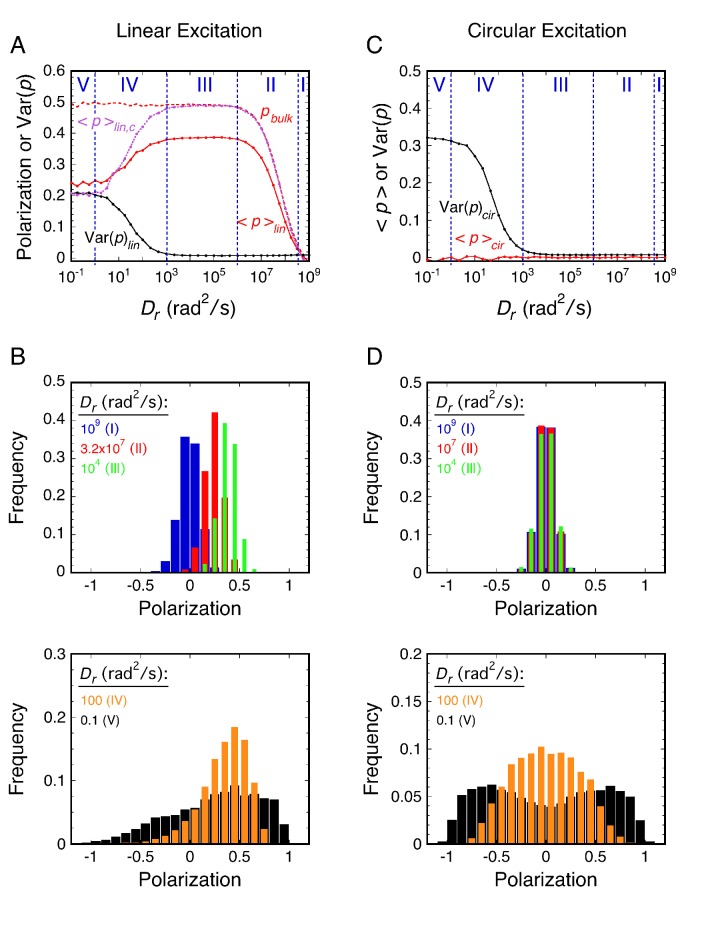
Principles of rotational mobility analysis by the p-PALM method. Rotational random walk simulations were used to obtain polarization histograms, and the corresponding mean polarization, <*p*>, and its variance, Var(*p*), for different *D_r_* values using linearly (**A** and **B**) and circularly (**C** and **D**) polarized excitation. The values predicted for single molecule measurements (*solid*) in **A** and **C** were calculated from polarization histogram data, such as that shown in **B** and **D**. The bulk polarization (*p*_bulk_) using linear excitation (**A**) was calculated assuming that the photons from all molecules (*N* = 10,000 per *D_r_* value) were collected simultaneously in the two polarization channels. <*p*>_lin,c_ is the mean polarization collected under p-PALM conditions corrected for the polarization mixing by the microscope objective. See text for discussion and **Materials and methods** for simulation details and definitions. In all panels: *D_r_* = *D_x_* = *D_y_ *= *D_z_* = (*D_x_* + *D_y_* + *D_z_*)/3 (i.e., a spherical particle); the noise (*p* photons, *s* photons) was (22, 15) and (15, 15) using linear and circular excitation, respectively; 1400 (circular) or 2800 (linear) rotational random walk steps per simulation, yielding an average of ~350 photons at high *D_r_* values (see Figure 2—figure supplement 5); 10,000 initial values per simulation; *t* = 10 ms; τ (fluorescence lifetime) = 3.5 ns; θ*_obj_* = 74.1°. The five rotational diffusion regimes (identified in **A** and **C**) are described in Figure 2—figure supplement 1. Figure 2—figure supplements 1–5 illustrate the influence of fluorescence lifetime, integration time, threshold, intensity shape factor, noise, ellipticity, and the number of collected photons on <*p*> and Var(*p*). Figure 2—figure supplement 6 illustrates the relationship between the number of measurements and the statistical measurement uncertainty. The anisotropy values, <*r*>_bulk_ and <*r*>_lin,c_, are shown in Figure 2—figure supplement 7 and described in **Materials and methods**.

The main principle of p-PALM experiments is that rotational mobility information is extracted not from the average bulk polarization, but from polarization measurements obtained from thousands of individual molecules that are pooled to generate polarization frequency histograms (Figure 2). The primary experimental readouts from these data are the average polarization, <*p*>, and the variance of the polarization distribution, Var(*p*). In addition, the overall shape of polarization histograms and photon scatterplots can provide additional clues as to the underlying physical constraints on the probe’s rotational mobility. Polarization was defined as *p* = (*gI_p_ – I_s_*)/(*gI_p_* + *I_s_*), where *I_p_* and *I_s_* are the fluorescence intensities measured for each single molecule spot in the two polarization channels, and *g* corrects for the different photon collection efficiencies of these channels (Gould et al., 2008; Harms et al., 1999). We used a measurement timescale (10 ms) comparable to the timescale of protein import by the NPC (Grünwald et al., 2011; Tu and Musser, 2011). For circularly polarized excitation, the average polarization (<*p*>_cir_) as well as the peak of the distribution are theoretically always 0 (for uniformly distributed dipole orientations), providing a convenient check on instrument alignment and calibration (see below and **Materials and methods**), but providing no information on rotational mobility. Instead, the histogram width, quantified as Var(*p*), provides an estimate of the rotational mobility, with increasing width corresponding to decreasing rotational mobility (lower *D_r_* values). Since slowly rotating molecules emit from distinct orientations during the data collection period, a wider range of polarization values are obtained for lower *D_r_* values, whereas rapidly rotating probes yield time-averaged polarizations near zero. For linearly polarized excitation, <*p*>_lin_ is almost always non-zero and was the parameter used for inferring rotational mobility in this excitation mode. The effects of the *D_r_* on histograms of polarization measurements, <*p>*, and Var(*p*) for a spherical particle are shown in Figure 2.

**Figure 2—figure supplement 1.**
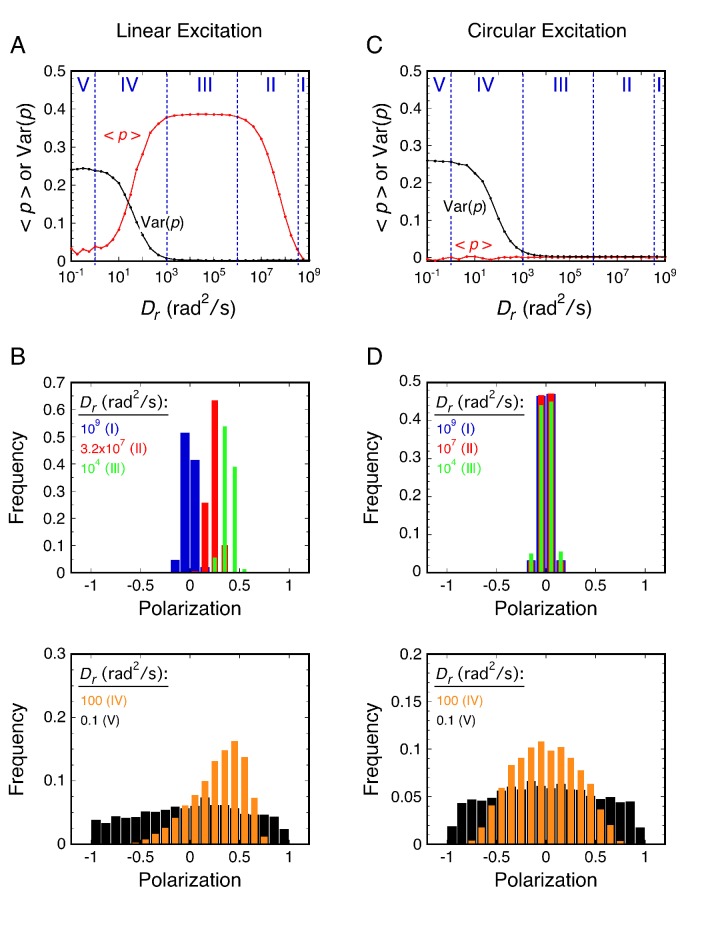
Principles of rotational mobility analysis by the p-PALM method – no threshold, intensity broadening, or background noise. Results of the same rotational random walk simulations used for Figure 2 using the photons collected in each channel with no corrections – no threshold selection was used, the intensity distribution was not broadened, and no noise was added (see **Materials and methods**). The contributions of each of these experimentally determined conditions on <*p*> and Var(*p*) are more explicitly illustrated in Figure 2—figure supplement 4. The five rotational diffusion regimes (identified in (**A**) and (**C**)) are characterized as follows. (*Regime I – very fast rotation*) During the excited state of the fluorophore, the transition dipole orientation is virtually completely randomized. All measurements of *p* from collecting hundreds of photons are therefore close to 0 for each molecule, and hence, the polarization histograms are narrowly centered around 0 for both excitation modes. (*Regime II – fast rotation*) The fluorescent particle rotates significantly during its fluorescence lifetime, but the dipole orientation when excited influences the direction (polarization) of the observed emission. Thus, emission is preferentially observed toward the direction of linearly polarized excitation. However, the transition dipole is completely randomized *between excitation events*. Therefore, polarizations measured using circularly polarized excitation remain closely centered around 0. (*Regime III – moderate rotation*) The fluorescent particle does not rotate significantly during its fluorescence lifetime, and thus, linear excitation leads to strongly biased emission, with <*p*>_lin_ ≈ 0.4. However, the transition dipole is essentially completely randomized between excitation events, and therefore, measured polarizations using circular excitation are closely centered around 0. (*Regime IV – slow rotation*) The transition dipole rotates between excitation events, but does not get completely randomized. Thus, individual polarization measurements are broadly distributed, with a width determined by the relationship between τ (is this the correct greek letter "tau"?) and *D_r_.* (*Regime V – very slow rotation*) The transition dipole does not rotate significantly between excitation events. This time regime corresponds to particles that are essentially ‘stuck’, and returns all polarizations for a particle randomly oriented in three-dimensions. The threshold significantly affects <*p*> and Var(*p*) under conditions where the molecules are essentially immobile (regime V). Under circularly polarized excitation, dipoles having a large axial component are largely invisible when there is a 100 photon threshold due to the low excitation probability by the beam propagating in the *z*-direction. This causes a central dip in the histogram for *D_r_* = 0.1 rad^2^/s with circular excitation (observed in Figure 2D, *black*, but not here in (**D**), due to the absence of a threshold), which increases Var(*p*)_cir_ (compare (**C**) with Figure 2C). With linearly *x*-polarized excitation under low *D_r_* conditions, molecules with negative polarizations are largely undetectable when there is a 100 photon threshold due to inefficient excitation (compare (**B**), *black*, with Figure 2B). The consequences of this threshold are an increase in <*p*> and a decrease in Var(*p*) (compare (**A**) with Figure 2A).

**Figure 2—figure supplement 2.**
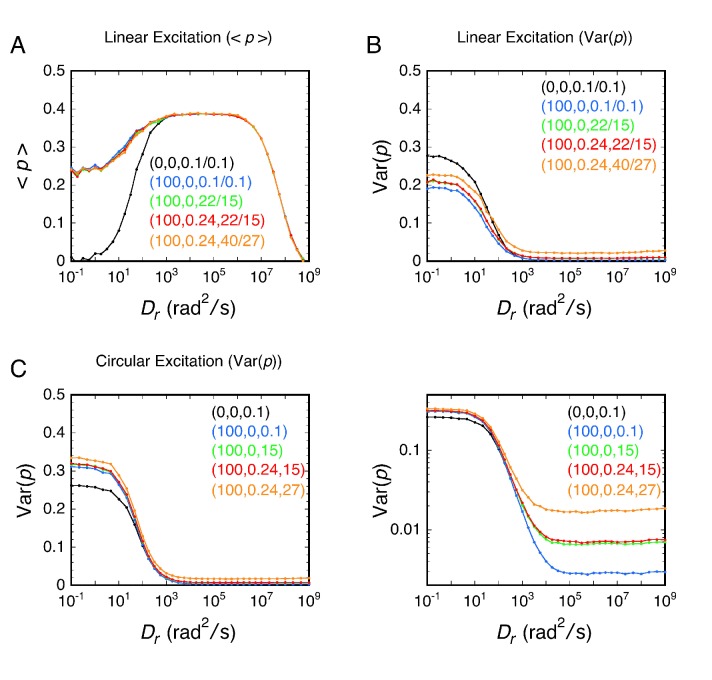
Effect of threshold, intensity shape factor, and background noise on <*p*> and Var(*p*). Effects on <*p>* and Var(*p*) are shown for the indicated parameter values (threshold, σ*, *p*-noise/*s*-noise) for a spherical particle. The <*p*>_cir_ was always 0, and therefore is not shown. Note that in (**C**), both linear (*left*) and log (*right*) ordinate scales are shown to illustrate the effect of thresholding and noise at low and high *D_r_* values, respectively. Threshold and noise values are given in photons. σ* is the shape parameter of the log-normal intensity distribution (see **Materials and methods**). The threshold has a major impact on <*p*>_lin_ (and a lesser impact on Var(*p*)_cir_) at low *D_r_* values and noise primarily influences the Var(*p*)_cir_ baseline at high *D_r_* values. The value of the shape parameter has no significant effect.

**Figure 2—figure supplement 3.**
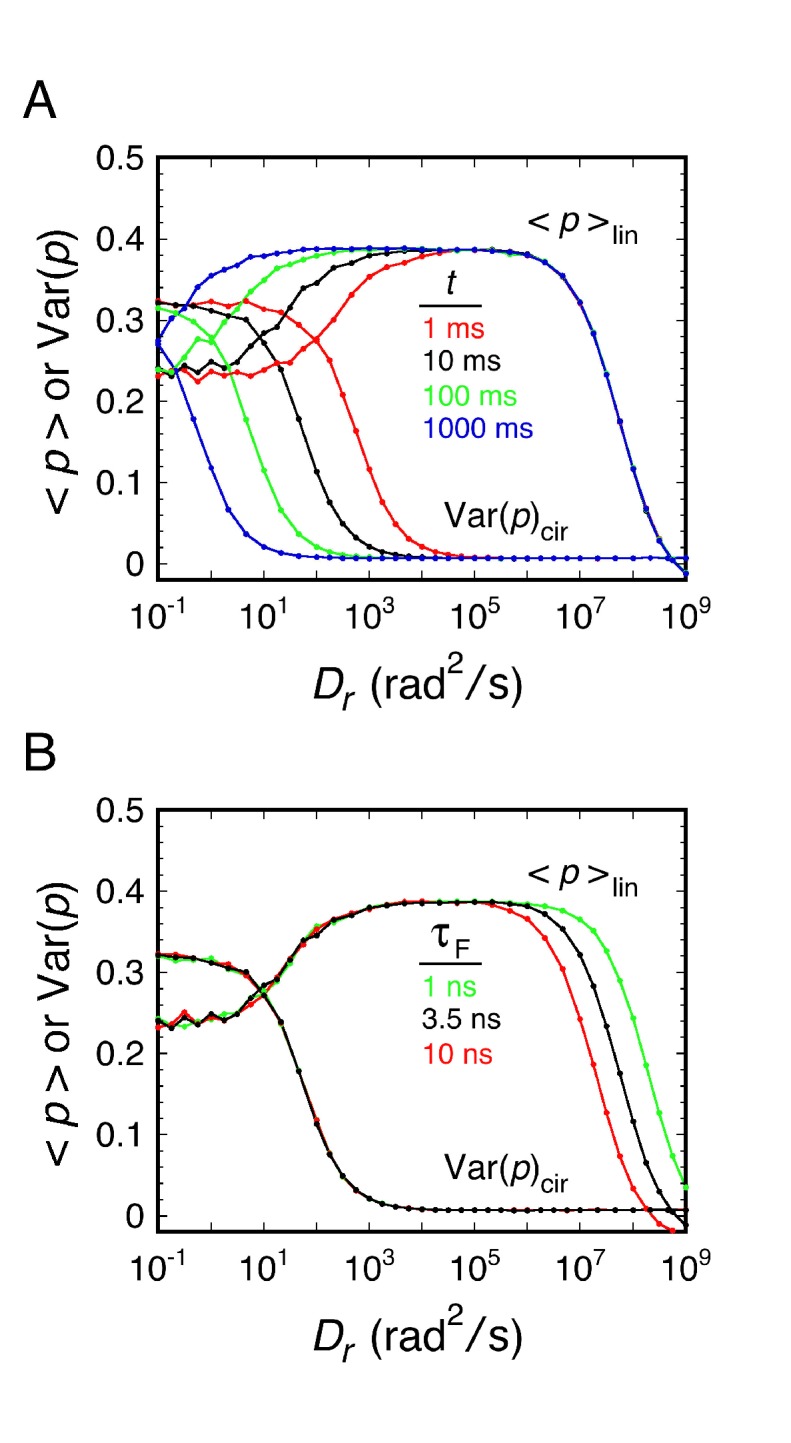
Effects of image integration time and fluorescence lifetime on <*p*>_lin_ and Var(*p*)_cir_. (**A**) Effect of image integration time (*t*). (**B**) Effect of fluorescence lifetime (τ_F_). These simulations were performed under the conditions of Figure 2 , unless otherwise noted. These simulations demonstrate that τ_F_ determines the position of Regime II and that *t* determines the position of Regime IV (see Figure 2—figure supplement 1 for the definition of the rotational mobility regimes).

**Figure 2—figure supplement 4.**
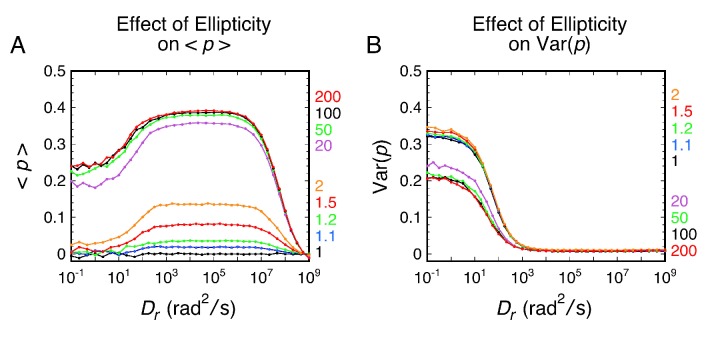
Effect of excitation ellipticity on <*p>* and Var(*p*). (**A**) Effect of ellipticity on <*p>*. (**B**) Effect of ellipticity on Var(*p*). Ellipticity (ε) values of ≤ 1.2 represent close to circular excitation; while ε = 1 represents true circular excitation, optical effects or misalignment cause deviations from the ideal case, which is difficult to achieve. For ε ≥ 20, the excitation beam is *p*-polarized. 1400 (ε ≤ 2) or 2800 (ε ≥ 20) rotational random walk steps (*N_steps_*) were used per simulation. Other parameters are as in Figure 2.

**Figure 2—figure supplement 5.**
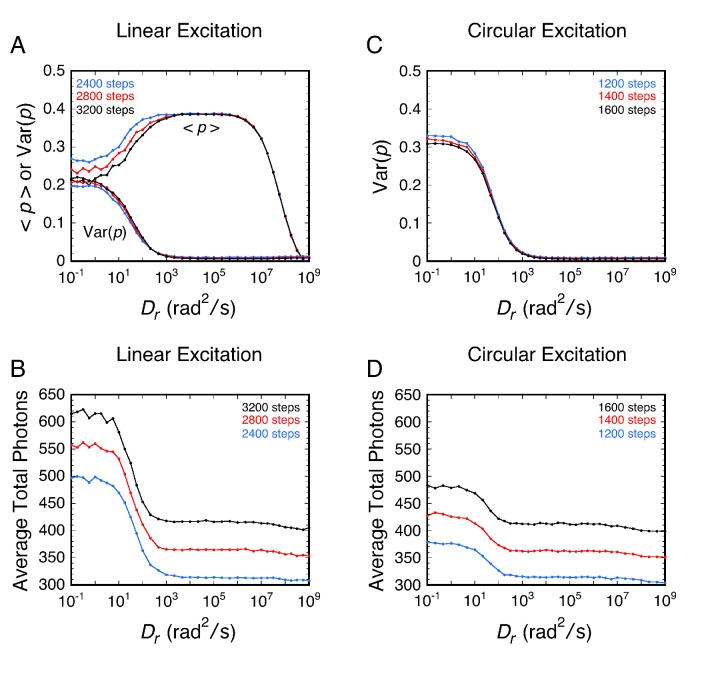
Influence of the total photons collected on <*p>* and Var(*p*). In the case of circular excitation, the expected total number of photons recovered at the limit of high *D_r_* is approximately given by *N_photons_* = *N_steps_* (1-cos(θ_obj_))/3. The expected total photons recovered using linear excitation is 2-fold lower, which is why double the number of steps were used for (**A**) and (**B**) relative to (**C**) and (**D**). The graph values at the high *D_r_* limits are higher than the formula predicts due to the baseline noise and the threshold (loss of information from < 0.5% of the molecules). The higher average total photons at low *D_r_* values occurs because the threshold eliminates low emitting molecules.

**Figure 2—figure supplement 6.**
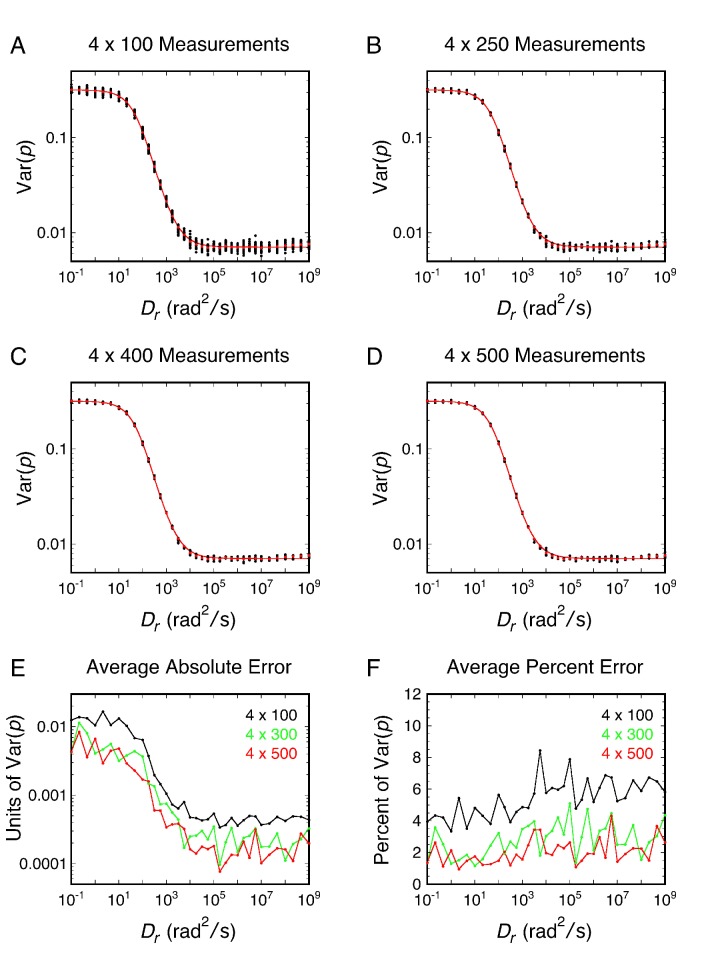
Estimating the error in datasets used to calculate Var(*p*)_cir_ – the number of data points required for reasonable estimates. (**A–D**) Variations in the Var(*p*)_cir_ for different sized datasets. Using the dataset in Figure 2C, estimates of Var(*p*)_cir_ were obtained by dividing the data into four equivalently sized subsets of *n* = 100–500 data points. For each 4 x *n* dataset, the mean and standard deviations (SD) of the four individual means were calculated. The *black points* are means calculated under the indicated conditions, the *red points* are the means calculated for the entire *N* = 10,000 dataset for each set of conditions, and the *red curves* are fits to the total dataset using Equation 25. (**E and F**) Absolute and percent errors under the indicated conditions. These calculations suggest that the Var(*p*)_cir_ value estimated from a p-PALM dataset of ~2000 points (4 × 500 points) differs from the ‘true’ value by an average of ~2–3%. The ‘true’ value was included in the range defined by the mean ± SD approximately 85% of the time, and thus this range defines the 85% confidence interval. In contrast, for a normal distribution, the mean ± SD should include the ‘true’ value approximately 68% of the time. Var(*p*)_cir_ is therefore not normally distributed, which violates the assumption of most statistical tests.

**Figure 2—figure supplement 7.**
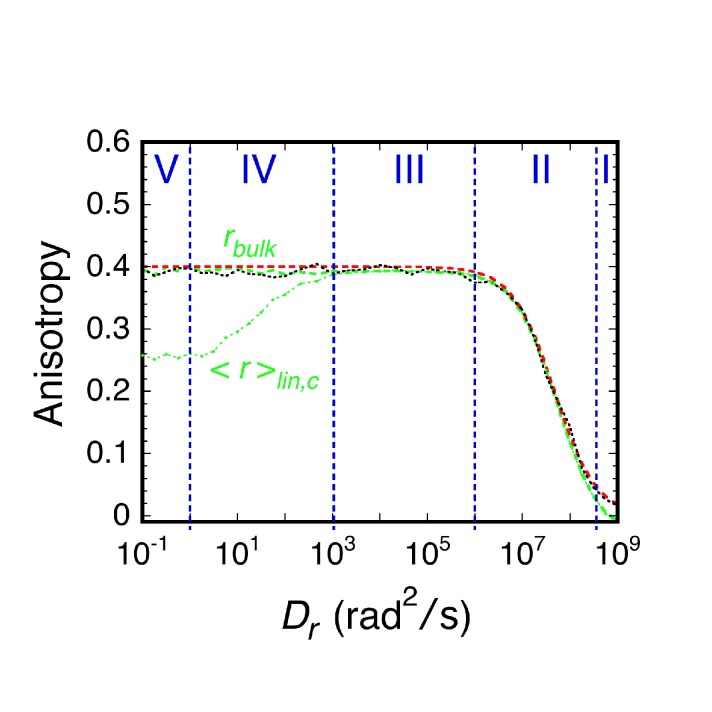
Comparison of bulk anisotropy with the corrected anisotropy calculated from p-PALM experiments. Bulk anisotropy (*r*_bulk_; *green dashed line*) and the corrected anisotropy from p-PALM experiments (<*r>_lin,c_; green dotted line*) are defined in Equations 22 and 24, respectively. The anisotropy calculated from the Perrin equation (*r* = *r*_0_/(1 + 6*D_r_*τ_F_; *red*) is shown for comparison (*r*_0_ is the fundamental anisotropy attained as *D_r_*τ_F_ → 0) (Lakowicz, 2006). The deviation between *r* (from the Perrin equation) and *r*_bulk_ (from simulations) at high *D_r_* values is a consequence of the large step sizes at the highest *D_r_* values, which can be corrected by using smaller time steps (*black dashed line; N* = ~20,000 photons, time step = 0.01 ns).

The monomeric fluorescent protein mEos3.1 (hereafter, simply denoted mEos3) (Zhang et al., 2012) was used as our probe molecule. Photoactivation of mEos3 by UV (405 nm) light results in conversion (photoactivation) from a ‘green’ (~500–550 nm emission) to an ‘orange’ (~570–650 nm emission) fluorescent state, a process that is irreversible due to polypeptide cleavage (McKinney et al., 2009; Wiedenmann et al., 2004). The mEos3 proteins were successively and individually photoactivated by continuous low level UV irradiation, and deactivated by photobleaching. PALM images and p-PALM polarization histograms were generated from thousands of position or polarization measurements, respectively, from many tens to hundreds of NPCs. For mEos3 tagged FG-polypeptides, the average number of probes detected per NPC was typically ~3–5. This is consistent with the known photoactivation efficiency of ~50% for photoactivatable proteins (Durisic et al., 2014). Narrow-field epifluorescence imaging (Yang and Musser, 2006b) was used for single molecule detection. In narrow-field epifluorescence, a small diameter excitation beam is confined to the center of the objective such that only a small area within the sample plane is illuminated (in our case, a 300 µm pinhole yielded an ~7 µm diameter illumination area; see Figure 1). This approach largely eliminated depolarization effects that normally result from focusing an excitation beam toward the *z*-axis (optical axis) by a high numerical aperture (NA = 1.46) objective (Ha et al., 1999; Olivini et al., 2001). The p-PALM approach does not require the high spatial precision typically obtained from super-resolution methods, which minimizes the need for high precision image alignment. Rather, polarization measurements are based on *intensities*, and spatial localizations are only necessary for spot correlation between the *p*- and *s*-channels. Thus, relatively low photon counts are acceptable. The excitation intensity was adjusted for all of our experiments so that the average total emission intensity was typically ~300–400 photons (*N_photons_*). While the number of photons collected from an individual molecule in the two imaging channels depends on the 3D orientation trajectory of the probe dipole during image acquisition, we emphasize that our method does not require knowledge of the individual 3D rotational trajectories of each probe. Instead, the average rotational mobility is inferred from the statistical properties of experimental polarization frequency histograms by comparison with the theoretically predicted values calculated from simulated rotational random walk trajectories (Figure 2 and **Materials and methods**).

#### Five rotational mobility regimes

Generally, for a given single molecule, the photons collected in a single image correspond to hundreds of excitation/emission cycles, during and between each of which the probe might rotate. Throughout this paper, we have assumed that the excitation and emission dipoles of the fluorescent particle are parallel (hereafter simply referred to as the transition dipole), which is the case for GFP and many fluorophores (Ha et al., 1999; Inoué et al., 2002). Our theoretical model of rotational diffusion (based on rotational random walk simulations – see **Materials and methods**) revealed five important rotational diffusion regimes in p-PALM experiments (Figure 2; more fully described and illustrated in Figure 2—figure supplement 1). These rotational diffusion regimes are defined by two time parameters, the fluorescence lifetime of the fluorophore (τ_F_) and the image integration time (*t*) (see Figure 2—figure supplement 2). The τ_F_ determines the time for the molecule to rotate between excitation and fluorescence emission. In combination with the number of photons collected, *t* determines the average time allotted for the molecule to rotate between potential excitation events (τ). In rotational random walk simulations, τ was calculated from *t* and the number of rotational walk steps (*N_S_*) as τ = *t*/*N_S_*, and was typically ~3-7 µs. Photon collection after each rotational walk step was dependent on excitation and emission probabilities of the dipole and the collection efficiency of the microscope channels. *N_S_* was set to approximately yield the experimentally collected number of photons (see **Materials and methods**). In general, the relationship between *D_r_* and <*p>* or Var(*p*) cannot be obtained analytically. However, our simulations of rotational diffusion were verified with an analytical solution for a special case (see **Materials and methods** and **Appendix 2**).

Considering the five rotational diffusion regimes (Figure 2), Regimes I and II correspond to the rotational mobility probed in most bulk fluorescence anisotropy experiments, where the increase in anisotropy at lower *D_r_* values is described by the Perrin equation (Figure 2—figure supplement 3) (Lakowicz, 2006). However, as demonstrated in the following sections, experimental polarization measurements from probes within the crowded FG-network of NPCs correspond to the slower rotational mobilities in Regimes III and IV. Linear excitation is primarily useful for molecules with high rotational mobility (Regimes I and II), where <*p*>_lin_ varies from 0 to ~0.4 (Figure 2). In single molecule experiments at lower *D_r_* values (Regimes IV and V), the fluctuations in <*p*>_lin_ strongly depend on thresholding and noise levels (Figure 2—figure supplement 4). Circular excitation is preferred over linear excitation for Regime IV (Figure 2) due to the larger dynamic range in Var(*p*)_cir_ and because this parameter is less sensitive to acquisition parameters than <*p*>_lin_ (Figure 2—figure supplement 4). Consequently, we primarily used circular excitation for the results that follow.

#### Differences between bulk and single molecule polarization measurements

Importantly, the single molecule average polarization (<*p*>_lin_) as defined in this paper and the average bulk fluorescence polarization (*p*_bulk_) obtained using linear excitation differ significantly, as illustrated in Figure 2A. There are two reasons for this. First, the intensities collected by a microscope objective are mixtures of parallel and perpendicular intensities. Correcting for these mixed intensities (see **Materials and methods**) yields <*p>*_lin,c_, which agrees with *p*_bulk_, except at low *D_r_* values (Figure 2A). And second, the remaining difference between <*p>*_lin,c_ and *p*_bulk_ that occurs at low rotational mobilities results from different weightings for each molecule’s fluorescence emission. That is, <*p*>_lin,c_ is calculated by weighting each molecule identically (i.e., the polarization of each molecule counts the same no matter how many photons are emitted), whereas *p*_bulk_ weights the contribution of each molecule to the measured polarization depending on the number of photons emitted. Consequently, since most polarizations are recovered in p-PALM experiments under low rotational mobility conditions, <*p*>_lin_ (and <*p>*_lin,c_) tends toward 0 at low *D_r_* values, although this decrease is limited by the sensitivity threshold. In contrast, dipoles oriented at large angles relative to the excitation polarization emit few photons, and therefore provide only a small contribution to *p*_bulk_. A mathematical description of the differences between p-PALM and bulk polarization measurements is given in **Materials and methods**. Notably, Var(*p*) cannot be obtained from bulk measurements because it requires polarization measurements from individual molecules, and hence, the quantification of slow rotational mobility made possible with p-PALM experiments cannot be obtained in a corresponding bulk experiment. Whereas anisotropy is favored as the measurement parameter for bulk fluorescence measurements, which typically have a parallel and two perpendicular channels (Lakowicz, 2006), polarization is a natural parameter for p-PALM measurements since there are only the two detection channels (*p* and *s*), neither of which can be used to directly account for the photons that escape detection.

#### *Effect of anisotropic rotation of the probe on* <*p*> and Var(*p*)

The approach taken in many of the experiments reported herein was to explore various local environments of the FG-network by covalently attaching an mEos3 probe to different FG-polypeptides and measuring its rotational mobility by p-PALM. Under such conditions, the principal rotational diffusion constants of the probe could be differentially affected relative to a freely diffusing spherical particle due to constraints generated by the local environment and/or by the FG-polypeptide to which it was tethered. We therefore used rotational random walk simulations to determine the effect of varying the relationship between the three principal rotational diffusion constants *D_x_*, *D_y_*, and *D_z_*. Somewhat surprisingly, our results and simulations suggested that the probe’s rotation mobility behavior was at most only slightly anisotropic or the angle between the dominant rotational axis and the transition dipole was near the magic angle of 54.7° (Axelrod, 1989). Consequently, the probe’s behavior largely resembled that of an untethered spherical particle. A more detailed discussion of the effects of rotational anisotropy on p-PALM measurements is given in **Appendix 1**.

#### Effect of numerical aperture (NA) on <*p*> and Var(*p*)

The NA is an important parameter in p-PALM experiments because it directly influences the experimentally measured values of <*p*> and Var(*p*) (Equations 7-11). Knowing the NA, <*p*> can in principle be standardized by converting the experimental value into the corresponding bulk parameter (Equation 21). However, there is no corresponding bulk value for Var(*p*). More importantly, the effective NA (NA_eff_) under the acquisition conditions can be significantly different than the nominal NA of the objective. For example, the spherical aberration that results from the refractive index mismatch when using an oil immersion lens for an aqueous sample yields a reduced NA, which is particularly significant when probing the sample far from the coverslip surface. A more detailed discussion of the effect of NA on p-PALM measurements is given in **Appendix 1**. Importantly, our major conclusions are not affected by a moderate uncertainty in the NA_eff_.

### p-PALM measurements of the FG-network of NPCs

#### Detecting rotational mobility changes in the FG-network of NPCs via p-PALM

For initial proof-of-concept experiments, mEos3 was fused to the C-terminus of Pom121 (Pom121-mEos3), and a stable HeLa cell line was generated. Pom121 is generally agreed to be a central membrane-integrated Nup (Antonin et al., 2005; Hallberg et al., 1993; Söderqvist and Hallberg, 1994; Söderqvist et al., 1997; Talamas and Hetzer, 2011; Yang and Musser, 2006a) with its N-terminal domain anchored to the NPC scaffold and its C-terminal FG-domain within the FG-network. We used both linearly and circularly polarized excitation beams (ellipticity >100 and <1.1, respectively), and p-PALM data were obtained from permeabilized HeLa cells by focusing on the bottom of cell nuclei. Thus, the sample plane coincided with the plane of the nuclear envelope (NE).

**Figure 3.**
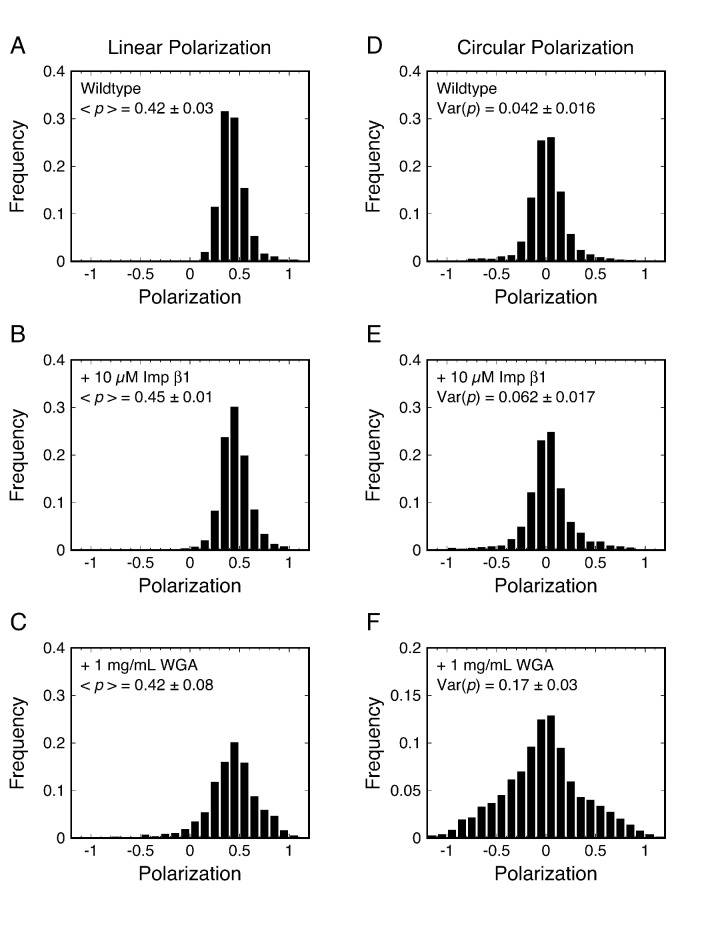
p-PALM polarization histograms for Pom121-mEos3. Linearly (**A–C**) and circularly (**D–F**) polarized excitation was used to obtain experimental polarization histograms for mEos3 attached to the C-terminus of Pom121 (Pom121-mEos3) under the indicated conditions. *t* = 10 ms. See text for discussion.

We first examined the as-isolated (wildtype) NPCs in permeabilized cells, and then determined whether addition of the NTR Importin β1 (Imp β1) or the transport inhibitor wheat germ agglutinin (WGA) (Adam et al., 1990; Dabauvalle et al., 1988; Finlay and Forbes, 1990; Wolff et al., 1988; Yoneda et al., 1987) influenced the rotational mobility of the mEos3 probe (Figure 3). Polarization frequency histograms using the collection/integration time *t* = 10 ms (per image) revealed that most measurements for Pom121-mEos3 were close to *p* = 0.4 for linearly polarized excitation and near *p* = 0 for circularly polarized excitation (Figure 3A and D). In the presence of Imp β1 (10 μM), the polarization histogram obtained with linear polarization was virtually unchanged and that obtained with circular polarization appeared slightly broader (Figure 3B and E). In the presence of WGA (1 mg/mL; ~26 μM), the difference between linear and circular excitation was clearly observed, with circular excitation producing a substantially wider polarization histogram (Figure 3C and F). WGA binds to O-GlcNAc-modified Nups, of which there are at least five in humans (Finlay and Forbes, 1990; Hülsmann et al., 2012). Since the WGA dimer has eight GlcNAc-binding sites (Schwefel et al., 2010), WGA molecules can potentially non-covalently ‘crosslink’ the FG-network, and thus severely inhibit the rotational mobility of proteins embedded within it. The data in Figure 3 support this hypothesis (compare with Figure 2). Overall, these data demonstrate that the p-PALM approach can detect changes in rotational mobility within the FG-network. A reduced rotational mobility likely implies an increased number of contacts with surrounding macromolecules (which could be strongly interconnected), and hence, we interpret a reduced rotational mobility as primarily a result of increased molecular crowding.

**Figure 3—figure supplement 1.**
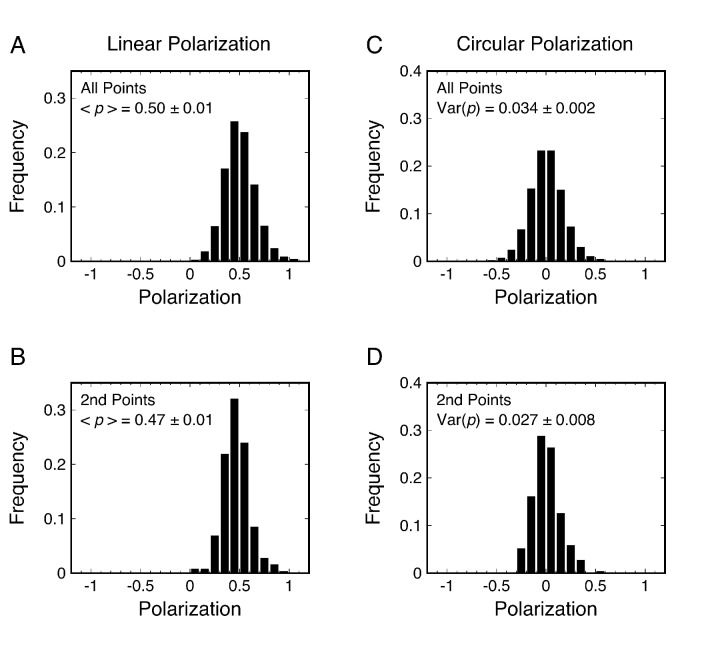
p-PALM polarization histograms for mEos3 in 92% glycerol. Linearly (**A–B**) and circularly (**C–D**) polarized excitation was used. For (**A**) and (**C**), all observed spots were included. For (**B**) and (**D**), only the second point in trajectories with ≥ 3 points were used – this process weights trajectories equivalently and insures that activation of the probe does not occur during the observation window (see Supplementary file 1). *t* = 10 ms.

#### Membrane topology of Pom121

**Figure 4.**
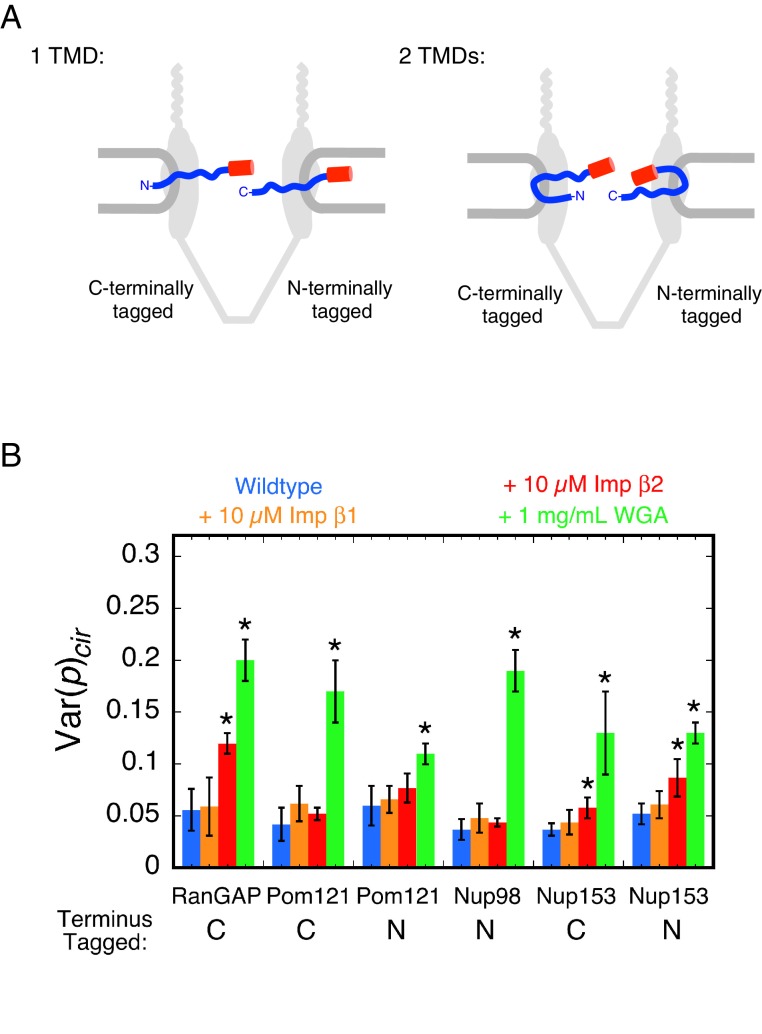
Pom121 membrane topology and rotational mobility of the mEos3 probe within the FG-network under various conditions. (**A**) Possible membrane topologies of Pom121. The N-terminus of Pom121 is predicted to be in the lumen of the ER (1 TMD) or in the central pore (2 TMDs). TMD = transmembrane domain. (**B**) Var(*p*)_cir_ values for various proteins under the indicated conditions. p-PALM data were obtained under the same conditions as in Figure 3. The stars (*) indicate significantly different values from the wildtype (*blue*) condition within the same group according to a two-sided Welch’s *t*-test (95%). Note that the actual significance level is expected to be higher than indicated since the error bars shown here are wider than a typical standard deviation (see Figure 2—figure supplement 6). A significant effect of WGA on the probe attached to the N-terminus of Pom121 suggests that this part of Pom121 is located within the central pore, and not in the ER lumen, and thus, that Pom121 has two TMDs (see (**A**)). 10 µM NTR ≈ 1 mg/mL.

Pom121 is one of three membrane proteins anchoring the NPC to the NE (Eriksson et al., 2004; Stavru et al., 2006), yet its membrane topology remains unresolved. We considered that the number of transmembrane domains (1 or 2) might be resolved by comparing the effect of WGA on the rotational mobility of the mEos3 probe attached to the N- or C-terminus of Pom121. Most studies describe Pom121 as having a single transmembrane domain (TMD) near its N-terminus, based primarily on a single long sequence of hydrophobic residues (Antonin et al., 2005; Funakoshi et al., 2011; Hallberg et al., 1993; Söderqvist and Hallberg, 1994; Söderqvist et al., 1997; Talamas and Hetzer, 2011). This topology would place the N-terminus of Pom121 in the perinuclear space (between the inner and outer NE membranes), and thus, inaccessible to exogenous reagents such as WGA. However, the hydrophobic transmembrane region of Pom121 is ~44 residues, and thus, it is long enough to span the membrane twice – a possibility that was considered in the initial report identifying this protein (Hallberg et al., 1993). In this alternate scenario, the N-terminus of Pom121 would be accessible from (or embedded within) the FG-network (Figure 4A), and the rotational mobility of an N-terminally attached probe could potentially be influenced by WGA.

To probe the membrane topology of Pom121, we attached mEos3 to the N-terminus of Pom121 (mEos3-Pom121), and obtained p-PALM measurements under wild-type, +NTR, and +WGA conditions after permeabilization of a stable cell line. WGA had a strong effect on the rotational mobility of the mEos3 probe (Figure 4B). These data support the hypothesis that Pom121 has two TMDs, and that the N-terminus of Pom121 is embedded within the FG-network. Alternatively, WGA could have long-range effects that are mediated across the NE membrane, thus reducing rotational mobility of mEos3 within the ER lumen, but we consider this scenario unlikely. These data demonstrate that the p-PALM approach can address structural questions difficult to ascertain with other methods by using the effects of increased crowding to report on the local environment of (or accessibility to) a probe.

#### Rotational mobility at different locations within the FG-network

Having established a framework for interpreting p-PALM measurements and demonstrating its utility for addressing structural questions, we then explored the rotational mobility of mEos3 tethered at various locations within the FG-network. We expected that different FG-polypeptide densities and different local concentrations of embedded NTRs and WGA would be reflected in differential effects on rotational mobility, and hence report on different levels of molecular crowding. In addition to the centrally located Pom121, we examined Nup98, a crucial element of the permeability barrier (Hülsmann et al., 2012). To probe the nuclear basket, we used Nup153 (Fahrenkrog et al., 2002; Hase and Cordes, 2003), which plays roles in both protein import and mRNA export and regulates the permeability barrier (Lowe et al., 2015; Makise et al., 2012; Shah and Forbes, 1998; Ullman et al., 1999). To probe the cytoplasmic filament region, we used RanGAP, which is essential for promoting GTP hydrolysis resulting in export complex disassembly (Bischoff and Görlich, 1997; Bischoff et al., 1994; Güttler and Görlich, 2011; Kutay et al., 1997a; Okamura et al., 2015) and which binds in its SUMOylated form to Nup358 (Reverter and Lima, 2005), a major component of the cytoplasmic filaments (Walther et al., 2002; Wu et al., 1995).

p-PALM measurements indicated that WGA had a strong effect on rotational mobility when the mEos3 probe was fused to all four proteins. In contrast, NTRs had a substantially weaker or no effect on probe rotational mobility (Figure 4B). Since both NTRs and WGA were added at ~1 mg/mL (~10 µM and 26 µM [dimer], respectively), these data indicate that exogenous NTRs introduce less molecular crowding in the FG-network structure than WGA.

#### Rotational mobility at different positions within the Nup98 disordered domain

**Figure 5.**
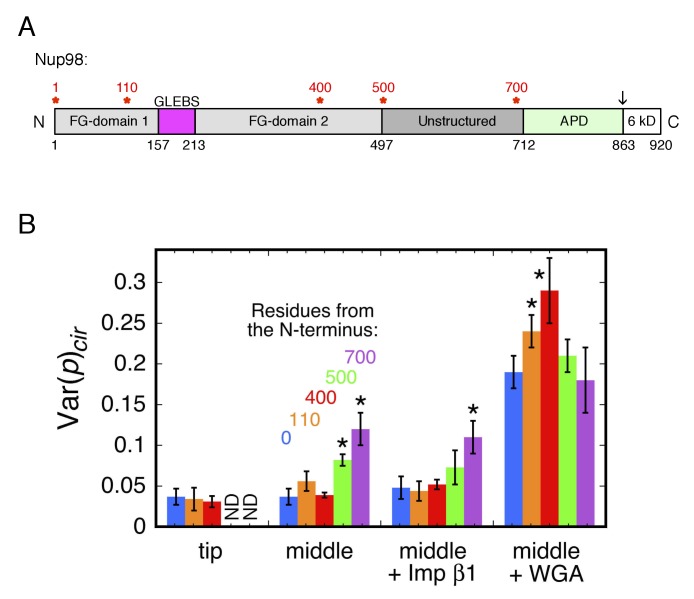
Rotational mobility of mEos3 at different positions within the Nup98 disordered domains. (**A**) Human Nup98 domain structure. The GLEBS domain is a binding motif for RNA export factors. The autoproteolytic domain (APD) co-translationally cleaves the C-terminal 6 kDa domain after residue 863 (arrow) (Rosenblum and Blobel, 1999). Amino acids numbers are indicated on the bottom and stars (*) indicate the positions at which the mEos3 probe was incorporated (0, 110, 400, 500, 700 residues from the N-terminus). The regions from residues 1–157 and 213–712 are considered disordered. Adapted from (Ren et al., 2010). (**B**) Var(*p*)_cir_ values obtained under the indicated conditions for the mEos3 probe at different positions within the Nup98 disordered domains (see **A**). p-PALM data were obtained as in Figure 3. The stars (*) indicate significantly different values from the wild-type (*blue*) condition within the same group according to a two-sided Welch’s *t*-test (95%). Note that the actual significance level is expected to be higher than indicated since the error bars are wider than a typical standard deviation (see Figure 2—figure supplement 6).

Different segments of an FG-polypeptide are expected to sample different regions of the FG-network depending on its anchoring point and the polypeptide length from the anchor point. To probe the molecular crowding in the neighborhood of different segments of an FG-polypeptide, we performed p-PALM measurements on mEos3 probes attached at different positions within the disordered region of Nup98. We chose Nup98 because of its important role in forming the permeability barrier (Hülsmann et al., 2012). The domain structure of Nup98 is shown in Figure 5A. With the exception of a 56-residue GLEBS domain, the first ~700 residues of this 920 residue protein are largely disordered, and most of this region contains FG repeats. The C-terminal autoproteolytic (APD) domain of Nup98 binds to Nup88 and Nup96, and is therefore thought to anchor Nup98 onto the NPC scaffold (Griffis et al., 2003; Hodel et al., 2002; Pleiner et al., 2016; Stuwe et al., 2012).

**Figure 5—figure supplement 1.**
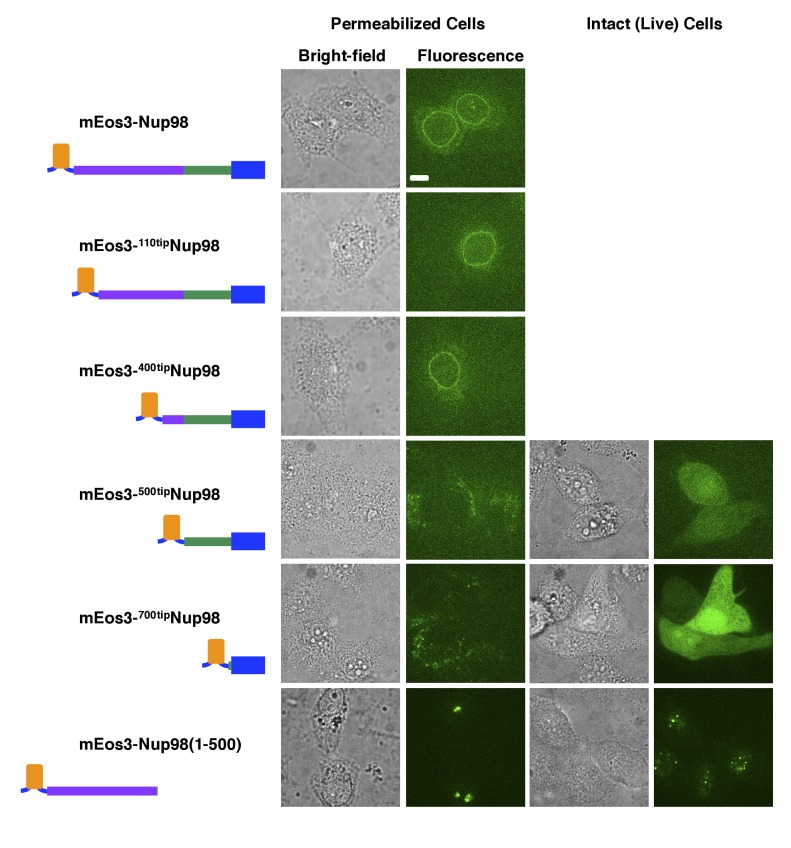
NE binding of Nup98 tip mutants. The mEos3-Nup98, mEos3-^110tip^Nup98, and mEos3-^400tip^Nup98 proteins localize to the NE. The mEos3-^500tip^Nup98, mEos3-^700tip^Nup98, and mEos3-Nup98(1-500) proteins do NOT localize to the NE. These data indicate that both the FG-domain (*purple*) and the C-terminal domain (*green + blue*) are required for Nup98 to bind to the NPC. Fusion protein schematics are colored as in Figure 1—figure supplement 1. For the proteins indicated here, stable cell lines were only generated for the mEos3-Nup98 fusion protein (see **Materials and methods**) – the remainder were examined after transient transfections. Bar = 10 µm.

In the first set of experiments, 109, 399, 499, or 699 residues were deleted from the N-terminus, and the mEos3 probe was attached to the new N-terminus (Figure 1—figure supplement 1). These ‘tip’ labeled, truncated versions of Nup98 were then examined in p-PALM experiments after transient transfections (Figure 5B). To our surprise, mEos3-^500tip^Nup98 and mEos3-^700tip^Nup98 were not retained at the NE in permeabilized cells (Figure 5—figure supplement 1), indicating that the Nup98 C-terminal domain was insufficient to anchor these proteins to the NPC scaffold (it is unclear if any binding to the NE occurs in live cells; see Figure 5—figure supplement 1). In separate experiments, we found that residues 1–500 of Nup98 do not bind to the NPC in either permeabilized or live cells (Figure 5—figure supplement 1), indicating that both the FG and C-terminal domains of Nup98 appear to be required for localization to the NPC.

In a second set of experiments, we added the deleted portion back to the Nup98 ‘tip’ mutants, thus placing the mEos3 probe in the middle of two sections of Nup98. These ‘middle’ labeled Nup98 mutants (Figure 1—figure supplement 1) were all localized to NPCs. In as-isolated permeabilized cells, rotational mobility was reduced when the mEos3 probe was attached near the Nup98 C-terminal folded domain. These data suggest more crowded conditions near the NPC scaffold than at the tip of Nup98 (which is likely found most of the time toward the center of the pore or on the nucleoplasmic and cytoplasmic sides, i.e., near the ‘surface’ of the FG-network). Imp β1 did not further affect rotational mobility relative to the as-isolated cells, yet WGA had a significant effect on the probe at all positions (Figure 5B). Notably, WGA had the strongest effect on the rotational mobility of the probe on mEos3-^400mid^Nup98, indicating that larger WGA-induced effects occurred near the middle of the Nup98 disordered region rather than near its FG-polypeptide tip (N-terminus) or the anchor domain (APD domain). These data indicate that different segments of an FG-polypeptide domain experience different environments within the FG-network.

#### Mixed populations

In all our p-PALM experiments, the mEos3 probe was attached to a single location on the protein of interest. Nonetheless, different probe molecules could potentially be in different environments of the FG-network due to the length and flexibility of the FG-polypeptide to which they were attached and/or variable environments within the same or different NPCs. The presence of a mixed population is not obviously apparent in values of <*p*> and Var(*p*), since these are obtained by averaging over the population. However, polarization frequency histograms and scatterplots of the photons recovered in the *p-* and *s*-channels (photon scatterplots) both can provide evidence for populations of probes with distinct rotational mobilities. In both cases, the distributions obtained from simulations of molecules with different rotational mobilities can be combined and compared with the experimental data to test/verify the mixed population hypothesis.

**Figure 6.**
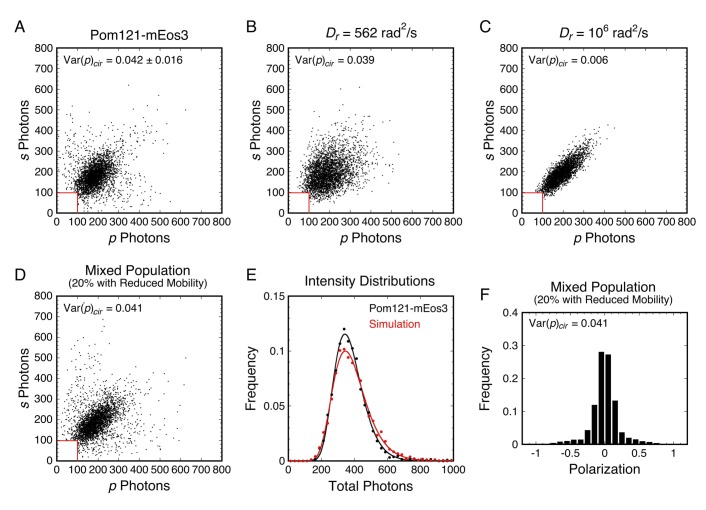
Analysis of rotational mobility within mixed populations. (**A**) Scatterplot of the numbers of photons collected in the *p*- and *s*-polarization channels for Pom121-mEos3 (data from Figure 3D; *N* = 3463). Each dot corresponds to one molecule. (**B and C**) Simulated photon scatterplots under the indicated conditions, assuming the NA_eff_ = 1.02 in water (θ_obj_ = 50°; see Appendix 1—figure 4). The results in (**B**) were simulated with the value of *D_r_* corresponding approximately to the Var(*p*)_cir_ in (**A**), whereas (**C**) corresponds to a higher rotational mobility at the high end of Regime III. The scatter is significantly wider in (**A**) than either (**B**) or (**C**), suggesting a mixed population. (**D**) Simulated photon scatterplot for a mixed population consisting of 80% of molecules with *D_r,1_* = 3160 rad^2^/s and 20% with *D_r,2_* = 100 rad^2^/s. Despite a relatively flat Var(*p*)_cir_ curve in Regime III, the scatterplots become narrower as *D_r_* increases from 10^3^ to 10^6^ rad^2^/s (Figure 6—figure supplement 3). The prevalent rotational mobility in the population was chosen guided by the width of the central scatter in (**A**) compared with the scatterplots in Figure 6—figure supplement 3. (**E**) Total photon intensity histograms of the experimental results in (**A**) and the mixed population simulation in (**D**), fit to a log-normal distribution. (**F**) Polarization histogram from the results in (**D**) (compare with Figure 3D). These results support the hypothesis that the p-PALM data for Pom121-mEos3 arise from a mixed population. For appropriate visual comparison with the experimental dataset in (**A**), *N* ≈ 3500 for all simulations. The *red box* near the origin identifies the region eliminated by the 100 photon threshold. Figure 6—figure supplements 1–8 show additional experimental and simulated photon scatterplots, and the effect of γ under highly anisotropic conditions on polarization histograms.

There were multiple indications that the mEos3 probes in some of our p-PALM experiments sampled multiple environments distinguishable by different rotational mobilities. One example is shown in Figure 6. The broad wings of the polarization histogram in Figure 3D were not expected (see Figure 2D). Moreover, a photon scatterplot for Pom121-mEos3 reveals a substantially wider distribution of points (Figure 6A) than simulated scatterplots that assume a homogeneous population (Figure 6B and C). In contrast, a model that assumes a mixture of two sub-populations of molecules having distinct *D_r_* values yields good agreement with the experimental data (Figure 6D–F). This example demonstrates that evidence for a mixed population can be obtained from both the polarization histogram and the photon scatterplot of a given dataset. Therefore, for more accurate interpretation, the underlying data should be more carefully examined rather than relying on the summary values <*p*> and Var(*p*). **Appendix 1** contains further discussion and additional evidence for mixed populations within our p-PALM datasets.

**Figure 6—figure supplement 1.**
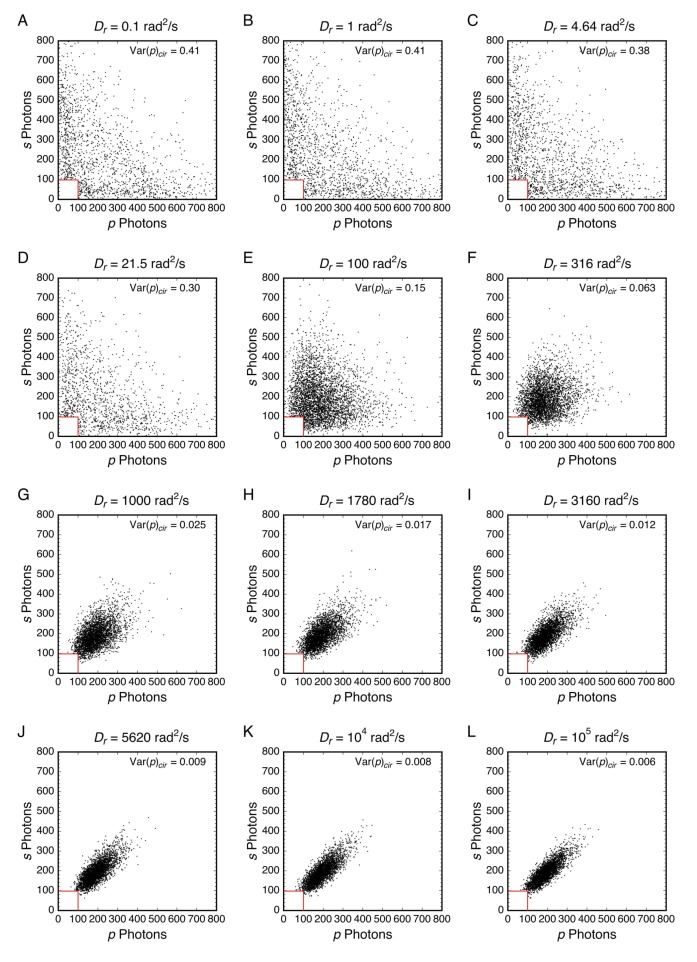
Simulated photon scatterplots using circular excitation. Over a range in Var(*p*)_cir_ values that are difficult to distinguish experimentally (~10^3^–10^5^ rad^2^/s), the widths of photon scatterplot distributions clearly narrow as *D_r_* increases (see also Figure 6B and C). As the width of the scatter for *D_r_* = 3160 rad^2^/s (**I**) reasonable approximates the central part of the distribution in Figure 6A, this rotational diffusion constant was assumed to be the dominant rotational mobility for the mixed population in Figure 6D. Data from Appendix 1—figure 4 for θ_eff_ ≈ 50° (NA_eff_ = 1.02).

**Figure 6—figure supplement 2.**
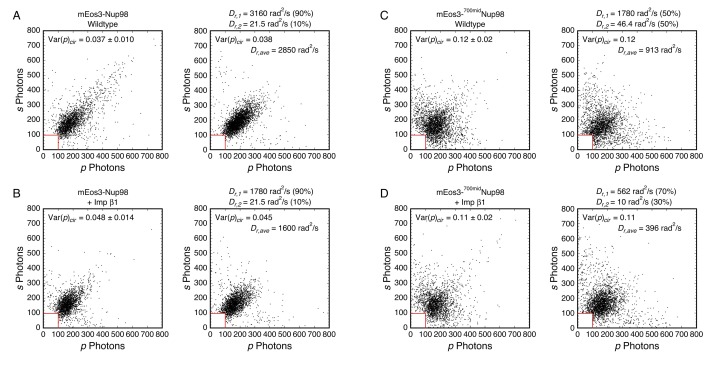
Mixed rotational mobility populations for mEos3-Nup98 and mEos3-^700mid^Nup98. Experimental (*left*) and simulated (*right*) photon scatterplots were generated under the indicated conditions. (**A**) Wild-type mEos3-Nup98 (*N* = 2242) and simulated scatterplot (*N* = 2455; 2846 steps → *N_photons_* ≈ 400). (**B**) mEos3-Nup98 + 10 µM Imp β1 (*N* = 2180) and simulated scatterplot (*N* = 2416; 2439 steps → *N_photons_* ≈ 350). (**C**) Wild-type mEos3-^700mid^Nup98 (*N* = 2411) and simulated scatterplot (*N* = 2396; 2439 steps → *N_photons_* ≈ 350). (**D**) mEos3-^700mid^Nup98 + 10 µM Imp β1 (*N* = 2840) and simulated scatterplot (*N* = 2800; 2439 steps → *N_photons_* ≈ 350). For all simulations, θ_obj_ = 50° (NA_eff_ = 1.02; see Appendix 1—figure 4). The average weighted rotational diffusion constant was defined as *D_r,ave_* = *f_1_D_r,1_* + *f_2_D_r,2_*, where *f_1_* and *f_2_* are the fractional sub-populations.

**Figure 6—figure supplement 3.**
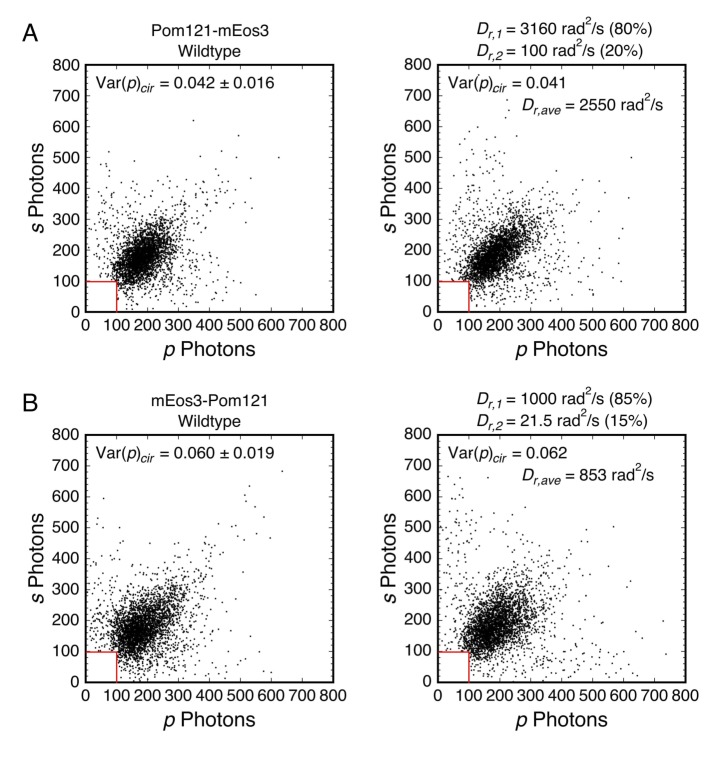
Mixed rotational mobility populations for mEos3-tagged Pom121. Experimental (*left*) and simulated (*right*) photon scatterplots were generated under the indicated conditions. (**A**) Wild-type Pom121-mEos3 (*N* = 3464) and simulated scatterplot (*N* = 3461). (**B**) Wild-type mEos3-Pom121 (*N* = 3561) and simulated scatterplot (*N* = 2913). For easier comparison between the two fusion proteins, the plots in (**A**) are from Figure 6A and D. For all simulations, θ_obj_ = 50° (NA_eff_ = 1.02; see Appendix 1—figure 4). See Figure 6—figure supplement 2 for the definition of *D_r,ave_*.

**Figure 6—figure supplement 4.**
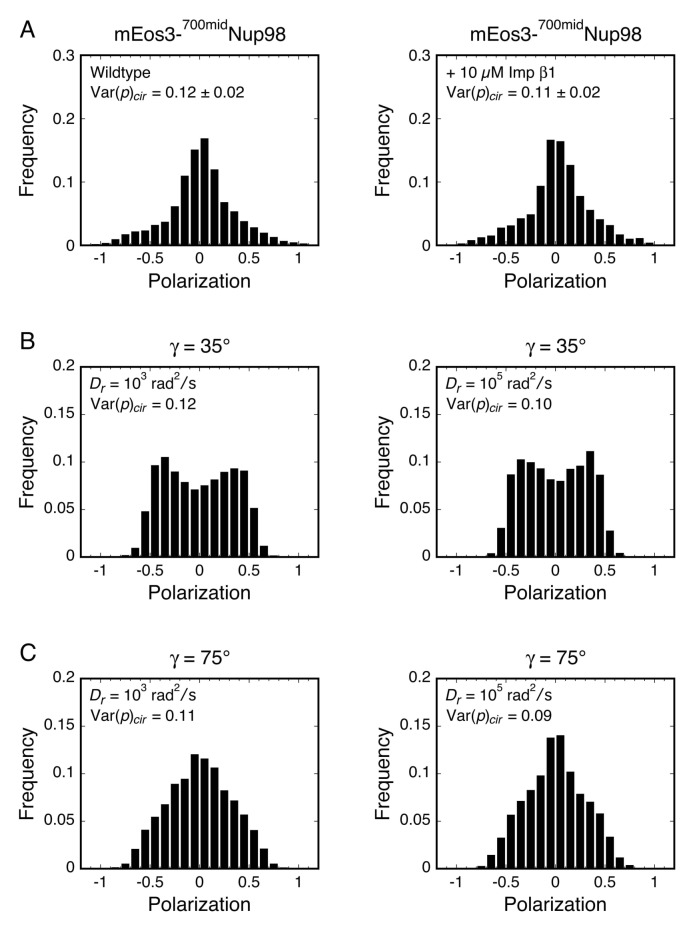
Polarization histograms for γ far from the magic angle for *D_z_*/*D_xy_* = 10^5^. (**A**) Polarization histograms for mEos3-^700mid^Nup98 under wt and +Imp β1 conditions (see Figure 5B). (**B and C**) Simulated polarization histograms for γ = 35° and 75° for *D_z_*/*D_xy_* = 10^5^ at low and high *D_r_* values (see Appendix 1—figure 2). Despite the similar Var(*p*)_cir_ values for the histograms in (**B**) and (**C**) to those in (**A**), the shapes are significantly different, indicating that the γ and *D_z_*/*D_xy_* constraints are inconsistent with the experimental results.

**Figure 6—figure supplement 5.**
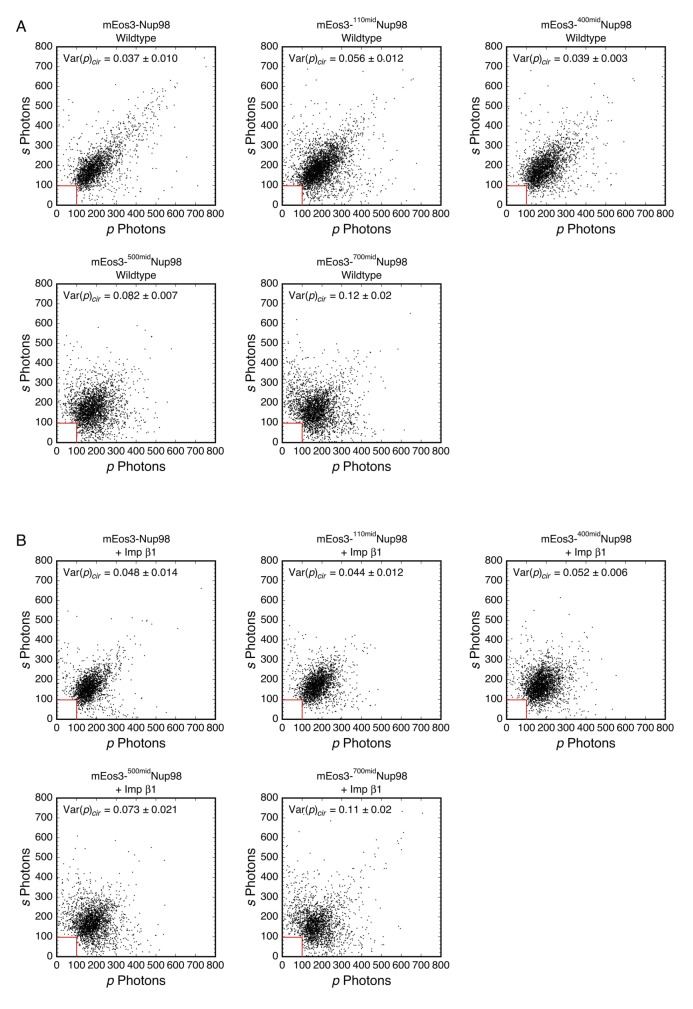
Photon scatterplots for data in Figure 5B (wt and +Imp β1). (**A**) mEos3-Nup98 middle mutants with no additions. (**B**) mEos3-Nup98 middle mutants + 10 µM Imp β1.

**Figure 6—figure supplement 6.**
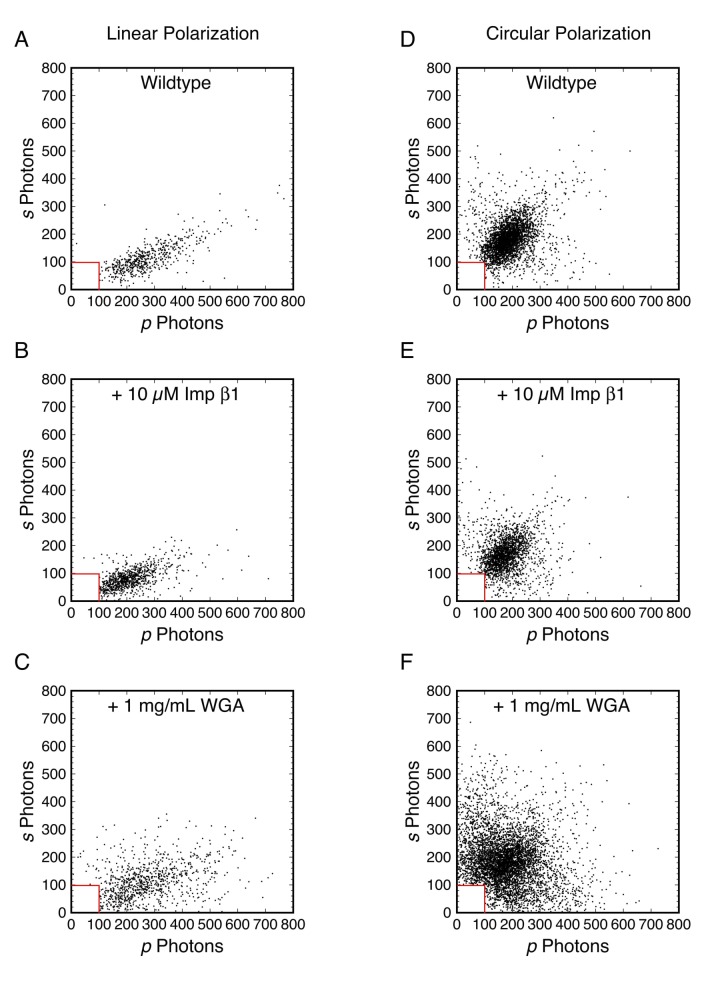
Photon scatterplots for the data in Figure 3. (**A**)-(**F**) correspond to Figure 3A–3F.

**Figure 6—figure supplement 7.**
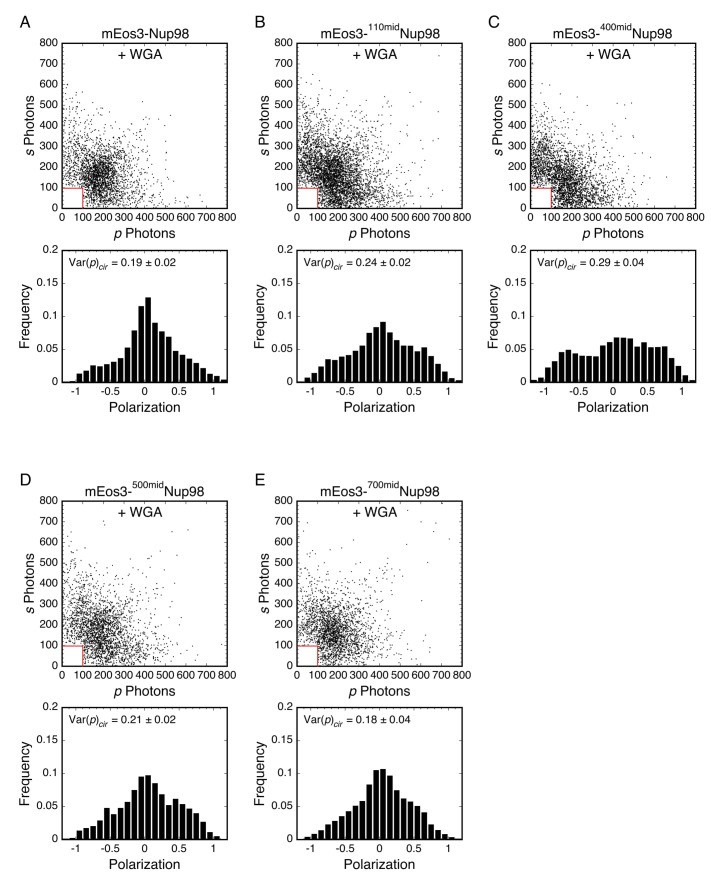
Photon scatterplots and polarization histograms for data in Figure 5B (+WGA). mEos3-Nup98 middle mutants + 1 mg/mL WGA.

**Figure 6—figure supplement 8.**
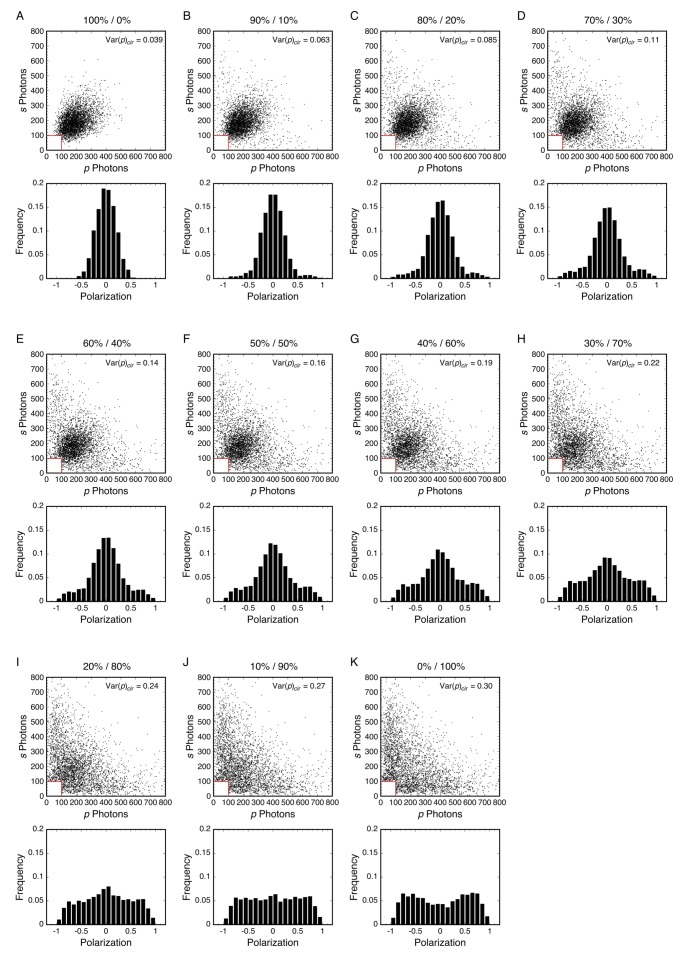
Mixed rotational mobility populations modeling mEos3-Nup98 middle mutants + WGA. Simulated photon scatterplots and polarization histograms are shown for mixed populations, assuming *D_r,1_* = 562 rad^2^/s (first %) or *D_r,2_* = 21.5 rad^2^/s (second %). Comparison with Figure 6—figure supplement 7 suggests that the experimental p-PALM data for the mEos3-Nup98 middle mutants with 1 mg/mL WGA can be explained by a mixture of two populations with different rotational mobilities. For all simulations, θ_obj_ = 50° (NA_eff_ = 1.02; see Appendix 1—figure 4). *N* = 3369–3959; *N_photons_* = 389–437.

### PALM of the FG-network of NPCs

#### Spatial distribution of mEos3 probes within the FG-network

**Figure 7.**
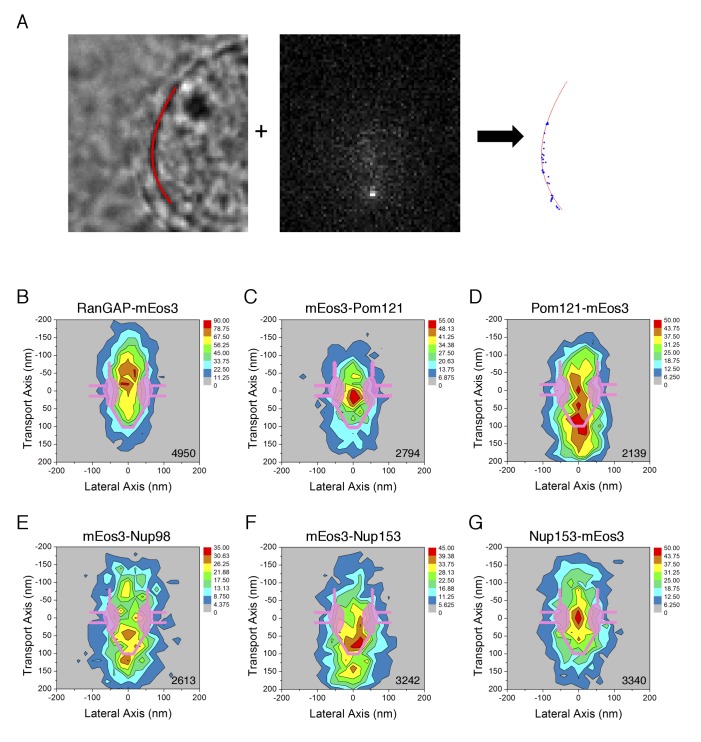
PALM 2D distribution maps of the mEos3 probe within the FG-Network attached to various proteins. (**A**) PALM imaging approach. (*left*) Nuclear envelope (NE) position. The position of the NE (*red*) was determined from a bright-field image at the nuclear equator, as described previously (Yang and Musser, 2006b). (*middle*) PALM imaging using circular excitation. In this example, the fluorescence from RanGAP-mEos3 was determined as in p-PALM imaging, except that a polarizer was not used and a single image per timepoint was collected (see Video 2). (*right*) mEos3 locations vis-à-vis the NE. The position of mEos3 in PALM spots was determined by 2D Gaussian fitting, and mEos3 positions (*blue*) were overlaid onto the NE position (*red*). (**B**)-(**G**) 2D particle distribution maps generated from PALM data. Spots/trajectories from different NPCs in PALM images (**A**) were aligned and overlaid, and two-dimensional (2D) distribution maps were generated by quantifying the number of localizations in a 400 nm x 400 nm area (20 nm x 20 nm ‘pixels’; see **Materials and methods** for details). The total number of spots/map is indicated in the bottom right corner (from 3 to 6 cells; 6–10 NPCs/cell).

To assist with the interpretation of the rotational mobility data and verify that the probe-labeled proteins were properly incorporated into NPCs, the locations of the mEos3 probe within the FG-network were examined via PALM. NEs were examined at the nuclear equator, allowing us to obtain localization information vis-à-vis the transport axis (Figure 7A). More than 2100 mEos3 localizations were used to generate a 2D density distribution map for each of the constructs used in Figure 4B (Figure 7B–G). Although previous reports have observed the eight-fold rotational symmetry of NPCs using super-resolution approaches (Löschberger et al., 2014; Löschberger et al., 2012; Ori et al., 2013; Pleiner et al., 2016; Szymborska et al., 2013), we found it difficult and unreliable to obtain radial distribution maps (when the optical axis was aligned with the transport axis) due to the low number of mEos3 probes detected per NPC (average of ~3–5).

**Figure 7—figure supplement 1.**
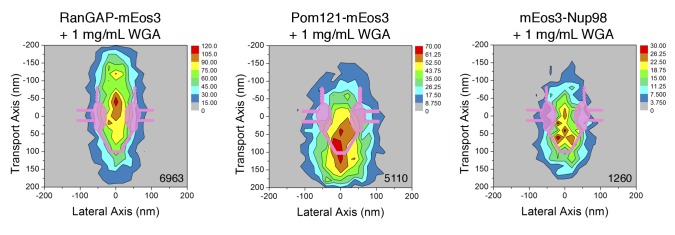
2D distribution maps of the mEos3 probe within the FG-Network in the presence of WGA. PALM data were collected as in Figure 7, except in the presence of 1 mg/mL WGA. The total number of spots/map is indicated in the bottom right corner (from 3 to 6 cells; 6–10 NPCs/cell).

**Video 2.** PALM imaging of RanGAP-mEos3 at the nuclear equator. Imaging conditions were the same as for p-PALM, except that there was no polarizer and only a single image was collected per time interval. Fluorescent spots that appear and disappear arise from single mEos3 molecules. *t* = 10 ms; 240 nm square pixels (see Figure 7A).

We caution that the lateral dimension was artificially compressed in these PALM density distributions. These maps were generated by determining the position of the NE, and aligning clusters of spots based on their centroids. This procedure positioned the centroids of all clusters on the central axis of the NPC. This approach was necessary since there were at most a few tens of spots per NPC, and we had no independent marker for the NPC scaffold. Thus, while the axial dimension was calibrated based on the NE position, the lateral dimension was artificially squeezed. Consequently, probes on the periphery of the pore may appear to be centrally distributed in these PALM density maps. In particular, while the probes on the N-terminus of Pom121 appear to be in the center of the pore (Figure 7C), we consider this to be a consequence of the alignment procedure.

The observed locations vis-à-vis the transport axis for the mEos3 probe attached to the FG-polypeptides generally agreed with previous published results. The mEos3 probe on the N-terminus of Pom121 yielded a distribution pattern along the transport axis that peaked within the central pore (Figure 7C), consistent with a short polypeptide segment anchoring it to the NPC scaffold. In contrast, the mEos3 probe on the C-terminus of Pom121 was more widely distributed (Figure 7D), consistent with access to a large region of the FG-network due to the length of the FG-polypeptide (Hallberg et al., 1993; Söderqvist and Hallberg, 1994). The mEos3 probe on the N-terminus of Nup98 was widely distributed (Figure 7E), consistent with previous studies that found Nup98 within the central pore and on both the nuclear and cytoplasmic sides of the NPC (Chatel et al., 2012; Frosst et al., 2002; Griffis et al., 2003; Krull et al., 2004; Radu et al., 1995). The N-terminus of Nup153 was predominantly localized to the nuclear basket, whereas its C-terminal end was predominantly localized closer to the central pore (Figure 7F and G). These results are consistent with previous antibody domain mapping studies on Nup153 (Chatel et al., 2012; Fahrenkrog et al., 2002; Krull et al., 2004; Lim et al., 2007).

In contrast to the results with the mEos3 probe on FG-polypeptides, which agreed with previous reports, the PALM map for RanGAP was a bit unexpected. RanGAP was predominantly localized in the cytoplasmic filament region, consistent with it being bound to Nup358 in its SUMOylated form (Hutten et al., 2008; Mahajan et al., 1997; Matunis et al., 1998; Reverter and Lima, 2005; Wälde et al., 2012). However, there were a surprising number of localizations within the central pore and nuclear basket regions (Figure 7B), suggesting that the cytoplasmic filaments penetrate into the central pore, and/or that RanGAP can bind to other parts of the FG-network.

### Combining PALM and p-PALM to probe for spatially distinct regions of varying rotational mobility

The relatively wide particle distribution maps observed in PALM experiments (Figure 7) suggested that regions of different rotational mobilities could potentially be resolved by combining the 2D localization maps generated via PALM with p-PALM rotational mobility information. This combined approach is challenging for the following reasons, which significantly reduced the size of current datasets. First, all p-PALM experiments reported thus far were performed by imaging the bottom of the nucleus, yielding spots from an approximately planar (2D) distribution of NPCs. In contrast, in order to obtain the position of the NE, PALM experiments were performed at the nuclear equator, yielding spots from a pseudo-linear (1D) distribution of NPCs. Thus, in combined PALM/p-PALM experiments, the NE was imaged at the nuclear equator, limiting the number of NPCs that could be simultaneously examined. Second, whereas entire trajectories were used for PALM, a single image per molecule was used for p-PALM to avoid biasing the data (see **Materials and methods**). This p-PALM constraint was retained in combined PALM/p-PALM experiments. And third, while *xy* spatial information was readily obtained from p-PALM fluorescent spots, localization precision was reduced in combined PALM/p-PALM experiments compared with typical PALM data since the emission intensity was distributed over two images (partially compensated by increasing the excitation intensity). Nonetheless, we demonstrate here that PALM localizations can be combined with p-PALM measurements.

**Figure 8.**
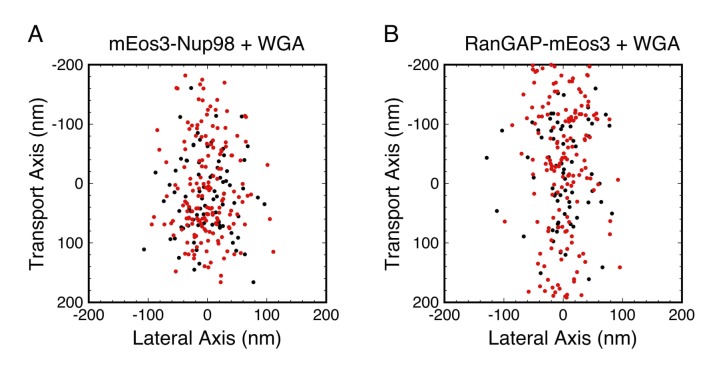
Combined PALM and p-PALM measurements of mEos3-Nup98 and RanGAP-mEos3 in the presence of WGA. Data were collected as in Figure 7, except that the emission was divided into *p*- and *s*-polarization channels. Each dot corresponds to one image from one probe molecule. (*red*) 1.2 > |*p*_cir_| > 0.3, which is only expected for slowly rotating molecules with *D_r_* < ~10^3^ rad^2^/s; (*black*) |*p*_cir_| < 0.3, which could be observed for molecules with any *D_r_* (see Figure 2C and D). The two color-coded populations have similar broad distributions in each panel, suggesting that WGA influences rotational mobility throughout most, if not all, of the FG-network. Total number of molecules and distribution widths (mean ± SD along the transport axis): (**A**) *N* = 359, (*black*) 17 ± 65 nm, (*red*) 5 ± 77 nm; (**B**) *N* = 386, (*black*) −8 ± 84 nm, (*red*) 21 ± 117 nm.

In order to explore the distribution of WGA-binding sites, we focused on the following question: did WGA reduce rotational mobility throughout the FG-network? We examined mEos3-Nup98 and RanGAP-mEos3, both of which yielded probe localizations widely distributed throughout the FG-network (Figure 7). The data were divided into those with |*p*_cir_| > 0.3, which is only expected for molecules with *D_r_* < ~10^3^ rad^2^/s, and those with |*p*_cir_| < 0.3, which could be observed for molecules with any *D_r_* value (Figure 2C and D). Thus, if WGA reduced rotational mobility in a specific region of the FG-network, the two datasets should yield spatially distinguishable distributions. This was not observed (Figure 8). Therefore, the data support the hypothesis that WGA inhibits rotational mobility throughout most, if not all, of the FG-network.

## Discussion

In this study, we have developed a combined experimental and theoretical framework for inferring the local rotational mobility of macromolecules in crowded environments using p-PALM. The p-PALM method was used to examine the macromolecular crowding in the vicinity of mEos3 probes positioned at different locations within the FG-network of NPCs, and PALM was used to determine the spatial distributions of these probes. Our major findings are: (1) different FG-polypeptides and different domains within the same FG-polypeptide experience different environments that are distinguishable by a probe’s rotational mobility; (2) in some cases, the binding of NTRs can increase crowding, thus producing significant differences in the properties of the local environments; and (3) WGA strongly influences rotational mobility throughout the FG-network, demonstrating that the local properties of the FG network can be modulated by embedded macromolecules. The implications of these findings provide a substantially improved understanding of the complexities of the FG-network, which we now discuss.

The average positions vis-à-vis the transport axis for the mEos3 probe attached to FG-polypeptides are largely consistent with previous results, as indicted in the **Results** section. Thus, the PALM data support the hypothesis that these mEos3-tagged proteins behave similar to their wild-type counterparts, and therefore, they enable probing of the FG-network. The mEos3 probe on the N-terminus of Nup98 was widely distributed along the transport axis with localizations within the central pore region as well as relatively far from the NPC center on both the nuclear and cytoplasmic sides (Figure 7E). This broad distribution pattern is consistent with Nup98 anchoring sites on the inner and outer ring structures of the NPC scaffold (Kosinski et al., 2016; Lin et al., 2016) and on the cytoplasmic filaments and nuclear basket (Frosst et al., 2002; Stuwe et al., 2012), which together agree with the high copy number (48) for Nup98 (Lin et al., 2016; Ori et al., 2013). While the probe on the C-terminus of Pom121 also yielded a broad spatial distribution pattern, consistent with the long C-terminal FG-domain, labeling of the Pom121 N-terminus yielded a narrower distribution, consistent with anchoring of this part of the protein at the NE (Figure 7C and D). Two anchoring sites for Pom121 via its N-terminal domain to Nup155 and/or Nup160 on both the inner and outer ring complexes (Kosinski et al., 2016; Lin et al., 2016) is consistent with a stoichiometry of 16 copies/NPC (Ori et al., 2013), although such dual anchoring is unresolvable at our current resolution.

For RanGAP, there were a surprising number of localizations within the central pore and basket regions (Figure 7B), seemingly inconsistent with the cytoplasmic distribution expected considering the known binding site for SUMOlyated RanGAP on the cytoplasmic filaments (Hutten et al., 2008; Mahajan et al., 1997; Matunis et al., 1998; Reverter and Lima, 2005; Wälde et al., 2012). RanGAP has a role in heterochromatin assembly (Nishijima et al., 2006), it has both nuclear localization and nuclear export signals (Feng et al., 1999), and, although found predominantly at the NE, it is also found in both the cytoplasmic and nucleoplasmic compartments (Mahajan et al., 1997; Matunis et al., 1998). These data suggest that RanGAP trafficks through the NPC, which could result in trapping within the FG-network during cell permeabilization. Alternatively, RanGAP could have additional roles within the FG-network other than export complex disassembly on the cytoplasmic filaments. Notably, RanGAP is not expected to catalyze disassembly of RanGTP-containing export complexes without a RanBP (Bischoff and Görlich, 1997; Bischoff et al., 1994; Güttler and Görlich, 2011; Kutay et al., 1997a; Okamura et al., 2015), thus suggesting that only the portion of RanGAP attached to the cytoplasmic filaments (RanBP2) may be active (Mahajan et al., 1997; Matunis et al., 1998; Wu et al., 1995; Reverter and Lima, 2005 #688; Yokoyama et al., 1995).

The goal of our p-PALM approach was to identify regions of increased crowding within the FG-network and thus map the protein density distribution within the pore. While attaching the mEos3 probe to the end or to the middle of an FG-polypeptide could potentially introduce severe anisotropy in the rotational diffusion constants, the entirety of our results and simulations suggest that the probe’s rotation mobility behavior is at most only slightly anisotropic or the angle between the dominant rotational axis and the transition dipole is near the magic angle. In either case, the probe’s behavior largely resembled that of an untethered spherical particle. This was a somewhat surprising finding. However, this conclusion greatly simplifies the interpretation of p-PALM data since it substantially limits the parameter space that needs to be considered.

Since the mEos3 probe’s rotational mobility behavior resembled that of an isotropic particle, comparison of the experimental <*p*>_lin_ and Var(*p*)_cir_ values with the simulation results for a spherical particle (Figure 2) enables the rotational mobility of the mEos3 probe under different conditions to be interpreted in terms of the approximate values of the average rotational diffusion coefficient (*D_r_*). Assuming an mEos3 fluorescence lifetime > ~3 ns, which is true for most fluorescent proteins (Bajar et al., 2016; Moeyaert et al., 2014), the <*p*>_lin_ values for all the conditions tested (Supplementary file 1) indicate that *D_r_* ≤ ~10^6^ rad^2^/s (Figure 2 and Supplements). Since *D_r_* for mEos3 free in solution is ~10^7^ rad^2^/s (calculated for a sphere [Loman et al., 2010]), the experimental <*p*>_lin_ values suggest that rotational mobility was reduced by at least an order of magnitude by crowding within the FG-network. Var(*p*)_cir_ values of ~0.2–0.3 (Supplementary file 1) for the mEos3 probe under some conditions, in particular in the presence of WGA, indicate that the *D_r_* was reduced to <~100 rad^2^/s (Figure 2 and Supplements), that is, at least a 5 orders of magnitude change in rotational mobility from the free particle (for at least a fraction of the particles in the population, considering that most populations likely consisted of particle distributions with multiple *D_r_* values – see **Appendix 1**). The p-PALM method therefore enables detection of large changes in rotational mobility. Moreover, since the p-PALM technique measures the polarization of individual molecules, which allows calculation of the variance of the polarization, it permits discrimination between rotational diffusion behaviors at much lower *D_r_* values than traditional anisotropy approaches, in which fluorescence depolarization is governed by the fluorescence lifetime (Lakowicz, 2006). Notably, using a bulk fluorescence anisotropy approach on yeast NPCs (Atkinson et al., 2013; Mattheyses et al., 2010; Kampmann et al., 2011), it was reported that some GFP probes were oriented when attached to some FG-polypeptides, particularly when they were near to the NPC scaffold. As the anisotropy signals were weak relative to homogeneous models, it appears that either the percentage of oriented molecules was low, or the orientation bias was weak. For either explanation, the assumption that probes were initially isotropically oriented in our random walk simulations is valid in most cases and leads to only minor errors in other cases. The fact that WGA had substantial effects on rotational mobility, as we observed here, and yet had very little, if any, effect on probe orientation (Atkinson et al., 2013) emphasizes the different physical parameters measured by the p-PALM and bulk fluorescence anisotropy approaches.

Under wild-type conditions, <*p*>_lin_ and Var(*p*)_cir_ values suggest that the *D_r_* was 10^3^–10^6^ rad^2^/s for the large majority of probe molecules, consistent with the high mobility expected for the FG-polypeptides and a dynamically flexible FG-network. Conditions that decreased the rotational mobility of the mEos3 probe have been interpreted to result from an increase in macromolecular crowding. A high density of macromolecules reduces molecular motion (Dix and Verkman, 2008; McGuffee and Elcock, 2010), presumably through an increased number of contacts with surrounding macromolecules. An mEos3 probe molecule within the FG-network can interact with FG-polypeptides, embedded macromolecules, or both. While the parameter Var(*p*)_cir_ provided an initial indication of the reduction of rotational mobility due to crowding, it is still a population average, and a more refined picture was obtained by examining the full distribution of polarization values via polarization histograms and photon scatterplots. In multiple instances, two distinct rotational mobilities were necessary to explain the data, indicating heterogeneity in the environment around the different probe molecules. We consider it likely that most, if not all, of the high Var(*p*)_cir_ values arose from mixed populations (Figure 6 and discussion in **Appendix 1**), one sub-population of which had a relatively low rotational mobility (<10^3^ rad^2^/s). Therefore, despite being fused to a single location in a given protein, mEos3 probes often resided in multiple distinct environments, and variations in Var(*p*)_cir_ values likely arose from both differences in local protein densities as well as differential partitioning between environments.

In all cases that we examined, WGA had a significant effect on the probe’s rotational mobility (Figures 4B and 5B). WGA binds to O-GlcNAc-modified Nups, of which there are at least five in humans (Finlay and Forbes, 1990; Hülsmann et al., 2012). The eight GlcNAc binding sites on the WGA dimer (Schwefel et al., 2010) suggest that it likely inhibited rotational mobility by non-covalently ‘crosslinking’ the FG-network. Considering that WGA affected the rotational mobility of mEos3 probes that were located both in the central pore and on the cytoplasmic and nucleoplasmic sides (Figure 7—figure supplement 1), the most parsimonious conclusion is that WGA binding sites are found throughout the FG-network. This conclusion is also supported by the combined PALM and p-PALM data (Figure 8). However, this conclusion that WGA binding sites are located throughout the FG-network is inconsistent with previous dSTORM microscopy studies that localized WGA to an ~40 nm diameter ring near the scaffold of the central pore (Löschberger et al., 2012). Electron microscopy using WGA-gold revealed a similar picture to the dSTORM study, although more central localizations were also revealed (Akey and Goldfarb, 1989). It is possible that freezing or fixation influences the distribution of WGA-binding sites in these previous studies, which could explain the apparent conflict with the p-PALM data collected here on functional pores. However, there is an alternate interpretation. WGA could significantly increase Var(*p*)_cir_ by binding to a distinct region of the FG-network, and strongly influencing the rotational mobility of the sub-population of probes in the neighborhood of these binding sites. In a highly interconnected network, such as a hydrogel or NTR/FG-polypeptide mixed network, WGA binding in one localized spatial region could influence more distant regions of the FG network via long-range allosteric-type effects. In this way, binding of WGA to one or more discrete spatial locations could influence the rotational motion of a probe throughout the FG-network. This interpretation is consistent with the WGA localization data obtained via dSTORM and electron microscopy (Akey and Goldfarb, 1989; Löschberger et al., 2012). In the case of the mEos3 probe on the N-terminus of Pom121, whose rotational mobility was also significantly reduced by the WGA, it seems unlikely that WGA-binding interactions within the central pore could be transmitted across the NE membrane and influence the rotational mobility of a probe within the perinuclear space. For this reason, we have concluded that the N-terminus of Pom121 is likely to reside within the central pore (Figure 4).

Our results also shed light on the NTR distribution within the NPC. Nearly a hundred molecules of Imp β1 are bound within each NPC during steady-state (Lowe et al., 2015; Paradise et al., 2007; Tokunaga et al., 2008), consistent with the finding that NTRs have high affinities for FG-polypeptides (summarized in [Tetenbaum-Novatt et al., 2012]). A high number of NTRs within the FG-network increases macromolecular crowding, which is expected to influence the structural and functional properties of the FG-network (Kapinos et al., 2014; Lowe et al., 2015; Schleicher et al., 2014; Vovk et al., 2016; Wagner et al., 2015). In particular, the NTR-centric model postulates that NTR/FG-polypeptide effective affinities are higher nearest the NPC scaffold, and significantly weaker in the center of the pore, thus enabling rapid transport only through a narrow channel (~10–20 nm diameter) in the center of the ~50 diameter pore (Kapinos et al., 2014; Schleicher et al., 2014; Wagner et al., 2015). This model therefore predicts significantly higher macromolecular crowding near the scaffold anchor domain of FG-polypeptides. This hypothesis was directly tested via the p-PALM measurements on the mEos3 probe placed at different locations within the Nup98 FG-polypeptide, which support the hypothesis that crowding is indeed higher near the Nup98 anchor domain (Figure 5). Considering mixed populations, the average weighted rotational diffusion constant (*D_r,ave_*) for the probes on mEos3-Nup98 and mEos3-^700mid^Nup98 were ~2800 and ~910 rad^2^/s, respectively (Figure 6—figure supplement 1A C). Similarly, the *D_r,ave_* for the probes on Pom121-mEos3 and mEos3-Pom121 were ~2500 and ~850 rad^2^/s, respectively (Figure 6—figure supplement 2). Therefore, the rotational mobility data for both Pom121 and Nup98 suggest higher macromolecular crowding near the NPC scaffold than at the tips of the FG-polypeptides, consistent with the NTR-centric model. For comparison, in the absence of molecular crowding effects, *D_r_* ≈ 1000 rad^2^/s for the mEos3 probe would correspond to a viscosity of ~10^4^ cP. Notably, the probes on both mEos3-^700mid^Nup98 and mEos3-Pom121 experience multiple environments distinguished by at least two distinct rotational mobilities (Figure 6—figure supplements 1,2). Since the mEos3 probes in both of these constructs are attached near their respective anchor domains, and thus cannot migrate to spatially distinct sites within the FG-network, the local environment must be heterogeneous within an individual NPC, or with respect to different NPCs. Consequently, crowding near the NPC scaffold is somewhat heterogeneous.

High time-resolution super-resolution methods on functional NPCs in unfixed cells will continue to be instrumental in deciphering the complex, amorphous biomaterial that is the FG-network. We demonstrated here that the p-PALM method allows examination of rotational mobility over a range of at least 6 orders of magnitude. This range can be tuned by both acquisition conditions and experimental design, offering significant advantages over bulk measurements of fluorescence anisotropy. The results have allowed us to infer the local binding interactions and molecular crowding within NPCs, and have elucidated multiple aspects of the structural and dynamic complexity of the FG-network. While dynamics are an essential feature of the FG-network, enabling both rapid transport and dynamic maintenance of the permeability barrier, the extent to which newly identified heterogeneities play a role in functional properties of the NPC remains to be explored. While we expect that p-PALM will enable further dissection of the intricacies of the FG-network, it is also well-suited for probing the nanoscale structure of other dense molecular aggregates, such as the poorly understood organization of numerous nucleoplasmic and cytoplasmic membrane-less compartments (‘bodies’), for example, nucleoli, stress granules, and RNA and protein processing bodies (Aumiller et al., 2014; Mitrea and Kriwacki, 2016). These bodies typically contain high concentrations of proteins, and often nucleic acids, and their high densities promote phase separation. These highly crowded environments are difficult to probe because of their rapid dynamics, and often, their small size (<1 µm) (Aumiller et al., 2014; Mitrea and Kriwacki, 2016), and thus, the high time- and super-resolution capability of PALM and the molecular crowding sensitivity of p-PALM provide an important novel tool.

## Materials and methods

### Experimental methods

#### Human cell lines

HeLa cells (authenticated via STR profiling by ATCC) were cultured in Dulbecco’s Modified Eagle Medium (GIBCO, Invitrogen, Carlsbad, CA) supplemented with 4.5 g/L glucose, 862 mg/L Gluta-MAX-I, 15 mg/mL phenol red, 100 U/mL penicillin, 100 μg/mL streptomycin, and 10% (v/v) fetal bovine serum (GIBCO, Invitrogen, Carlsbad, CA). Cells were transfected with expression plasmids using Lipofectamine 2000 according to the manufacturer’s instructions (Invitrogen). For Pom121-mEos3, mEos3-Pom121, mEos3-Nup98, and RanGAP-mEos3, a stable cell line was generated from a single cell clone. In all other cases, cells were split ~24 hr after transient transfections, and were examined ~24 hr after splitting. Cell lines were occasionally tested for mycoplasma contamination.

#### Plasmids

Schematics of the mEos3 fusion proteins produced by the following plasmids can be found in Figure 1—figure supplement 2. Protein expressing inserts of all plasmids were confirmed by DNA sequencing.

*mEos3 –* mEos3.1 was PCR amplified from the mEos3.1 N1 vector (Zhang et al., 2012) (gift of Dr. Tijana Jovanovic-Talisman, City of Hope, Duarte, CA) using forward primer 5'-GTCGCTAGCAGTGCGATTAAGCCAGACATGAAG-3' and reverse primer 5'-CCAGAATTCTTATCGTCTGGCATTGTCAGGCAATCC-3'. The product was digested with Nhe1/EcoR1 (here and elsewhere, restriction sites within primers are underlined) and ligated into pRSETA-mEos2 (gift of Dr. Jie Xiao, Johns Hopkins University, Baltimore, MD) digested with the same enzymes, yielding plasmid pRSETA-mEos3, which produces N-terminally 6xHis-tagged mEos3.

*Pom121-mEos3 –* mEos3.1 was PCR amplified from the mEos3.1 N1 vector using forward primer 5'-TGGCAATTGGGAGGAAGTGCGATTAAGCCAGACATG-3' and reverse primer 5'-CTAACGCGTTTATCGTCTGGCATTGTCAGGCAATCC-3'. The product was digested with Mfe1/Mlu1 and ligated into plasmid peGFP-rPom121 (gift of Dr. Jan Ellenberg, EMBL, Heidelberg) digested with the same enzymes, yielding plasmid peGFP-rPom121-mEos3. Rat Pom121 was PCR amplified from the plasmid eGFP-rPom121 using forward primer 5’-TTTGCTAGCATGTCTCCGGCGGCTGCGGC-3’ and reverse primer 5'-GGGCAATTGTAACTTCTTGCGGGTGTGCTGCCTTCG-3', which mutates the stop codon TTA on Pom121 to TTA. The rPom121 PCR product was digested with Nhe1/Mfe1 and ligated into peGFP-rPom121-mEos3 digested with the same enzymes, yielding plasmid prPom121-mEos3, which produces Pom121-mEos3.

*mEos3-Pom121 –* mEos3.1 was PCR amplified from the mEos3.1 N1 vector using forward primer 5'-GGCGCTAGCATGAGTGCGATTAAGCCAGAC-3' and reverse primer 5'-CGGAGATCTTCGTCTGGCATTGTCAGGCAATC-3', which removes the stop codon on mEos3. The product was digested with Nhe1/Bgl2 and ligated into peGFP-rPom121 digested with the same enzymes, yielding plasmid pmEos3-rPom121(long linker). To remove the long linker between the mEos3 and rPom121 domains, rPom121 was PCR amplified from the plasmid peGFP-rPom121 using forward primer 5’-TTTAGATCTTCTCCGGCGGCTGCGGCGGCTGAC-3’ and reverse primer 5'-CCCACGCGTTTACTTCTTGCGGGTGTGCTGCC-3'. The PCR product was digested with Bgl2/Mlu1 and ligated into pmEos3-rPom121(long linker) digested with the same enzymes, yielding plasmid pmEos3-rPom121, which produces mEos3-Pom121.

*mEos3-Nup153 –* Human Nup153 was PCR amplified from the plasmid peGFP3-Nup153 (gift of Jan Ellenberg, EMBL, Heidelberg) using forward primer 5'-TTAAGATCTGCCTCAGGAGCCGGAGGAGTCG-3' and reverse primer 5'-CGGACGCGTTTATTTCCTGCGTCTAACAGCAGTC-3'. The product was digested with Bgl2/Mlu1 and ligated into plasmid pmEos3-Pom121 digested with the same enzymes, yielding plasmid pmEos3-Nup153, which produces mEos3-Nup153.

*Nup153-mEos3 –* Human Nup153 was PCR amplified from the plasmid peGFP3-Nup153 using forward primer 5'-TATGCTAGCATGGCCTCAGGAGCCGGAGGAGTCG-3' and reverse primer 5'-GGGACGCGTTTTCCTGCGTCTAACAGCAGTCTTTATCTTG-3'. The product was digested with Nhe1/Mlu1 and ligated into plasmid prPom121-mEos3 digested with the same enzymes, yielding plasmid pNup153. Then, mEos3.1 was PCR amplified from the mEos3.1 N1 vector using forward primer 5'- TTTACGCGTGGAGGAAGTGCGATTAAGCCAGACATG-3' and reverse primer 5'- CTAACGCGTTTATCGTCTGGCATTGTCAGGCAATCC-3', which includes a stop codon at the end of the coding sequence for mEos3. Plasmid pNup153 was digested with Mlu1 and the digested product was dephosphorylated by shrimp alkaline phosphatase (rSAP, New England Biolabs, Ipswich, MA). The mEos3.1 PCR product was digested with Mlu1, and ligated into the dephosphorylated pNup153 fragment, yielding plasmid pNup153-mEos3, which produces Nup153-mEos3.

*mEos3-Nup98 –* Human Nup98 was PCR amplified from the plasmid peGFP-Nup98 (gift of Jan Ellenberg, EMBL, Heidelberg) using forward primer 5'-CCGAGATCTTTTAACAAATCATTTGGAACACCCTTTGG-3' and reverse primer 5'-TATACGCGTTCACTGTCCTTTTTTCTCTACCTGAG-3'. The product was digested with Bgl2/Mlu1 and ligated into plasmid pmEos3-Pom121 digested with the same enzymes, yielding plasmid pmEos3-Nup98, which produces mEos3-Nup98. Plasmid pmEos2-Nup98, which produces mEos2-Nup98, was made identically.

*Mutant Versions of mEos3-Nup98 –* Forward primer 5'-TTTAGATCTGCACAAAATAAACCAACTGGCTTTGGC-3' and reverse primer 5'-TATACGCGTTCACTGTCC TTTTTTCTCTACCTGAG-3' were used to obtain a PCR fragment of Nup98 encoding amino acids 110–920, which was digested with Bgl2/Mfe1 and then ligated into plasmid pmEos3-Nup98 digested with the same enzymes, yielding plasmid pmEos3-^110tip^Nup98, which produces mEos3-^110tip^Nup98. Forward primer 5'-CCCGCTAGCATGTTTAACAAATCATTTGGAACACCC-3' and reverse primer 5'-TTTGCTAGCAAAGGCATTGTTTTGGGATGAGAAGAG-3' were used to obtain a PCR fragment of Nup98 encoding amino acids 1–109, which was digested with Nhe1, dephosphorylated as described above, and then ligated into plasmid pmEos3-^110tip^Nup98 digested with the same enzyme, yielding plasmid pmEos3-^110mid^Nup98, which produces mEos3-^110mid^Nup98. The 400tip, 400mid, 500tip, 500mid, 700tip, and 700mid (see Figure 1—figure supplement 2 for nomenclature) mutant expression plasmids were constructed in a similar manner.

*RanGAP-mEos3 –* Mouse RanGAP was PCR amplified from the plasmid pET11d-RanGAP (gift of Jan Ellenberg, EMBL, Heidelberg) using forward primer 5'-TTGGCTAGCATGGCCTCTGAAGACATTGCC-3' and reverse primer 5'-GGGCAATTGGATGTTGTATAGCGTCTGCAGCAG-3'. The product was digested with Nhe1/Mfe1 and ligated into plasmid prPom121-mEos3 digested with the same enzymes, yielding plasmid pRanGAP-mEos3, which produces RanGAP-mEos3.

#### Protein purification

The mEos3, Imp β1 (Kutay et al., 1997b), and Imp β2 (Izaurralde et al., 1997) proteins all contain a 6xHis-tag and were purified by NiNTA and size exclusion chromatography. Plasmids were transformed into *Escherichia coli* BL21(DE3), and protein production (1 L total culture) was induced with 1 mM isopropyl β-D-1-thiogalactopyranoside (IPTG). After overnight growth at 18°C, cells were centrifuged at 5000 *g* for 10 min at 4°C. Pellets (~2 g) were resuspended on ice in 20 mM Tris, 1 M NaCl, 10 mM imidazole, 2 mM β-mercaptoethanol, pH 8.0 with protease inhibitors (1 mM phenylmethylsulfonyl fluoride, 2 µg/mL pepstatin, 2 µg/mL leupeptin, and 20 µg/mL soybean trypsin inhibitor). Resuspended cells were lysed via French press (3X at 1000 psi). The lysate was centrifuged at 50,000 *g* for 15 min. The supernatant was added to a 1 mL Ni-NTA column (Qiagen, Germantown, MD). The Ni^2+^ beads were washed with 40 mL 20 mM Tris, 1 M NaCl, 10 mM imidazole, 0.1% Triton X-100, pH 8.0 with protease inhibitors followed by 40 mL 20 mM Tris, 50 mM NaCl, 20 mM imidazole, pH 8.0 with protease inhibitors. Proteins were eluted with 10 mM Tris, 250 mM imidazole, 50 mM NaCl, pH 8.0. Major protein fractions were combined, concentrated with Microsep 10K or 30K Omega centrifuge filters (Pall Corp., NY), and then further purified by size-exclusion chromatography (Superdex 200; GE Healthcare, Wauwatosa, WI) using 20 mM Hepes, 110 mM KOAc, 5 mM NaOAc, 2 mM MgOAc, 1 mM EGTA, pH 7.3.

#### Cell permeabilization

Cells were permeabilized and prepared for microscopy as previously described (Izaurralde et al., 1997; Lyman et al., 2002; Yang et al., 2004). In short, HeLa cells were grown on coverslips overnight, and an ~20 µL flow chamber was constructed from high-vacuum grease and a top coverslip. Cells were permeabilized by incubation with 40 mg/mL digitonin in import buffer (20 mM Hepes, 110 mM KOAc, 5 mM NaOAc, 2 mM MgOAc, 1 mM EGTA, 1 mM dithiothreitol, pH 7.3) for 2 min. Permeabilized cells were washed twice with import buffer containing 1.5% (w/v) polyvinylpyrrolidone (~360,000 g/mol). When used, WGA (Vector Laboratories, CA), Imp β1, or Imp β2 were incubated with cells for 10 min before imaging.

#### Microscopy

Cells were imaged using a Zeiss 200M inverted microscope, equipped with an alpha plan-apochromat 100X, 1.46 NA oil-immersion objective (Zeiss). A 405 nm laser (100 mW, CUBE, Coherent, Santa Clara, CA) was used to activate the fluorescent protein mEos3 (30–35 W/cm^2^). The green fluorescence of mEos3 was obtained with an ArKr mixed-gas ion laser (2.5 W all lines, Stabilite 2018-RM, Spectra-Physics, Mountain View, CA) at 488 nm and the orange fluorescence of activated mEos3 was obtained with a solid-state laser (150 mW, Excelsior One, Newport, Santa Clara, CA) at 561 nm with an excitation density of 2 kW/cm^2^. Both activation and excitation beams passed through a λ/4 wave plate (ThorLabs, Newton, NJ), generating circularly polarized light. A λ/2 wave plate was used to rotate the angle of linear polarization of the excitation lasers. The sample was illuminated via narrow-field epifluorescence (Yang et al., 2004), that is, a 300 µm pinhole was placed within a specimen-conjugate plane in the activation/excitation beam path, thereby restricting the specimen illumination area to ~7 μm diameter. After passing through a quad-bandpass filter (FF01-446/523/600/677-25, Semrock, Rochester, NY), the fluorescence emission was collected with an EMCCD camera (Evolve 128, Photometrics, Tucson, AZ). Image acquisition was controlled by MetaMorph (Molecular Devices, Sunnyvale, CA).

#### Single molecule localization

Single mEos3 molecules were localized using an algorithm written in Matlab (The MathWorks, Inc., Natick, MA). Fluorescent spots were fit by a symmetric two-dimensional (2D) Gaussian function, whose center was assumed to be the particle's position. Particles in consecutive frames were considered to belong to the same trajectory when they were within a user-defined distance *r*. Considering the NPC size, we set *r* = 200 nm.

#### Image alignment matrix

Coverslip adsorbed 1 µM TetraSpeck microspheres were used for image alignment. Spot centers were determined by 2D Gaussian fitting. The coordinates of *n* spots were summarized as:

$$\begin{array}{ll} X=\left(x_{1}\;x_{2}\cdot \cdot \cdot x_{n}\right)\; & X^{\prime }=\left(x_{1}^{\prime }\;x_{2}^{\prime }\cdot \cdot \cdot x_{n}^{\prime }\right)\\ Y=\left(y_{1}\;y_{2}\cdot \cdot \cdot y_{n}\right)\; & Y^{\prime }=\left(y_{1}^{\prime }\;y_{2}^{\prime }\cdot \cdot \cdot y_{n}^{\prime }\right) \end{array}$$

where *x*_1_, *x*_2_, …, *x*_n_ and *y*_1_, *y*_2_, …, *y*_n_ are the coordinates of the spot centers in the *p*-polarization channel, and the primed values correspond to the coordinates of the spot centers in the *s*-polarization channel. (*X*, *Y*) and (*X*', *Y*') are related as follows:

(1) $$\left[\begin{array}{c} X\\ Y \end{array}\right]=fM_{r}\left[\begin{array}{c} X^{\prime }\\ Y^{\prime } \end{array}\right]+B=f\left[\begin{array}{cc} \cos\Omega & \sin\Omega \\ \sin\Omega & \cos\Omega \end{array}\right]\left[\begin{array}{c} X^{\prime }\\ Y^{\prime } \end{array}\right]+\left[\begin{array}{c} b_{x}\\ b_{y} \end{array}\right]$$

where $M_{r}$ is a rotation, f is zoom factor, and B is a translation. Fit parameters were determined by using the Matlab non-linear least squares fitting function ‘lsqcurvefit’, yielding Ω≈0 and f≈1. Once $M_{r},f,\text{and }B$ were determined, the expected $\left(x_{i},y_{i}\right)$ values were compared with the experimental $\left(x_{i},y_{i}\right)$ values. The standard deviation of the differences, which was considered to be the alignment precision in both x and y, was determined to be ~11 nm for each coordinate.

#### PALM imaging

Samples were illuminated continuously with both 405 nm (activation beam) and 561 nm (measurement beam). Since the number of inactive mEos3 molecules decreased during data acquisition, the intensity of the activation laser was increased from 30 W/cm^2^ to 35 W/cm^2^ over forty 1000-frame movies. As a measure of the rapid photocycling in our experiments, ~80% of the mEos3 molecules in an imaging field were activated and photobleached in ~60 s at an imaging speed of 100 Hz.

#### Localization precision

The localization precision in both the *x* and *y* dimensions can be estimated by (Mortensen et al., 2010):

(2) $$\sigma_{x,y}^{2}=2\left[{\left(\frac{4}{3}\right)}^{2}\frac{s^{2}+a^{2}/12}{N}+\frac{8\pi b^{2}{\left(s^{2}+a^{2}/12\right)}^{2}}{a^{2}N^{2}}\right]$$

where *s* is the standard deviation of the Gaussian fit (~140 nm), *a* is the effective pixel size (240 nm), *b* is the background noise (~3 photons/pixel), and *N* is the total number of photons collected in the spot. Equation 2 yielded an average static localization precision of σ*_x,y_* = ~17 nm for single mEos3 molecules in PALM experiments (*N* ≈ 350 photons).

#### The position of the nuclear envelope (NE) in PALM experiments

Brightfield images of the NE were taken at the beginning of each movie (1000 frames, 10 s duration), and the NE position was determined essentially as described earlier (Yang and Musser, 2006b). The pixel intensities within a row across the NE were fit with a 1D Gaussian function. Peak positions from rows covering the useful area were fit with a cubic function, which was considered to trace the NE.

#### PALM fluorescent spot alignment and overlay

Clusters of three or more fluorescent spots with a maximal distance from their centroid of 200 nm were considered to arise from a single NPC. Inter-cluster distances were >400 nm. A normal to the NE that passed through a cluster centroid was defined as the transport axis of an NPC. Individual NPC transport axes were aligned (translated and rotated) to overlay fluorescent clusters.

#### PALM 2D particle density maps

Origin 7 (OriginLab, Northampton, MA) was used to convert the *x-* and *y*-coordinates of aligned and overlaid fluorescent spots into a 20 × 20 matrix with a bin size of 20 nm. Contours were plotted in intervals of 12.5% of the maximum bin.

Two major sources of error contribute to the broad distributions of PALM localizations along the transport axis (σ > 80 nm: Figure 7). The error in the NE position (σ_NE_) is likely substantial due to NE spatial fluctuations, a non-smooth path of the NE, and heterogeneity near the NE, which affects the accuracy of the bright-field NE localization algorithm. Since previous work has demonstrated resolution of activities occurring on opposite sides of the NPC with particle distribution widths of <50 nm (which includes localization error) (Sun et al., 2013; Sun et al., 2008), we estimate an upper limit of ~45 nm for σ_NE_. The second major source of error is the particle localization error (σ*_x,y_* = ~17 nm; see Localization Precision section). While other sources of error exist (Musser and Grünwald, 2016), σ_NE_ and σ*_x,y_* dominate and yet are insufficient to explain the broad particle distributions along the transport axis. The most likely additional major contributors to the broad PALM distribution maps are multiple anchoring sites for the labeled proteins and motion of the FG-polypeptides themselves. Higher resolution data is required for more precise conclusions on FG-polypeptide translational mobility and more refined maps of FG-polypeptide distributions and anchoring sites.

#### Polarization PALM (p-PALM)

For p-PALM, the emission light was separated with a 50% polarizing beam splitter cube (PBS201, ThorLabs, Newton, NJ) mounted in an Optosplit III beamsplitter (Cairn Research, Kent, UK). The two polarization components were imaged simultaneously on the two halves of the EMCCD camera. A system-dependent factor, g, corrects for differences in photon collection efficiency by the *p*- and *s*-detection channels, and must be empirically determined to calculate the polarization according to:

(3) $$p=\frac{\left(gI_{p}-I_{s}\right)}{\left(gI_{p}+I_{s}\right)}$$

where $I_{p}$ and $I_{s}$ are the intensities measured in the two detection channels. To estimate g, we measured the intensities of mEos3 molecules in 92% glycerol, which rapidly rotate on the data collection timescale (Figure 3—figure supplement 1), and therefore, $<p{>}_{\text{cir}}$ is assumed to be 0. Under these conditions $g=<I_{s}/I_{p}>=0.92$.

#### Polarization histograms

Except for the data collected in 92% glycerol (see Figure 3—figure supplement 1), only molecules that lasted for three or more frames were used for polarization analysis. Polarization values were only calculated for the second frame of each trajectory. This approach was designed to only include intensities that reflected photon emission over the entire frame (i.e., ensuring that photoactivation or photobleaching did not occur during data collection) and to weight each trajectory/molecule equally. Both of these constraints are essential to accurately estimate rotational mobility. Note that pooling p-PALM images would increase the integration time and the number of photons collected, both of which influence the measurement (Figure 2—figure supplements 2A,5). Pixel-dependent background intensities were determined by averaging a 1000-frame movie collected at the end of the experiment after complete photobleaching of mEos3.

### Rotational random walk simulations

#### Overview of the problem

For a single fluorophore molecule, the photons collected in a single image correspond to hundreds of excitation and emission cycles, during and between each of which the probe might rotate. To decipher how polarization measurements are affected by the various conditions that could occur in p-PALM experiments, we estimated the photons collected (intensities measured) in the *p*- and *s*-polarization channels using rotational random walk simulations. Rotational random walk trajectories were simulated using Microsoft Excel with the RiskAMP Monte Carlo Simulation Engine (https://www.riskamp.com).

A freely diffusing spherical particle has identical rotational diffusion constants for the molecule's three principle rotational axes. Such a particle reasonably approximates most globular proteins (Loman et al., 2010). However, for an mEos3 molecule tethered to an FG-polypeptide, restrictions on rotational mobility due to the tether point can be expected. mEos3 has a β-barrel structure (Zhang et al., 2012). It was tethered to FG-polypeptides via its N- or C-terminus (or both), each of which are at the bottom of this β-barrel. For simplicity, we assumed that the tether point was on the rotational *z*-axis, and that this was coincident with the β-barrel axis. Restricted movement of the tether point thus results in *D_z_* > *D_x_* ≈ *D_y_* (Appendix 1—figure 1). We assumed that the excitation and emission transition dipole moments of mEos3 are parallel, which is the case for GFP and many fluorophores (Ha et al., 1999; Inoué et al., 2002), and therefore, we usually more simply refer to these as the transition dipole. The transition dipole was assumed to be in the *yz*-plane of the molecule, and, since the transition dipole of GFP is ~60° from the β-barrel axis (Inoué et al., 2002), we assumed this to be approximately true for mEos3 as well (Zhang et al., 2012). Appendix 1—figure 2 demonstrates the effects of varying the angle (γ) between the transition dipole and the rotational *z*-axis.

#### Summary of rotational random walk simulation algorithm

The initial output of the rotational random walk simulations was the number of photons collected in each of the two polarization channels. The approach is briefly summarized here – details follow in subsequent sections. The dipole's initial orientation was chosen randomly. For each time step, the dipole was rotated around its three principle rotational axes via an angular step randomly chosen from a normal distribution defined by its rotational diffusion constant. Three decisions were then used to determine if a photon was collected, and if so, to which detection channel it went: (1) the excitation of the probe was stochastically determined based on its excitation probability, given the illumination ellipticity and the orientation of the dipole; (2) the probability of photon collection was determined based on the dipole’s orientation and the solid angle subtended by the objective NA; and (3) if a photon was collected, it was partitioned into either the *p*- or *s*-channel depending on probabilities determined by the dipole's orientation. Most rotational random walk steps did not result in the collection of a photon. For circular excitation, the number of steps (*N_s_*) for most simulations was 1400, leading to an average of ~352 photons collected under high *D_r_* conditions (Figure 2—figure supplement 5), which matches well with the average of ~300–400 total photons collected in the two polarization channels in p-PALM experiments (Supplementary file 1). Under these simulation conditions, an average of 341 total photons are expected based on the average excitation efficiency (2/3) and the photon collection efficiency of the objective (~36.5%). For linear excitation, *N_s_* = 2800 was used to compensate for the lower average excitation efficiency (1/3). Thresholding (see later) is responsible for the higher than expected average total photons collected, particularly under low *D_r_* conditions (Figure 2—figure supplement 5).

#### Coordinate systems and rotation transformations

For the laboratory frame, we assumed a spherical coordinate system where φ describes the angle from the *z*-axis (optical axis), and θ describes rotation around the *z*-axis from the *x*-axis. Random walk steps consisted of three angular sub-steps, one each around the molecule's three principal rotation axes, which were randomly oriented at the beginning of each simulation. The molecule's coordinate system was continuously updated and output to the laboratory frame using vector cross product multiplication to determine perpendicular unit vectors. Rotations were calculated by quaternion multiplication as follows. A rotation of the unit dipole vector ***a*** = *a_x_***i** + *a***_y_j **+ *a_z_***k** by an angle ϕ around the unit rotation axis vector ***u*** = *u_x_***i** + u_y_**j**+ *u_z_***k** was calculated as:

(4) $$a^{\prime }=q^{-1}aq$$

where ***a'*** is the rotated vector and the quaternion (***q***) and its conjugate (***q^−1^***) were given by,

(5) $$q=\cos\frac{\phi }{2}+\left(u_{x}i+u_{y}j+u_{z}k\right)\sin\frac{\phi }{2}$$

(6) $$q^{-1}=\cos\frac{\phi }{2}-\left(u_{x}i+u_{y}j+u_{z}k\right)\sin\frac{\phi }{2}$$

Angular sub-step sizes were randomly selected from a normal distribution centered around the starting position with a variance of ϕ^2^ = 2*D*τ (with *D* = *D_x_*, *D_y_*, or *D_z_*, as required for the rotational axis), where τ is the duration of each angular step, or ϕ^2^ = 2*D*τ_m_, where τ_m_ is time between excitation and emission of the molecule. The τ_m_ at each step was randomly chosen from an exponential decay defined by the fluorescence lifetime (τ_F_) of mEos3, which was assumed to be 3.5 ns (Adam et al., 2011). Each rotational random walk cycle included an excitation step, rotation during the fluorescence lifetime (three independent sub-steps defined by *D_x_*, *D_y_*, and *D_z_*), and rotation after the photon was emitted (three independent sub-steps). Since *t* is the image integration time, τ = *t*/*N_s_*.

#### Excitation probabilities and emission intensities

Excitation probability is proportional to the square of the magnitude of the electric field along the direction of the transition dipole (Forkey et al., 2000). Therefore, since we used a narrow-field excitation approach (i.e. with a very low NA for excitation), the excitation probability at each time step was set to (sin^2^φ)(cos^2^θ + (1/ε)sin^2^θ), where the ellipticity is defined by ε = *E_x_*/*E_y_*, and where *E_x_* and *E_y_* correspond to the average magnitudes of the electric fields along the *x*- and *y*-laboratory axes. Typical values used in the simulations were ε = 1 (circularly polarized light) and ε = 100 (linearly polarized light).

For a fixed unit dipole with laboratory frame components (*x*, *y*, *z*), the emission intensities collected in the *p*- and *s*-channels are:

(7) $$i_{p}=i_{tot}\left(K_{1}x^{2}+K_{2}y^{2}+K_{3}z^{2}\right)$$

(8) $$i_{s}=i_{tot}\left(K_{2}x^{2}+K_{1}y^{2}+K_{3}z^{2}\right)$$

where

(9) $$K_{1}=\frac{3}{32}\left(5-3\cos\left(\theta_{obj}\right)-\cos^{2}\left(\theta_{obj}\right)-\cos^{3}\left(\theta_{obj}\right)\right)$$

(10) $$K_{3}=\frac{1}{32}\left(1-3\cos\left(\theta_{obj}\right)+3\cos^{3}\left(\theta_{obj}\right)\cos^{3}\left(\theta_{obj}\right)\right)$$

(11) $$K_{3}=\frac{1}{8}\left(2-3\cos\left(\theta_{obj}\right)+\cos^{3}\left(\theta_{obj}\right)\right)$$

are constants determined from Axelrod's expressions (Axelrod, 1979) normalized such that *i_p_* + *i_s_* = *i_tot_* for θ*_obj_* = 180° (all light collected) (Ha et al., 1999). Assuming an angular semiaperture of θ*_obj_* = 74.1° estimated from an immersion oil index of refraction of *n* = 1.518 and NA = *n* sin(θ*_obj_*)=1.46, these constants were calculated as *K_1_* = 0.38, *K_2_* = 0.012, and *K_3_* = 0.15 (but see Appendix 1—figure 4). For values of θ*_obj_* < 180°, *i_p_* + *i_s_* + *i_e _*= *i_tot_* where *i_e_* is the intensity of photons that escaped detection. Thus, the probability that an emitted photon escapes detection is:

(12) $$P_{e}=\frac{i_{e}}{i_{tot}}=1-\frac{i_{p}}{i_{tot}}-\frac{i_{s}}{i_{tot}}$$

and the probability that the photon is collected is 1 – *P_e_*. If a photon is collected, the probability that the photon is partitioned into either the *p*- or *s*-channel is:

(13) $$P_{p}=\frac{i_{p}}{i_{p}+i_{s}}$$

(14) $$P_{s}=\frac{i_{s}}{i_{p}+i_{s}}=1-P_{p}$$

Note that *i_tot_* ends up being simply a scaling factor that disappears upon calculation of these probabilities. Note also that the high NA implies that photons can be collected in either the *p*- or *s*-channels from molecules oriented with a *z*-axis component, which reduces the number of measured polarization values near the ±1 limits (Figure 2).

Equation 12 was additionally verified using simulations in which it was determined if the propagation direction of the emitted photon allowed it to be captured based on θ*_obj_*. The photon’s propagation direction was randomly chosen assuming that the propagation direction is proportional to sin^2^φ*_k_* (Forkey et al., 2000), where φ*_k_* is the angle between the transition dipole and the propagation direction. Identical results were obtained.

#### Broadening intensity distributions and addition of background noise

In order that the simulation results more accurately reflected experimental intensity distributions and noisy single molecule data, emission intensities based on photon counts were broadened and background noise was added. Whereas histograms of total photon intensities from the simulations were Gaussian under high rotational mobility conditions, experimental intensity histograms were approximately three-fold wider and were better described by log-normal distributions (e.g. Figure 6E), consistent with previous observations (Mutch et al., 2007). The broader experimental intensity distributions likely arise from a variety of factors, including the amplification noise of the EMCCD camera (Chao et al., 2013), differential focusing, irregularities within different light paths (permeabilized cells provide complex scattering and refractive index changes), as well as dirt and aberrations/variations in the optics and pixel quantum efficiencies (Mutch et al., 2007). Conformational differences of the probe that affect photon output are also possible. The simulated intensity distributions were therefore broadened by random selection of intensities from log-normal distributions with scale parameter μ* = the total photons collected at each time step and shape parameter σ* = 0.24. The value of the shape parameter was guided by fits to experimental intensity distributions and agreement of the final simulation results to experiment. The value of σ* has essentially no effect on <*p*> and Var(*p*) (Figure 2—figure supplement 4). Log-normal distributions have an advantage over normal distributions for the purpose described here, particularly for lower intensities, since negative values cannot occur. Background noise was added to the *p*- and *s*-intensities recalculated from the new total intensities, partitioned according to the original photon numbers. Background noise (in photons) was normally distributed with σ_p_ = 15 and σ_s_ = 15 for circular excitation, and σ_p_ = 22 and σ_s_ = 15 for linear excitation, which are the average noise levels in p-PALM experiments over the 5 × 5 pixel regions of interest used to obtain fluorescence intensities. This approach yielded intensity distributions, *ps*-photon scatterplots, and polarization histograms that resembled the experimental data (Figure 6).

#### Thresholding

A threshold was implemented to distinguish single molecule signals from background noise in experimental measurements. For rapidly rotating particles, distinguishing single molecule signals from background was fairly straightforward. However, slowly rotating particles have strongly preferred transition dipole orientations, and therefore, intensity histograms were broader for such molecules, as expected, since excitation probabilities and emission intensities depend on dipole orientation. In particular, dipoles with a strong *z*-component yielded fewer emission photons primarily due to poor excitation efficiency, and therefore, molecules with such preferred orientations were not reliably detected in our experiments. Our criteria for spot selection was ≥100 photons in either the *p*- or *s*-detection channel.

To ensure more accurate modeling of the experimental data, a 100 photon threshold was also implemented for the simulation results. When using linear excitation, this threshold eliminates ~50% of the *p* values < 0 when *D_r_* is low (Regime V; compare Figure 2B and Figure 2—figure supplement 1B). Consequently, under these conditions, a 100 photon threshold increases <*p*>_lin_ from ~0 to ~0.24 (Figure 2—figure supplement 4A). This is a major change, and indicates an extreme sensitivity to imaging and analysis parameters. In contrast, Var(*p*)_lin_ and Var(*p*)_cir_ values are less sensitive to this threshold for all values of *D_r_* (Figure 2—figure supplement 4B C). Notably, however, Var(*p*)_cir_ has a wider dynamic range than Var(*p*)_lin_ at low *D_r_* values, indicating greater sensitivity to changes in rotational mobility. For circular excitation and small *D_r_* values, the threshold eliminates ~20% of the simulation values, which predominantly increases the low *D_r_* asymptote in Var(*p*)_cir_ plots. Under these conditions, the thresholding selects against particles oriented with a strong *z*-component, for which *x*- and *y*-intensities are similar and low, leading to the selective elimination of *p* values near zero (see Figure 2D) and an increase in Var(*p*)_cir_. Background noise also contributes to increasing this asymptotic value, although noise predominantly influences the high *D_r_* asymptote (Figure 2—figure supplement 4C). In contrast, the 100 photon threshold does not affect the high *D_r_* asymptote since at high *D_r_* values all measurements are above the threshold. Notably, thresholding has little influence in most of the region where Var(*p*)_cir_ is most sensitive to *D_r_* (Figure 2—figure supplement 4C). These simulations indicate that measuring <*p*>_lin_ is best for rotational diffusion regimes I-III and measuring Var(*p*)_cir_ is best for regimes III-V.

#### Single molecule vs. bulk results

Single molecule and bulk fluorescence data yield fundamentally different information under conditions of low rotational mobility. This is directly observed in Figure 2A, where the single molecule and ensemble polarization results are compared for linear excitation. The emission intensities collected in the *p*- and *s*-polarization channels, *I_p_* and *I_s_*, are mixtures of the intensities emitted along the *x*-, *y*-, and *z*-axes of the laboratory frame, *I_x_*, *I_y_*, and *I_z_*, respectively:

(15) $$I_{p}=K_{1}I_{x}+K_{2}I_{y}+K_{3}I_{z}$$

(16) $$I_{s}=K_{2}I_{x}+K_{1}I_{y}+K_{3}I_{z}$$

where *K_1_*, *K_2_*, and *K_3_* are defined in Equations 9-11. For *x*-polarized linear excitation, *I_y_* = *I_z_*, and therefore:

(17) $$I_{x}=I_{∥}=\frac{\left(K_{1}+K_{3}\right)I_{p}-\left(K_{2}+K_{3}\right)I_{s}}{\left(K_{1}-K_{2}\right)\left(K_{1}+K_{2}+K_{3}\right)}$$

(18) $$I_{y}=I_{z}=I_{⊥}=\frac{K_{1}I_{s}-K_{2}I_{p}}{\left(K_{1}-K_{2}\right)\left(K_{1}+K_{2}+K_{3}\right)}$$

Thus, the corrected polarization (*p*_c_) and anisotropy (*r*_c_) values calculated for individual molecules in p-PALM images are given by:

(19) $$p_{c}=\frac{I_{∥}-I_{⊥}}{I_{∥}+I_{⊥}}=\frac{\left(K_{1}+K_{2}+K_{3}\right)\left(I_{p}-I_{s}\right)}{\left(K_{1}-K_{2}+K_{3}\right)I_{p}+\left(K_{1}-K_{2}-K_{3}\right)I_{s}}$$

(20) $$r_{c}=\frac{I_{∥}-I_{⊥}}{I_{∥}+2I_{⊥}}=\frac{\left(K_{1}+K_{2}+K_{3}\right)\left(I_{p}-I_{s}\right)}{\left(K_{1}-2K_{2}+K_{3}\right)I_{p}+\left(2K_{1}-K_{2}-K_{3}\right)I_{s}}$$

Using these expressions, the corrected values for the mean polarization (<*p*>*_lin,c_*) and the mean anisotropy (<*r*>*_lin,c_*) measured in p-PALM experiments are given by:

(21) $$<p{>}_{lin,c}=\frac{1}{N}\sum_{k=1}^{N}p_{c}$$

(22) $$<r{>}_{lin,c}=\frac{1}{N}\sum_{k=1}^{N}r_{c}$$

and are plotted in Figure 2A and Figure 2—figure supplement 3, respectively. The bulk polarization (*p*_bulk_) and anisotropy (*r*_bulk_) were estimated by summing the intensities from 10,000 different molecules with random initial orientations:

(23) $$p_{bulk}=\frac{\sum_{k}I_{∥}-\sum_{k}I_{⊥}}{\sum_{k}I_{∥}+\sum_{k}I_{⊥}}$$

(24) $$r_{bulk}=\frac{\sum_{k}I_{∥}-\sum_{k}I_{⊥}}{\sum_{k}I_{∥}+2\sum_{k}I_{⊥}}$$

These expressions yield low *D_r_* limiting values of 0.5 and 0.4, respectively (Figure 2A and Figure 2—figure supplement 3), as expected (Lakowicz, 2006). The bulk and p-PALM results differ for two reasons (discussed more fully in the text): (1) the microscope objective mixes polarizations (Equations 15 and 16); and (2) the values calculated from single molecule data weights each molecule identically (i.e. the polarization/anisotropy of each molecule counts the same no matter how many photons are emitted), whereas the values corresponding to bulk conditions weights the contribution of each molecule to the measured polarization/anisotropy (a single measurement) depending on the number of photons emitted. The bulk polarization and anisotropy under circular excitation are always 0.

Under single molecule conditions, the variance in the measured polarization values, Var(*p*), provides a measure of the width of polarization histograms (see Figure 2). The variance was calculated as Var(*p*) = <*p*^2^> – (<*p*>)^2^, where <*p*^2^> is the average square of the measured polarization values. Theoretically, using circular excitation, Var(*p*) = <*p*^2^> since <p> = 0 for all *D_r_* values. Consequently, since in most cases we obtained <*p*>_cir_ ≈ 0 (see Supplementary file 1), we assumed that Var(*p*)_cir_ was equivalent to the experimentally determined <*p*^2^>_cir_. As defined here, Var(*p*) does not exist under bulk conditions since the information from all individual molecules is integrated by the measurement into a single value. This fundamentally explains why circular polarized excitation provides rotational mobility information under slow rotation conditions in a single molecule p-PALM experiment, whereas no information is obtained in a corresponding bulk experiment.

#### Inferring *D_r_* from Var(*p*)_cir_

A general analytical solution for the Var(*p*)_cir_ dependence on the rotational diffusion constants is a complex problem. A few special cases are described in the Appendix. Guided by these results, it became clear that Pade-like approximations could be used for curve fitting, which enables extraction of rotational diffusion constants from measurements of Var(*p*)_cir_. Here, we summarize our findings from a range of simulations where *D_x_*, *D_y_*, *D_z_*, *t*, γ, background noise and threshold values were varied.

The three principle rotational axes, *x*, *y*, and *z*, of a particle are characterized by the rotational diffusion constants *D_x_*, *D_y_*, and *D_z_*, respectively, with an average rotational diffusion constant given by *D_r_* = (*D_x_* + *D_y_* + *D_z_*)/3. For a freely diffusing sphere, *D_x_* = *D_y_* = *D_z_ *= *D_r_*. For a sphere whose rotational motion is restricted via a tether point on its rotational *z*-axis, *D_x_* = *D_y_*, the average perpendicular rotational diffusion constant is *D*_⊥_ = *D_xy_* = (*D_x_* + *D_y_*)/2, and *D_r_* = (2*D*_⊥_ + *D_z_*)/3. *D_xz_* and *D_yz_* are similarly defined. For a particle that is tethered via multiple constraints, is highly geometrically asymmetrical, or has its rotational diffusion differentially constrained by other means, *D_x_* ≠ D*_y_* ≠ D_z_.

For a particle behaving similar to a freely diffusing sphere (i.e., *D_x_* ≈ *D_y_* ≈ *D_z_*), we find that (e.g., see Appendix 1—figure 3):

(25) $$\text{Var}{\left(p\right)}_{cir}=\frac{\left(1+a_{1}D_{r}t\right)}{\beta +a_{2}D_{r}t+a_{3}{\left(D_{r}t\right)}^{2}}+\alpha$$

where α, β, and *a_1-3_* are fit parameters that depend on γ, noise, the average number of photons collected, and the threshold. The value of α is determined by the high mobility limit (*D_r_t* → ∞; lower asymptote), and is the same regardless of the relationship between the rotational diffusion constants. The value of β (with a small contribution from α) determines the low mobility limit (*D_r_t* → 0; upper asymptote). In most cases, *a_1_* ≈ 1 works well. Using the quadratic equation, *D_r_* is obtained from the positive root of Equation 25:

(26) $$\;D_{r}=\frac{1}{2a_{3}t}\left[\frac{a_{1}}{\text{Var}{\left(p\right)}_{cir}-\;\alpha }-a_{2}+\sqrt{\left(a_{2}^{2}-4a_{3}\beta \right)+\frac{4a_{3}-2a_{1}a_{2}}{\text{Var}{\left(p\right)}_{cir}-\;\alpha }+{\left(\frac{a_{1}}{\text{Var}{\left(p\right)}_{cir}-\;\alpha }\right)}^{2}}\right]$$

which allows calculation of *D_r_* from a Var(*p*)_cir_ measurement.

In more complex situations (e.g., *D_x_* ≈ *D_y_* << *D_z_* or *D_x_* << *D_y_* << *D_z_*), we find that Var(*p*)_cir_ is well-approximated by:

(27) $$\text{Var}{\left(p\right)}_{cir}\approx \left(1+a_{1}D_{r}t\right)\left[\frac{1}{\beta_{1}+a_{2}D_{r}t+a_{3}{\left(D_{r}t\right)}^{2}}+\frac{1}{\beta_{2}+a_{4}D_{r}t+a_{5}{\left(D_{r}t\right)}^{2}}\right]+\alpha$$

The β_2_ value determines an intermediate plateau (e.g., see Appendix 1—figure 3). Parameters from simpler situations can be used as a guide to fit data from more complex situations and often work well, but the parameter values are not strictly the same. We emphasize that Equations 25 and 27 are empirical solutions used to obtain smooth fits of simulation data. As far as we know, the constants *a_1-5_* do not have a physical interpretation.

#### Error in Var(*p*)_cir_ measurements

The simulation results were used to estimate the number of experimental data points required to obtain a reasonable estimate of Var(*p*) and the error in the measurement. Datasets of 500–2000 measurements were randomly divided into 4 equivalently sized datasets, and means and standard deviations (SDs) were calculated. As shown in Figure 2—figure supplement 6, an experimental Var(*p*)_cir_ value obtained from ~2000 (4 × 500) measurements differs from the ‘true’ value by an average of ~2–3%. The ‘true’ value was included in the range defined by the mean ±SD approximately 85% of the time, and thus this range defines the 85% confidence interval. Errors (SDs) for all experimental <*p*> and Var(*p*) values were calculated in this manner (from 4 equivalently sized datasets), and are summarized in Supplementary file 1.

## Figures and Tables

**Appendix 1—figure 1.**
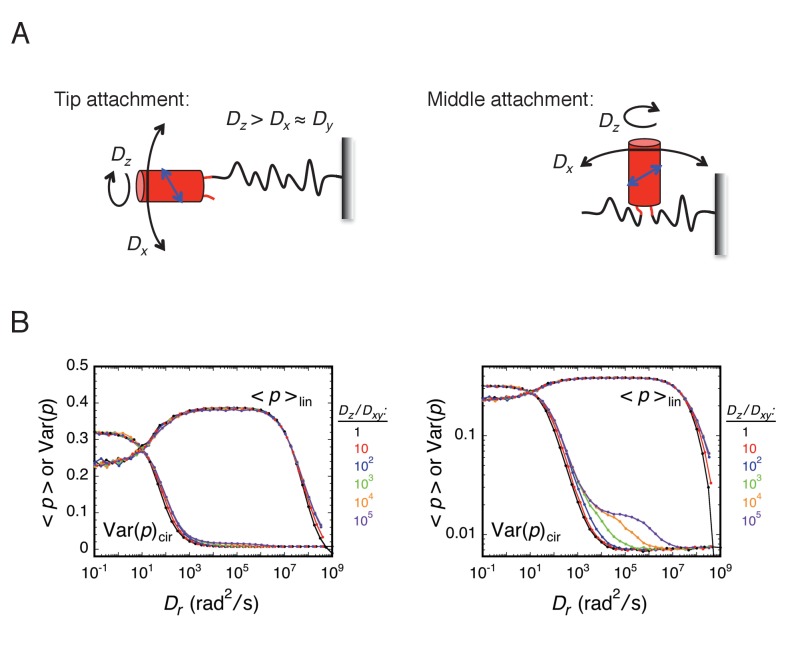
Effect of rotational anisotropy, expressed through the *D_z_*/*D_xy_* ratio, on <*p*>_lin_ and Var(*p*)_cir_. (**A**) Rotational modes for an mEos3 protein attached to the tip or middle of an FG-polypeptide. For 'tip' attachment, free rotation of mEos3 (*red*) is expected around the bonds in the tether (polypeptide link). 'Middle' attachment could potentially inhibit *z*-axis rotation because this would require the linked FG-polypeptides to twist in a coordinated fashion. Rotational motion about the three rotational axes could potentially be differentially influenced by local crowding and the motion of the FG-polypeptide domain(s) to which the probe is attached. The transition dipole (*blue*) is assumed to be within the *yz*-plane at an angle of ~60° to the rotational *z*-axis (assumed to be approximately equivalent to the β-barrel axis). (**B**) Effect of the *D_z_*/*D_xy_* ratio. Rotational random walk simulations were used to estimate <*p*>_lin_ and Var(*p*)_cir_ for different *D_z_*/*D_xy_* ratios, assuming *t* = 10 ms. Under these conditions, the effect of the *D_z_*/*D_xy_* ratio is relatively minor (but see Appendix 1—figures 2 and 3). The panel on the *right* shows the same information as the one on the *left*, except that the ordinate is log-scaled to more easily reveal the biphasic relationship of Var(*p*)_cir_ with *D_r_* at high *D_z_*/*D_xy_* ratios.

**Appendix 1—figure 2.**
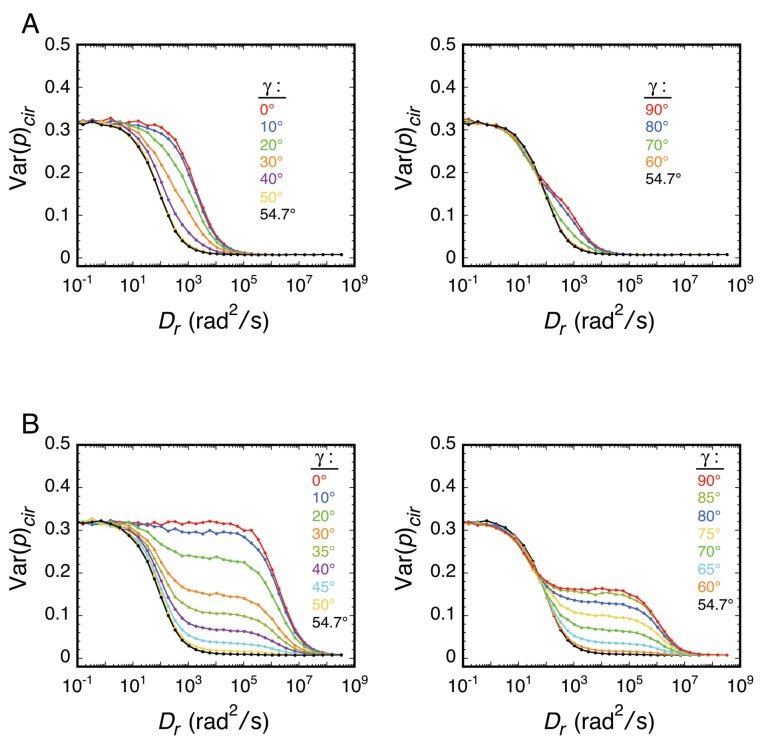
Effect of γ on the relationship between Var(*p*)_cir_ and *D_r_*. Rotational random walk simulations were used to estimate Var(*p*)_cir_ for different γ values (*t* = 10 ms). (**A**) *D_z_*/*D_xy_* = 100. (B) *D_z_*/*D_xy_* = 100,000.

**Appendix 1—figure 3.**
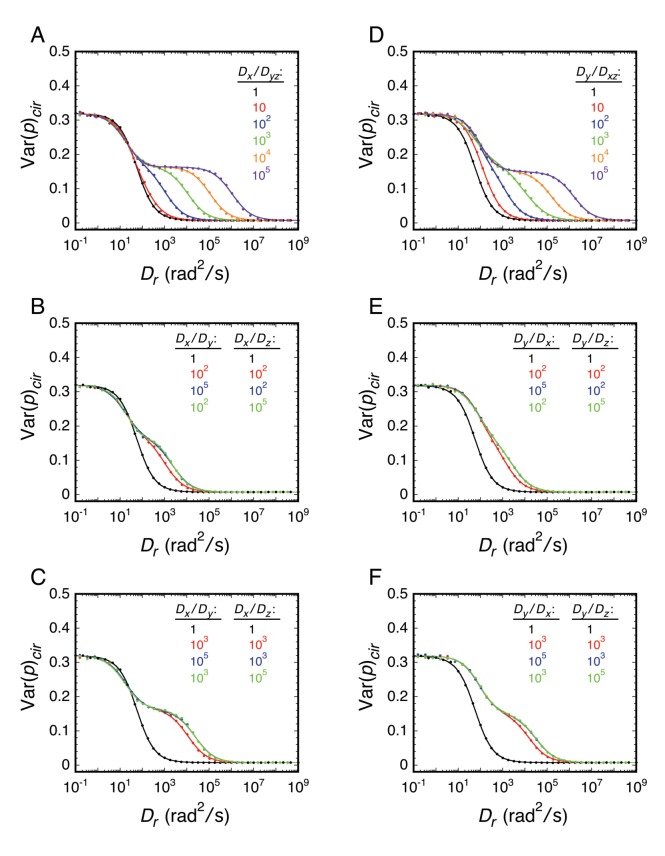
Effect of rotational diffusion constant anisotropy on the relationship between Var(*p*)_cir_ and *D_r_*. For all results in this figure, γ = 60° and the transition dipole was in the *yz*-plane. (**A**) Effect of the *D_x_*/*D_yz_* ratio, where *D_yz_* = *D_y_ = D_z_*. (**B and C**) Effect of *D_x_* > *D_y_*, *D_x_* > *D_z_*, and *D_y_* ≠ *D_z_*. Since the transition dipole was in the *yz*-plane, *D_x_* = 100*D_y_ *= 100*D_z_* is equivalent to γ = 90° in Appendix 1—figure 2A. (**D**) Effect of the *D_y_*/*D_xz_* ratio, where *D_xz_* = *D_x_ = D_z_*. (**E and F**) Effect of *D_y_* > *D_x_*, *D_y_* > *D_z_*, and *D_x_* ≠ *D_z_*. Since the transition dipole was in the *yz*-plane 60° from the *z*-axis, *D_y_* = 100*D_x_ *= 100*D_z_* is equivalent to γ = 30° in Appendix 1—figure 2A. In all cases, *D_r_* = (*D_x_* + *D_y_* + *D_z_*)/3 and *t* = 10 ms. Results were fit using Equations 25 and 27. In (**B**), (**C**), (**E**), and (**F**), the *green* and *blue* curves overlap. The large effects of diffusion constant anisotropy shown here contrast with the small effects shown in Appendix 1—figure 1. The latter results from the assumption that γ = 60°, which is near the magic angle of 54.7° (see Appendix 1—figure 2).

**Appendix 1—figure 4.**
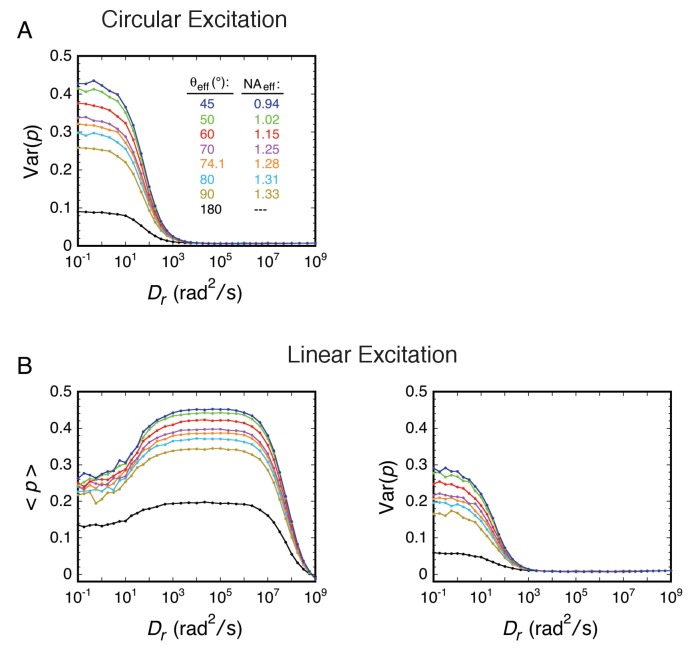
Effects of the numerical aperture. (**A**) Effect of the NA on Var(*p*)_cir_. (**B**) Effect of the NA on <*p*>_lin_ and Var(*p*)_lin_. For most of the rotational random walk simulations reported in this paper, we have assumed an angular semi-aperture of θ_obj_ = 74.1°, where θ_obj_ is the half-angle of the cone of light recovered by the microscope objective. This θ_obj_ was calculated in the normal way (Ha et al., 1999), assuming an immersion oil refractive index of *n* = 1.518 and NA = *n* sin(θ_obj_) where NA = 1.46 is the value inscribed on the objective. This approach assumes that the sample is embedded in a medium with refractive index equivalent to that of immersion oil. The NA_eff_ for an aqueous sample depends on a variety of factors, including distance from the surface, spherical aberrations, and scattering. Our p-PALM data are most consistent with θ_eff_ ≈ 50–60° (see text for details). In the figure, NA_eff_ = 1.33 sin(θ_eff_), where *n* = 1.33 is the refractive index for water. θ_eff_ = 180° describes a perfect objective (all light collected), and is compared with an analytical calculation in Appendix 2—figure 3 (see **Appendix 2**). θ_eff_ = 90° represents the theoretical maximum with current objective designs. The color key is the same for all graphs.

**Appendix 1—figure 5.**
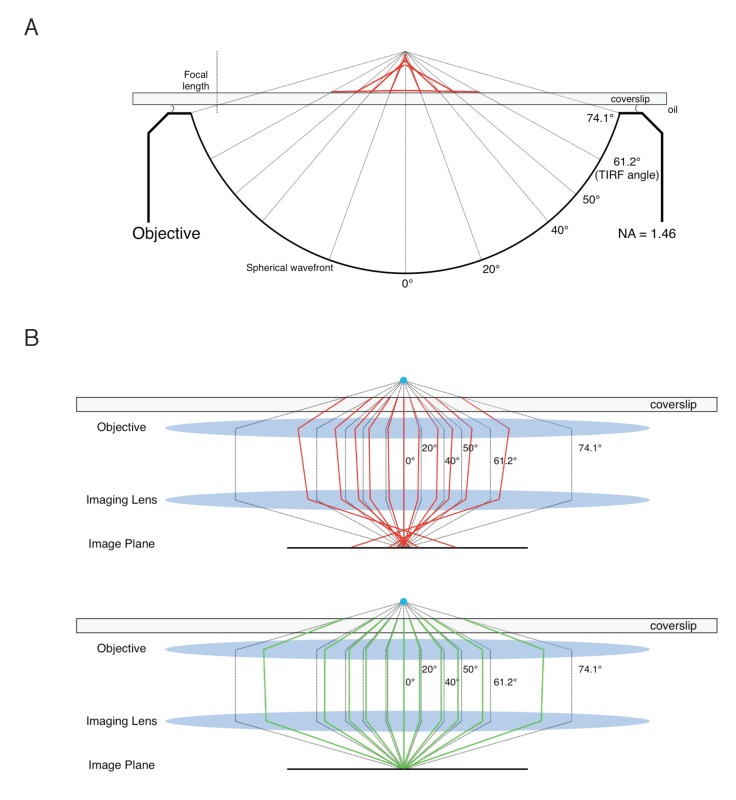
Effect of spherical aberration on NA_eff_. Refractive index mismatch causes spherical aberration. (**A**) Effect of refractive index mismatch on the point spread function (PSF). When an oil-immersion objective is used to illuminate a sample in identical refractive index medium (*n* = 1.518), all rays converge upon a single focal point (*dashed lines*). When illuminating an aqueous sample, the rays are refracted (*red*), and the PSF is elongated along the optical axis. (**B**) Effect of refractive index mismatch on image quality. (*top*) Imaging an aqueous sample (*n* = 1.33) with an oil-immersion objective leads to a poorly focused image with loss of photons emitted at high angles to the optical axis, thus reducing the NA_eff_ (*refracted rays in red*). (*bottom*) Imaging a sample in glycerol (*n* = 1.47) with the same objective leads to a more tightly focused image (*refracted rays in green*). If the objective is corrected for spherical aberrations introduced by aqueous samples, the opposite will be true – the PSF will be smaller for aqueous samples and a sample in glycerol will be poorly focused and have a low NA_eff_.

**Appendix 1—figure 6.**
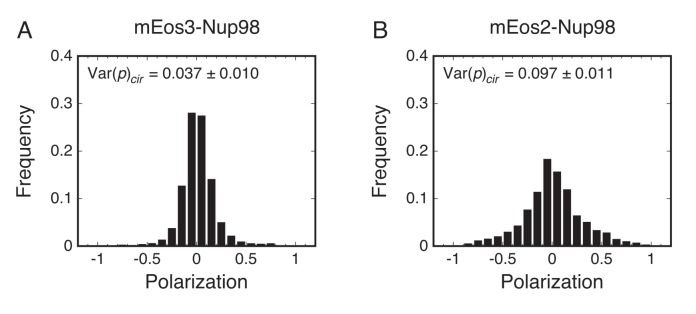
Reduced rotational mobility of the mEos2 probe. p-PALM measurements were made on mEos3 (**A**) and mEos2 (**B**) attached to the N-terminus of Nup98. The rotational mobility of mEos2 was significantly lower, likely due to the tendency of mEos2 to dimerize (Hoi et al., 2010; McKinney et al., 2009).

**Appendix 2—figure 1.**
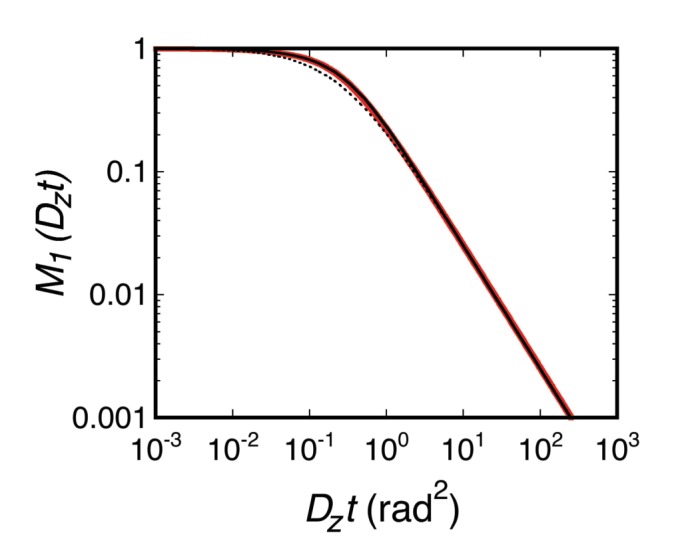
*M_1_*(*D_z_t*). Equation 48 was evaluated by numerical integration (red). The dashed black curve is Equation 49, and the solid black curve is Equation 50.

**Appendix 2—figure 2.**
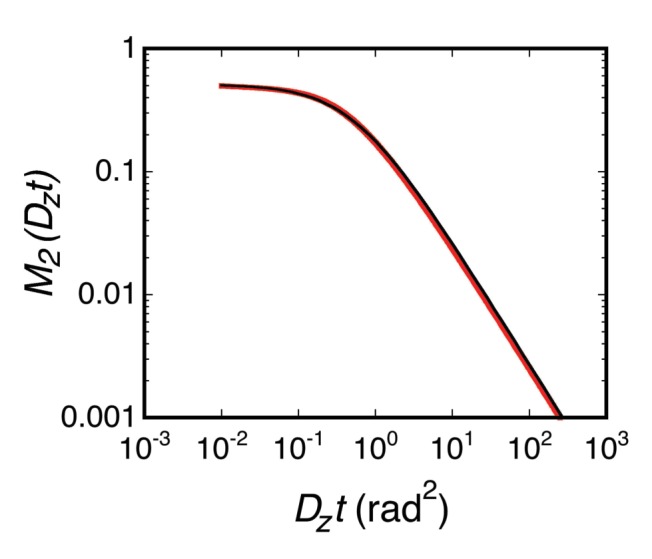
*M_2_*(*D_z_t*). Equation 55 was evaluated by numerical integration (red). The solid black curve is the best fit, given in Equation 56 as *M_2_*(*D_z_t*) = <*p*^2^>(*t*)/*H*^2^ (see Equation 54).

**Appendix 2—figure 3.**
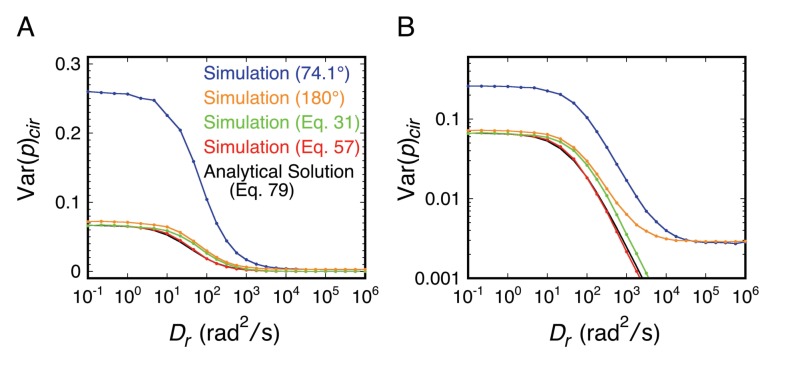
Comparison of the relationship between <*p^2^*>_cir_ (=Var(*p*)_cir_) and *D_r_* as determined analytically and from rotational random walk simulations for an isotropically rotating spherical particle. Results are plotted on both linear (**A**) and log (**B**) ordinate scales. The *red* curve reproduces the *black* curve, providing confirmation of the simulation algorithm. As noted earlier, Equation 57 requires isotropic excitation and *B* = 1. The relatively small difference between the *red* and *green* curves indicates that the error introduced by reducing Equation 31 to Equation 57 is not very high. The *blue* and *orange* curves reach a minimum of ~0.003 at high *D_r_t* values due to the limited photons recovered from the random walk simulations (shot-noise limited). (*black*) Equation 79; (*red*) simulation algorithm using Equation 57 to calculate *p*; (*green*) simulation algorithm using Equation 31 to calculate *p*; (*orange*) rotational random walk simulation (no noise, no threshold) for a perfect objective (θ_obj_ = 180°); (*blue*) simulation data from Figure 2—figure supplement 1C (θ_obj_ = 74.1°). *t* = 10 ms; *N_s_* = 1400; *N* = 10,000.

## Additional files

Supplementary file 1. Table S1: <*p*>_cir_ and <*p^2^*>_cir_ Values, Table S2: <*p*>_lin_ and Var(*p*)_lin_ Values.

Transparent reporting form

## Appendix 1

### Additional considerations for interpreting p-PALM data

10.7554/eLife.28716.035 Rotational anisotropy A probe’s rotational mobility is determined by its three principle rotational diffusion constants *D_x_*, *D_y_*, and *D_z_*, which are related to the average rotational diffusion constant by *D_r_* = (*D_x_* + *D_y_* + *D_z_*)/3. The relationship between the values of these rotational diffusion constants can have a significant effect on the <*p*> and Var(*p*) obtained for a given *D_r_* value. A critical parameter is the angle (γ) between the transition dipole and the major (dominant) rotational axis. Values of γ far from the magic angle of 54.7° (Axelrod, 1989) have a substantial effect on p-PALM measurements under highly anisotropic conditions (Appendix 1—figures 1–3). In most of the reported experiments, the mEos3 probe was tethered to FG-polypeptides via its N- or C-terminus, both of which are at the bottom of its β-barrel structure (Zhang et al., 2012). Consequently, we assumed that the tethered probe’s rotational *z*-axis was well-approximated by its β-barrel axis. The transition dipole of GFP subtends an ~60° angle from the β-barrel axis (Inoué et al., 2002), and we assumed that this is approximately true for mEos3 (Zhang et al., 2012). With these constraints, if the principal rotational diffusion constants were non-identical, we assumed that *z*-axis rotation of the probe at the tip of an FG-polypeptide would be the most facile (Appendix 1—figure 1A). Rotational random walk simulations for various *D_z_*/*D_xy_* ratios assuming *D_z_* > *D_xy_* (where *D*_⊥ _≡ *D_xy_* = *D_x_ =  D_y_*) indicated that for γ = ~60° (i.e, near the magic angle), the *D_z_*/*D_xy_* ratio has little detectable influence on the relationship between <*p*>_lin_ or Var(*p*)_cir_ and *D_r_* (Appendix 1—figure 1B,C). These simulations thus suggest that the mEos3 probe on tip-labeled FG-polypeptides will yield p-PALM data similar to that expected for a free particle. Numerical aperture (NA) For single molecule fluorescence experiments, a high NA objective is typically desired to maximize photon collection efficiency. However, the ranges of <*p*> and Var(*p*) obtained in p-PALM experiments depends on the NA, and both are broader at lower NA values (Appendix 1—figure 4). A major factor that leads to a reduction in the nominal NA is the spherical aberration that results from a refractive index mismatch, which occurs when using an oil immersion lens for an aqueous sample (Appendix 1—figure 5) (Mondal and Disaspro, 2014). An additional, albeit minor, factor reducing the NA can be a high scattering sample, which preferentially results in the loss of photons emitted at high angles relative to the transport axis due to the longer path in the scattering medium (Theer and Denk, 2006). Both of these factors contribute to reducing the NA in the reported p-PALM experiments. The influence of refractive index mismatch/spherical aberration on p-PALM measurements became apparent in control experiments on freely diffusing mEos3 in glycerol solution. Under our experimental conditions, translational motion was too fast to obtain p-PALM data on freely diffusing mEos3 in normal buffer. However, in 92% glycerol, the translational motion of freely diffusing mEos3 was significantly reduced and p-PALM experiments yielded <*p*>_lin_ = 0.47–0.50 (Supplementary file 1; Figure 3—figure supplement 1). Notably, the estimated *D_r_* of ~3 × 10^4^ rad^2^/s (Stokes-Einstein-Debye relation; [Loman et al., 2010]) in this high viscosity solution (η ≈ 380 cP; [Lide, 1998]) is in rotational mobility Regime III, indicating that θ_eff_ < 45° (NA_eff_ < 0.94). Thus, the NA_eff_ was lower when using a 92% glycerol solution (*n* = 1.46; θ_eff_ < 45°) than an aqueous sample (*n* = 1.33, θ_eff_ ≈ 50–60°). This was surprising since spherical aberration should be worse for an aqueous sample than a glycerol sample when using an oil immersion objective due to a larger refractive index mismatch (Appendix 1—figure 5). Additional aberrations may have been introduced by the dual-polarization imaging system (see **Materials and methods**). The range of <*p*>_lin_ for mEos3 within the NPC was ~0.41–0.46 for the various conditions tested (Supplementary file 1). This range is higher than that in Region III of Figure 2A (~0.39). Thresholding, background noise, and ellipticity were not able to explain these differences (Figure 2—figure supplements 4,7), but a reduced NA can. Our linear excitation data (Supplementary file 1) are consistent with an NA_eff_ ≈ 1.02–1.15 in water (θ_eff_ ≈ 50–60°) (Appendix 1—figure 4), which is substantially below the nominal value of the NA = 1.46 (θ_obj_ = 74.1°). The reduced NA is likely a consequence of spherical aberration with a minor contribution from scattering in the sample. Most rotational walk simulations reported in this paper assume θ_obj_ = 74.1°, which corresponds to a non-scattering sample medium and aberration-free imaging with a sample index of refraction matching the immersion oil. These simulations are valid for determining the role of various parameters. However, since simulations with θ_eff_ = 50–60° more closely model the experimental conditions on permeabilized cells, they were used for more precise interpretation of the results (see Mixed populations section in main text). Importantly, our major conclusions are not affected by a moderate uncertainty in the NA_eff_. Sensitivity to binding interactions The sensitivity of the p-PALM approach to binding interactions became apparent when mEos2 (McKinney et al., 2009) was used as a probe. We observed significantly reduced rotational mobility for the probes on mEos2-Nup98 relative to mEos3-Nup98 (Appendix 1—figure 6), consistent with the finding that mEos2 forms dimers (Hoi et al., 2010; McKinney et al., 2009). Dimerization of mEos2 was likely promoted by the high local concentration of such probes within the FG-network. Since the mEos3 probe is a true monomer (Zhang et al., 2012), the reduced rotational mobilities reported here under certain conditions requires an alternate interpretation. As we have argued in this paper, molecular crowding is a reasonable explanation for the low rotational mobilities observed for probes in the FG-network of NPCs under some conditions. Nonetheless, the mEos2 results indicate that p-PALM can be used to directly monitor the binding of fluorescent molecule to a binding partner. Mixed populations Since the p-PALM parameters <*p*> and Var(*p*) are averages over the entire population within the dataset, the rotational mobility behavior of individual sub-populations in more complex samples may not be accurately reflected in these values. In the main text, one example of a mixed population sample (containing molecules with at least two distinct rotational mobilities) was discussed (Figure 6). Here, we further discuss this example and identify additional datasets containing mixed populations. Higher than expected Var(*p*)_cir _values for tip-labeled FG-polypeptides High mobility was expected for probes at the end of long FG-polypeptides, as these were presumed to be positioned at or near the periphery of the FG-network, and thus, in a relatively open environment (sparse polypeptide distribution). Since *D_r_* for mEos3 free in solution is ~10^7^ rad^2^/s (calculated for a sphere [Loman et al., 2010]), a high rotational mobility should yield Var(p)_cir_ = ~0.01–0.02 (*D_r_* > ~10^3^ rad^2^/s; Regimes II-III of Figure 2B). Unexpectedly, the Var(*p*)_cir_ for tip-labeled FG-polypeptides under wildtype conditions was ~0.04–0.06 (Supplementary file 1). While the higher than expected Var(*p*)_cir_ values could be a reflection of an unexpectedly low rotational mobility (Regime IV), we explored whether there might be an alternate explanation. We first considered whether the asymptote at high *D_r_* values in Figure 2B is too low due to an error in the assumed threshold, camera noise, photons collected, the NA_eff_, or the ellipticity. However, reasonable values for these parameters are unable to increase the high *D_r_* asymptote to reach the experimental values (Figure 2—figure supplements 4,5,7, and Appendix 1—figure 4). Notably, the broader ‘wings’ in the polarization histograms for Pom121-mEos3 obtained under wildtype and conditions (Figure 3) were not obtained for the simulations on homogeneous populations (Figure 2). These broad ‘wings’ are consistent with a small sub-population of rotationally constrained molecules (low *D_r_* values) that yield high |*p*| values. Thus, we explored whether a simulated mixture of probes with *D_r_* values < ~10^3^ rad^2^/s and > ~10^3^ rad^2^/s could simultaneously yield a Var(*p*)_cir_, polarization histogram, and photon scatterplot consistent with the data. This was indeed possible, as is demonstrated by the results of such an analysis (Figure 6). We therefore conclude that the higher than expected Var(*p*)_cir_ value for Pom121-mEos3 under wildtype conditions arises from sub-populations of probe molecules with distinct rotational mobilities, which are likely generated by at least two distinct environments. We therefore anticipated that the higher than expected Var(*p*)_cir_ values for other tip-labeled FG-polypeptides could be similarly explained. Rotational mobility near the NPC scaffold We next examined whether probes positioned near the NPC scaffold also exhibited multiple rotational mobilities. The significantly shorter FG-polypeptide segment between the probe and the anchor domain prevents a wide spatial distribution of the probe, which could potentially confine the probe to a more homogeneous environment. Alternately, multiple anchoring sites or different environments in different NPCs could result in distinct rotational mobilities. When attached to two different proteins, mEos3 probes attached near the NPC scaffold exhibited multiple distinct rotational mobilities. We first examined the rotational mobility of the probe on mEos3-^700mid^Nup98, which was attached 12 residues away from the APD anchor domain (Figure 5A). The photon scatterplot for mEos3-^700mid^Nup98 is consistent with a mixed population hypothesis (Figure 6—figure supplement 1C). As a control, the photon scatterplot of mEos3-Nup98 is also consistent with a mixed population hypothesis, although the percentage of the low rotational mobility sub-population appears significantly lower (Figure 6—figure supplement 1A). We also examined the rotational mobility of mEos3 attached to the N-terminus of Pom121, which is near the membrane anchor domain (Figure 4), and found that the photon scatterplot is consistent with a mixed population hypothesis, similar to the C-terminally labeled Pom121 (Figure 6—figure supplement 2). These data indicate the probes attached near the NPC scaffold on Nup98 and Pom121 experienced multiple environments, though it remains unclear whether this was a consequence of local heterogeneity near the anchoring sites within a single NPC, or whether the FG-networks of different NPCs had different compositions and/or structures (e.g., due to different NTR occupancies). Since the probe was in the middle of an FG-polypeptide in the mEos3-^700mid^Nup98 experiments, its rotational mobility behavior could potentially have been very different from that of tip-labeled mutants due to the two attachment points (Appendix 1—figure 1A). Therefore, it was important to examine the assumptions that the rotational anisotropy was low and that γ ≈ ~60°. Rotational random walk simulations where the *D_x_*/*D_yz_* and *D_y_*/*D_xz_* ratios were varied (Appendix 1—figure 2) revealed that values of γ far from the magic angle of 54.7° (Axelrod, 1989) have a substantial effect on p-PALM measurements under highly anisotropic conditions. Allowing γ to vary, we found that γ = ~35–40° or ~70–75° at a high *D_z_*/*D_xy_* ratio (Appendix 1—figure 2) yields Var(*p*)_cir_ values consistent with that observed for mEos3-^700mid^Nup98 (Figure 5B). However, the experimental polarization histograms are inconsistent with these parameters (Figure 6—figure supplement 4). Therefore, we conclude that the relatively high Var(*p*)_cir_ value for the probe on mEos3-^700mid^Nup98 is indeed a consequence of probe sub-populations with distinct *D_r_* values, and not due to highly anisotropic rotational mobility behavior. Increasing Var(*p*)_cir_ by increasing the low mobility fraction in mixed populations. The increased Var(*p*)_cir_ for the mEos3 probe on mEos3-^700mid^Nup98 relative to mEos3-Nup98 (0.12 vs 0.037; Supplementary file 1) coincided with an increase in the fraction of the sub-population with lower rotational mobility (Figure 6—figure supplement 1). We therefore explored whether other increases in Var(p)_cir_ could be explained by an increase in the fraction of a lower mobility sub-population for a variety of other conditions. Photon scatterplots for the Nup98 middle mutants support the hypothesis that the observed increase in Var(p)_cir_ as the probe was moved toward the APD anchor domain was indeed a consequence of an increase in the fraction of a lower mobility sub-population under both wildtype and +Imp β1 conditions (Figure 6—figure supplement 5). The substantial increases in Var(*p*)_cir_ by WGA also are consistent with an increase in the low mobility fraction, though the high mobility fraction also appears to have reduced mobility (Figure 6—figure supplements 6–8). In summary, we consider it likely that most, if not all, of the high Var(*p*)_cir_ values arose from mixed populations, one sub-population of which had a relatively low rotational mobility (<10^3^ rad^2^/s).

## Appendix 2

### Analytical solutions for the Var(*p*)_cir_ dependence on rotational diffusion

10.7554/eLife.28716.042 Definition of *p* The <*p*> and Var(*p*) values from rotational random walk simulations for a variety of conditions were obtained by running the simulations many times for individual molecules and averaging the results. In order to augment and verify the simulations, and guide the choice of the functional form of fits to the simulation data (Equations 25 and 27), we performed analytical calculations for a few special situations under circularly polarized illumination. For simplicity, we have dropped the ‘cir’ subscripts on <*p*> and Var(*p*) in this appendix. Spherical coordinates were defined as:

$$\begin{array}{ll} x & \;\;\;=\cos\theta \sin\varphi \\ y & \;\;\;=\sin\theta \sin\varphi \\ z & \;\;\;=\cos\varphi \end{array}$$

Using Equation 7 (**Materials and methods**) and including the excitation probability, the intensity collected from *m* dipole orientations under high photon flux is:

(28) $$I_{p}=i_{tot}\sum_{k=1}^{m}\left(K_{1}x_{k}^{2}+K_{2}y_{k}^{2}+K_{3}z_{k}^{2}\right)\left(\sin^{2}\varphi_{k}\right)\left(\cos^{2}\theta_{k}+(1/\varepsilon )\sin^{2}\theta_{k}\right)$$

where ε is the ellipticity, as defined earlier (**Materials and methods**). For all of the derivations in this appendix, we have assumed circular excitation (ε = 1) and that the dipole does not rotate between excitation and emission (*D_r_* < ~10^6^ rad^2^/s). Consequently,

(29a) $$I_{p}=i_{tot}\sum_{k=1}^{m}\left(K_{1}x_{k}^{2}+K_{2}y_{k}^{2}+K_{3}z_{k}^{2}\right)\left(\sin^{2}\varphi_{k}\right)$$

(29b) $$I_{p}=i_{tot}\sum_{k=1}^{m}\left(K_{1}\cos^{2}\theta_{k}\sin^{4}\varphi_{k}+K_{2}\sin^{2}\theta_{k}\sin^{4}\varphi_{k}+K_{3}\cos^{2}\varphi_{k}\sin^{2}\varphi_{k}\right)$$

and similarly,

(30a) $$I_{s}=i_{tot}\sum_{k=1}^{m}\left(K_{2}x_{k}^{2}+K_{1}y_{k}^{2}+K_{3}z_{k}^{2}\right)\left(\sin^{2}\varphi_{k}\right)$$

(30b) $$I_{s}=i_{tot}\sum_{k=1}^{m}\left(K_{2}\cos^{2}\theta_{k}\sin^{4}\varphi_{k}+K_{1}\sin^{2}\theta_{k}\sin^{4}\varphi_{k}+K_{3}\cos^{2}\varphi_{k}\sin^{2}\varphi_{k}\right)$$

Substituting the expressions above into the definition of polarization in Equation 3 (**Materials and methods**) and assuming that *g* = 1, the polarization *p* measured for one molecule in elapsed time *t* for *m* dipole orientations (analogous to the rotational walk steps) is given by:

(31) $$p=\frac{I_{p}-I_{s}}{I_{p}+I_{s}}=\frac{\sum_{k=1}^{m}\left(K_{1}-K_{2}\right)\sin^{4}\varphi_{k}\cos2\theta_{k}}{\sum_{k=1}^{m}\left[\left(K_{1}+K_{2}\right)\sin^{4}\varphi_{k}+2K_{3}\sin^{2}\varphi_{k}\cos^{2}\varphi_{k}\right]}=\frac{A\sum_{k=1}^{m}\sin^{4}\varphi_{k}\cos2\theta_{k}}{\sum_{k=1}^{m}\left[(\sin^{4}\varphi_{k}+B\sin^{2}\varphi_{k}\cos^{2}\varphi_{k}\right]}$$

where *A* = (*K_1_ – K_2_*) /(*K_1_* + *K_2_*) and *B* = 2*K_3_* /(*K_1_* + *K_2_*). As discussed earlier, *K_1_*, *K_2_*, and *K_3_* depend on the NA of the microscope objective (Equations 9–11). For θ*_obj_* = 74.1°, *A* = 0.939 and *B* = 0.765. Case 1: *D_r_t* = 0 The upper limit of Var(*p*) is attained when the particles are rotationally immobile, which corresponds to either short collection times or arrested rotational diffusion. This is formally defined as the limit where *D_r_t* → 0. Under these conditions, θ and φ are constant for a given individual particle, and thus, Equation 31 reduces to:

(32) $$p=\frac{A\sin^{2}\varphi }{\sin^{2}\varphi +B\cos^{2}\varphi }\cos2\theta =H\left(A,B,\varphi \right)\cos2\theta$$

where the φ-dependence has been subsumed into the function *H*, which depends on *A*, *B*, and φ. Assuming an isotropic distribution of dipole orientations, the second moment of the polarization distribution, <*p*^2^>, for a very large collection of such particles is obtained by averaging *p* over all angles. Recalling that <*p*> = 0 and Var(*p*) = <*p*^2^> for circular excitation (see **Materials and methods**, Figure 2, and Equations 46 and 65), we get:

(33) $$\text{Var}{\left(p\right)}_{D_{r}t\to 0}=\;<p^{2}{>}_{D_{r}t\to 0}\;=\frac{1}{4\pi }\int_{0}^{\pi }\int_{0}^{2\pi }{\left(\frac{A\sin^{2}\varphi \cos2\theta }{\sin^{2}\varphi +B\cos^{2}\varphi }\right)}^{2}\sin\varphi d\theta d\varphi$$

The integration over θ equates to π, which leads to:

(34) $$\text{Var}{\left(p\right)}_{D_{r}t\to 0}\;=\frac{1}{4}\int_{0}^{\pi }{\left(\frac{A\sin^{2}\varphi }{\sin^{2}\varphi +B\cos^{2}\varphi }\right)}^{2}\sin\varphi d\varphi$$

As *A* and *B* are constant for a given experiment, numerically integrating using values for θ*_obj_* = 74.1° yields:

(35) $$\text{Var}{\left(p\right)}_{D_{r}t\to 0}=\frac{1}{4}\left(1.0117\right)=0.2529$$

Thus, for *D_r_t* → 0 (immobile) and α = 0 (infinite photons), the value of β in Equation 25 is β_0_ = 1/0.2529 = 3.954. Case 2: φ = Constant (rotation on a circle) Definitions In some cases, a dipole may be restricted to rotate around a single rotational axis. For example, a membrane protein could rotate around an axis normal to the membrane – the extent of lateral diffusion is irrelevant, as long as the movement is two-dimensional (no membrane curvature). The orientation of the dipole relative to the membrane normal is not restricted, but remains constant (φ = constant). This situation corresponds to rotation around a circle, or *D_z_* > 0 and *D_x_* = *D_y_* = 0. Under these conditions, Equation 31 reduces to:

(36) $$p=\frac{H\left(A,B,\varphi \right)}{m}\sum_{k=1}^{m}\cos2\theta_{k}$$

where the factor *m* in the denominator normalizes the result based on the number of different orientations of the dipole. This is analogous to the integral along the dipole trajectory,

(37) $$p\left(t,\theta_{0}\right)=\frac{H\left(A,B,\varphi \right)}{t}\int_{0}^{t}dt^{\prime }\cos\left[2\theta \left(t^{\prime },\theta_{0}\right)\right]$$

where θ(*t,* θ_0_) is the value of θ at time *t* given a starting angle of θ_0_. Note that *p*(*t,* θ_0_) is a random variable that depends on the particle's entire angular trajectory. To evaluate its distribution, we need to know the time evolution of θ. Assuming that the dipole motion is an isotropic rotational random walk, the probability for a dipole starting at angle θ_0_ to be at an angle θ at time *t* is given by:

(38) $$q\left(\theta ,t,\theta_{0}\right)=\sum_{n\;=\;-\infty }^{n\;=\;\infty }\frac{1}{\sqrt{4\pi D_{z}t}}\exp\left[-{\left(\theta -\theta_{0}+2\pi n\right)}^{2}/4D_{z}t\right]$$

where *n* is an integer. This wrapped normal distribution is commonly approximated by a von Mises distribution (Watson, 1982):

(39) $$q(\theta ,t,\theta_{0})=\frac{1}{2\pi I_{0}\left(\frac{1}{2D_{z}t}\right)}\exp\left[\frac{\cos(\theta -\theta_{0})}{2D_{z}t}\right]$$

where the normalization factor

(40) $$I_{0}\left(\frac{1}{2D_{z}t}\right)=\frac{1}{2\pi }\int_{-\pi }^{\pi }d\theta \exp\left[\frac{\cos\left(\theta -\theta_{0}\right)}{2D_{z}t}\right]$$

is the modified Bessel function of order 0. Denoting Q(*p*, *t*, θ_0_) as the probability that the value of *p*(*t,* θ_0_) at time *t* is *p*, the mean value of *p*(*t,* θ_0_) at time *t* for a given θ_0_ is:

(41) $$<p>(t,\theta_{0})=\int_{-1}^{1}pdp\cdot Q(p,t,\theta_{0})$$

Assuming a uniform (random) initial angle distribution, the probability to obtain some value *p* is:

(42) $$Q(p,t)=\frac{1}{2\pi }\int_{-\pi }^{\pi }d\theta_{0}Q(p,t,\theta_{0})$$

where 1/2π normalizes the integration over θ_0_. The average *p* at time *t* is therefore:

(43) $$\begin{array}{ll} <p>(t) & =\frac{1}{2\pi }\int_{-1}^{1}pdp\cdot Q(p,t)\\ & =\frac{1}{2\pi }\int_{-1}^{1}pdp\int_{-\pi }^{\pi }d\theta_{0}Q(p,t,\theta_{0})\\ & =\frac{1}{2\pi }\int_{-\pi }^{\pi }d\theta_{0}\int_{-1}^{1}pdp\cdot Q(p,t,\theta_{0})\\ <p>(t) & =\frac{1}{2\pi }\int_{-\pi }^{\pi }d\theta_{0}<p>(t,\theta_{0}) \end{array}$$

Similarly,

(44) $$<p^{2}>(t)=\frac{1}{2\pi }\int_{-\pi }^{\pi }d\theta_{0}<p^{2}>(t,\theta_{0})$$

Mean (<*p*>) With the above definitions, we first obtain <*p*>. Using Equation 39 to evaluate Equation 37:

$$\begin{array}{ll} <p>\left(t,\theta_{0}\right) & =\left\langle \left(\frac{H}{t}\int_{0}^{t}dt^{\prime }\cos\left[2\theta \left(t^{\prime },\theta_{0}\right)\right]\right)\right\rangle \\ & =\frac{H}{t}\int_{0}^{t}dt^{\prime }\int_{-\pi }^{\pi }d\theta \left\langle \cos\left[2\theta \left(t^{\prime },\theta_{0}\right)\right]\right\rangle \\ & =\frac{H}{t}\int_{0}^{t}dt^{\prime }\int_{-\pi }^{\pi }d\theta \cos\left[2\theta \left(t^{\prime },\theta_{0}\right)\right]\cdot q\left(\theta ,t^{\prime },\theta_{0}\right)\\ & =\frac{H}{t}\int_{0}^{t}dt^{\prime }\int_{-\pi }^{\pi }d\theta \frac{\cos(2\theta )}{2\pi I_{0}\left(\frac{1}{2D_{z}t^{\prime }}\right)}\exp\left[\frac{\cos(\theta -\theta_{0})}{2D_{z}t^{\prime }}\right] \end{array}$$

Making the substitution *u* = θ - θ_0_:

$$\begin{array}{ll} <p>\left(t,\theta_{0}\right) & =\frac{H}{t}\int_{0}^{t}dt^{\prime }\int_{-\pi }^{\pi }du\frac{\cos\left(2\left(u+\theta_{0}\right)\right)}{2\pi I_{0}\left(\frac{1}{2D_{z}t^{\prime }}\right)}\exp\left[\frac{\cos\left(u\right)}{2D_{z}t^{\prime }}\right]\\ & =\frac{H}{t}\int_{0}^{t}dt^{\prime }\int_{-\pi }^{\pi }du\frac{\left(\cos2u\cos2\theta_{0}-\sin2u\sin2\theta_{0}\right)}{2\pi I_{0}\left(\frac{1}{2D_{z}t^{\prime }}\right)}\exp\left[\frac{\cos\left(u\right)}{2D_{z}t^{\prime }}\right]\\ & =\frac{H\cos\left(2\theta_{0}\right)}{t}\int_{0}^{t}dt^{\prime }\int_{-\pi }^{\pi }du\frac{\cos2u}{2\pi I_{0}\left(\frac{1}{2D_{z}t^{\prime }}\right)}\exp\left[\frac{\cos(u)}{2D_{z}t^{\prime }}\right] \end{array}$$

or

(45) $$<p>\left(t,\theta_{0}\right)=\frac{H\cos\left(2\theta_{0}\right)}{t}\int_{0}^{t}dt^{\prime }\frac{I_{2}\left(\frac{1}{2D_{z}t^{\prime }}\right)}{I_{0}\left(\frac{1}{2D_{z}t^{\prime }}\right)}$$

where *I_2_*(1/(2*D_z_t*)) is the 'modified Bessel function of order 2'. Using this to evaluate Equation 43, we find that:

(46) $$<p>\left(t\right)=\frac{H}{2\pi t}\left[\int_{-\pi }^{\pi }d\theta_{0}\cos\left(2\theta_{0}\right)\right]\int_{0}^{t}dt^{\prime }\frac{I_{2}\left(\frac{1}{2D_{z}t^{\prime }}\right)}{I_{0}\left(\frac{1}{2D_{z}t^{\prime }}\right)}=0$$

for all *t*. This simply states that the average polarization for circularly polarized excitation is always 0 (due to the first integral over θ_0_), which is clearly indicated by the histograms in Figure 2D. As the integral in Equation 45 will become necessary for calculating <*p*^2^>(*t*), we describe some of its properties. The integral cannot be calculated analytically. However, it can be evaluated numerically. Making the substitution *x* = *D_z_t*', Equation 45 can be rewritten as:

(47) $$<p>\left(t,\theta_{0}\right)=\frac{H\cos\left(2\theta_{0}\right)}{D_{z}t}\int_{0}^{D_{z}t}dx\frac{I_{2}\left(\frac{1}{2x}\right)}{I_{0}\left(\frac{1}{2x}\right)}=H\cos\left(2\theta_{0}\right)\cdot M_{1}\left(D_{z}t\right)$$

defining the new function:

(48) $$M_{1}(D_{z}t)=\frac{1}{D_{z}t}\int_{0}^{D_{z}t}dx\frac{I_{2}\left(\frac{1}{2x}\right)}{I_{0}\left(\frac{1}{2x}\right)}$$

which has the following properties: For small *t* (and thus, small *x*), $\frac{I_{2}\left(\frac{1}{2x}\right)}{I_{0}\left(\frac{1}{2x}\right)}\to 1$ so that $M_{1}(D_{z}t)\to \frac{1}{D_{z}t}\left(D_{z}t\right)=1$ For large *t*, $\frac{I_{2}\left(\frac{1}{2x}\right)}{I_{0}\left(\frac{1}{2x}\right)}\to 0$, the integral converges to 0.2541, and $M_{1}\left(D_{z}t\right)\to 0$ Overall, *M_1_* is well approximated by:

(49) $$M_{1}(D_{z}t)=\frac{0.2541}{0.2541+D_{z}t}=\frac{1}{1+3.935D_{z}t}$$

and more precisely with the higher order Pade-like approximation (Appendix 2—figure 1):

(50) $$\begin{array}{ll} M_{1}\left(D_{z}t\right) & =\frac{0.2541+0.5D_{z}t}{0.2541+D_{z}t+2{\left(D_{z}t\right)}^{2}}\\ & =\frac{1+1.967D_{z}t}{1+\;3.934D_{z}t\;+\;7.868(D_{z}t{)}^{2}} \end{array}$$

Second moment (<*p^2^*>) A similar approach was used to obtain <*p*^2^>. From Equation 37:

(51) $$\begin{array}{ll} <p^{2}>\left(t,\theta_{0}\right) & =\left\langle {\left(\frac{H}{t}\int_{0}^{t}dt^{\prime }\cos\left[2\theta \left(t^{\prime },\theta_{0}\right)\right]\right)}^{2}\right\rangle \\ & =\left\langle \left[\frac{H}{t}\int_{0}^{t}dt^{\prime }\cos\left[2\theta \left(t^{\prime },\theta_{0}\right)\right]\right]\left[\frac{H}{t}\int_{0}^{t}dt"\cos\left[2\theta \left(t",\theta_{0}\right)\right]\right]\right\rangle \\ & =\frac{H^{2}}{t^{2}}\int_{0}^{t}dt^{\prime }\int_{0}^{t}dt^{\prime \prime }<\cos\left[2\theta \left(t^{\prime },\theta_{0}\right)\right]\cdot \cos\left[2\theta \left(t^{\prime \prime },\theta_{0}\right)\right]> \end{array}$$

which can be rewritten as:

(52) $$\begin{array}{ll} <p^{2}>\left(t,\theta_{0}\right) & =\frac{H^{2}}{t^{2}}\int_{0}^{t}dt^{\prime }\int_{0}^{t^{\prime }}dt^{\prime \prime }\int_{-\pi }^{\pi }d\theta^{\prime }\int_{-\pi }^{\pi }d\theta^{\prime \prime }\cos\left(2\theta^{\prime }\right)\cos\left(2\theta^{\prime \prime }\right)q\left(\theta^{\prime \prime },t^{\prime \prime },\theta_{0}\right)q\left(\theta^{\prime },t^{\prime }-t^{\prime \prime },\theta^{\prime \prime }\right)\\ & +\frac{H^{2}}{t^{2}}\int_{0}^{t}dt^{\prime }\int_{t^{\prime }}^{t}dt^{\prime \prime }\int_{-\pi }^{\pi }d\theta^{\prime }\int_{-\pi }^{\pi }d\theta^{\prime \prime }\cos(2\theta^{\prime })\cos(2\theta^{\prime \prime })q(\theta^{\prime },t^{\prime },\theta_{0})q(\theta^{\prime \prime },t^{\prime \prime }-t^{\prime },\theta^{\prime }) \end{array}$$

The second integral in Equation 52 can be rewritten as:

$$\begin{array}{ll} \frac{H^{2}}{t^{2}}\int_{0}^{t}dt^{\prime \prime }\;\;\;\; & \int_{0}^{t^{\prime \prime }}dt^{\prime }\int_{-\pi }^{\pi }d\theta^{\prime }\int_{-\pi }^{\pi }d\theta^{\prime \prime }\cos\left(2\theta^{\prime }\right)\cos\left(2\theta^{\prime \prime }\right)q\left(\theta^{\prime },t^{\prime },\theta_{0}\right)q\left(\theta^{\prime \prime },t^{\prime \prime }-t^{\prime },\theta^{\prime }\right)\\ & =\frac{H^{2}}{t^{2}}\int_{0}^{t}dt^{\prime }\int_{0}^{t^{\prime }}dt^{\prime \prime }\int_{-\pi }^{\pi }d\theta^{\prime }\int_{-\pi }^{\pi }d\theta^{\prime \prime }\cos\left(2\theta^{\prime }\right)\cos\left(2\theta^{\prime \prime }\right)q\left(\theta^{\prime },t^{\prime \prime },\theta_{0}\right)q\left(\theta^{\prime \prime },t^{\prime }-t^{\prime \prime },\theta^{\prime }\right)\\ & =\frac{H^{2}}{t^{2}}\int_{0}^{t}dt^{\prime }\int_{0}^{t^{\prime }}dt^{\prime \prime }\int_{-\pi }^{\pi }d\theta^{\prime }\int_{-\pi }^{\pi }d\theta^{\prime \prime }\cos\left(2\theta^{\prime }\right)\cos\left(2\theta^{\prime \prime }\right)q\left(\theta^{\prime \prime },t^{\prime \prime },\theta_{0}\right)q\left(\theta^{\prime },t^{\prime }-t^{\prime \prime },\theta^{\prime \prime }\right) \end{array}$$

Which is the same as the first integral in Equation 52. Thus,

(53) $$<p^{2}>\left(t,\theta_{0}\right)=\frac{2H^{2}}{t^{2}}\int_{0}^{t}dt^{\prime }\int_{0}^{t^{\prime }}dt^{\prime \prime }\int_{-\pi }^{\pi }d\theta^{\prime }\int_{-\pi }^{\pi }d\theta^{\prime \prime }\cos\left(2\theta^{\prime }\right)\cos\left(2\theta^{\prime \prime }\right)q\left(\theta^{\prime \prime },t^{\prime \prime },\theta_{0}\right)q\left(\theta^{\prime },t^{\prime }-t^{\prime \prime },\theta^{\prime \prime }\right)$$

and

$$\begin{array}{ll} <p^{2}>\left(t\right) & \;\;\;=\frac{H^{2}}{\pi t^{2}}\int_{-\pi }^{\pi }d\theta_{0}\int_{0}^{t}dt^{\prime }\int_{0}^{t^{\prime }}dt^{\prime \prime }\int_{-\pi }^{\pi }d\theta^{\prime }\int_{-\pi }^{\pi }d\theta^{\prime \prime }\cos\left(2\theta^{\prime }\right)\cos\left(2\theta^{\prime \prime }\right)q\left(\theta^{\prime \prime },t^{\prime \prime },\theta_{0}\right)q\left(\theta^{\prime },t^{\prime }-t^{\prime \prime },\theta^{\prime \prime }\right)\\ & \;\;\;=\frac{H^{2}}{\pi t^{2}}\int_{0}^{t}dt^{\prime }\int_{0}^{t^{\prime }}dt^{\prime \prime }\int_{-\pi }^{\pi }d\theta^{\prime }\int_{-\pi }^{\pi }d\theta^{\prime \prime }\cos\left(2\theta^{\prime }\right)\cos\left(2\theta^{\prime \prime }\right)q\left(\theta^{\prime },t^{\prime }-t^{\prime \prime },\theta^{\prime \prime }\right)\int_{-\pi }^{\pi }\frac{d\theta_{0}}{2\pi I_{0}\left(\frac{1}{2D_{z}t"}\right)}\exp\left[\frac{\cos\left(\theta "-\theta_{0}\right)}{2D_{z}t"}\right]\\ & \;\;\;=\frac{H^{2}}{\pi t^{2}}\int_{0}^{t}dt^{\prime }\int_{0}^{t^{\prime }}dt^{\prime \prime }\int_{-\pi }^{\pi }d\theta^{\prime }\int_{-\pi }^{\pi }d\theta^{\prime \prime }\cos(2\theta^{\prime })\cos(2\theta^{\prime \prime })q(\theta^{\prime },t^{\prime }-t^{\prime \prime },\theta^{\prime \prime })\frac{2\pi I_{0}\left(\frac{1}{2D_{z}t^{\prime \prime }}\right)}{2\pi I_{0}\left(\frac{1}{2D_{z}t^{\prime \prime }}\right)}\\ & \;\;\;=\frac{H^{2}}{\pi t^{2}}\int_{0}^{t}dt^{\prime }\int_{0}^{t^{\prime }}dt^{\prime \prime }\int_{-\pi }^{\pi }d\theta^{\prime }\cos(2\theta^{\prime })\int_{-\pi }^{\pi }d\theta^{\prime \prime }\cos(2\theta^{\prime \prime })\frac{\exp\left[\frac{\cos(\theta^{\prime }-\theta^{\prime \prime })}{2D_{z}\left(t^{\prime }-t^{\prime \prime }\right)}\right]}{2\pi I_{0}\left(\frac{1}{2D_{z}\left(t^{\prime }-t^{\prime \prime }\right)}\right)} \end{array}$$

Making the substitution *u* = θ' - θ":

$$\begin{array}{ll} & =\frac{H^{2}}{\pi t^{2}}\int_{0}^{t}dt^{\prime }\int_{0}^{t^{\prime }}dt^{\prime \prime }\int_{-\pi }^{\pi }d\theta^{\prime }\cos\left(2\theta^{\prime }\right)\int_{-\pi }^{\pi }du\cos\left(2\theta^{\prime }-2u\right)\frac{\exp\left[\frac{\cos\left(u\right)}{2D_{z}\left(t^{\prime }-t^{\prime \prime }\right)}\right]}{2\pi I_{0}\left(\frac{1}{2D_{z}\left(t^{\prime }-t^{\prime \prime }\right)}\right)}\\ & =\frac{H^{2}}{\pi t^{2}}\int_{0}^{t}dt^{\prime }\int_{0}^{t^{\prime }}dt^{\prime \prime }\frac{I_{2}\left(\frac{1}{2D_{z}\left(t^{\prime }-t^{\prime \prime }\right)}\right)}{I_{0}\left(\frac{1}{2D_{z}\left(t^{\prime }-t^{\prime \prime }\right)}\right)}\int_{-\pi }^{\pi }d\theta^{\prime }\cos^{2}\left(2\theta^{\prime }\right)\\ & =\frac{H^{2}}{t^{2}}\int_{0}^{t}dt^{\prime }\int_{0}^{t^{\prime }}dt^{\prime \prime }\frac{I_{2}\left(\frac{1}{2D_{z}\left(t^{\prime }-t^{\prime \prime }\right)}\right)}{I_{0}\left(\frac{1}{2D_{z}\left(t^{\prime }-t^{\prime \prime }\right)}\right)} \end{array}$$

or

(54) $$<p^{2}>(t)=\frac{H^{2}}{{\left(D_{z}t\right)}^{2}}\int_{0}^{D_{z}t}ydy\cdot M_{1}(y)=H^{2}\cdot M_{2}(D_{z}t)$$

where:

(55) $$M_{2}(D_{z}t)=\frac{1}{{\left(D_{z}t\right)}^{2}}\int_{0}^{D_{z}t}ydy\cdot M_{1}\left(y\right)$$

Approximating *M_1_*(*y*) as in Equation 50 and numerically integrating to obtain *M_2_*(*D_z_t*), it was determined that <*p*^2^> is well-approximated by (Appendix 2—figure 2):

(56) $$V\text{ar}(p)\;(t)=\;<p^{2}>(t)=\frac{{\left[H\left(A,B,\varphi \right)\right]}^{2}}{1.95+3.68D_{z}t}$$

where Var(*p*) = <*p*^2^> since <*p*> = 0 for all *D_r_* values (Equation 46). Though constant under the constraint of Case 2, *H^2^* depends on φ, and ranges from 0 to ~0.94, assuming that θ*_obj_* = 74.1°. Case 3: General 3D case for a perfect objective (*B* = 1) Definitions The general 3D case is not treatable analytically in an exact fashion since the two summations in Equation 31 are coupled to the path of the same particle and therefore are statistically correlated. However, assuming isotropic excitation (which eliminates the sin^2^φ terms in Equations 29a and 30a) and the specific case where *B* = 1 (which corresponds to θ*_obj_* = 180°, i.e., all photons collected), *p* (defined by Equation 31) reduces to:

(57) $$p=\frac{A}{m}\sum_{k=1}^{m}\sin^{2}\varphi_{k}\cos2\theta_{k}$$

We derive here the dependence of <*p*^2^> on *D_r_t* for the three-dimensional (3D) case where *D_r_* = *D_x_* = *D_y_ *= *D_z_* under the constraints of Equation 57. A 3D rotational random walk can be approximated by the Fisher-von Mises distribution (Watson, 1982), in which the probability density for a particle starting at angles (θ_0_, φ_0_) to be at (θ, φ) at time *t* is given by:

(58) $$q\left(\theta ,\varphi ,t,\theta_{0},\varphi_{0}\right)=\frac{1}{2\pi F_{0}\left(\frac{1}{2D_{r}t},\theta_{0},\varphi_{0}\right)}\exp\left[\frac{\cos\left[\omega \left(\theta ,\varphi ,\theta_{0},\varphi_{0}\right)\right]}{2D_{r}t}\right]$$

where $\omega (\theta ,\varphi ,\theta_{0},\varphi_{0})$ is the angle between the initial and current position vectors on the unit sphere, and

(59) $$F_{0}\left(\frac{1}{2D_{r}t},\theta_{0},\varphi_{0}\right)=\frac{1}{2\pi }\int_{0}^{\pi }d\varphi \sin\varphi \int_{-\pi }^{\pi }d\theta \exp\left[\frac{\cos\left[\omega \left(\theta ,\varphi ,\theta_{0},\varphi_{0}\right)\right]}{2D_{r}t}\right]$$

is the normalization constant. From stereometry,

(60) $$\omega (\theta ,\varphi ,\theta_{0},\varphi_{0})=\cos\varphi \cos\varphi_{0}+\sin\varphi \sin\varphi_{0}\cos(\theta -\theta_{0})$$

and thus:

(61) $$\begin{array}{l} 2\pi F_{0}\left(\frac{1}{2D_{r}t},\theta_{0},\varphi_{0}\right)=\int_{0}^{\pi }d\varphi \sin\varphi \int_{-\pi }^{\pi }d\theta \exp\left[\frac{\cos\varphi \cos\varphi_{0}+\sin\varphi \sin\varphi_{0}\cos\left(\theta -\theta_{0}\right)}{2D_{r}t}\right]\\ F_{0}\left(\frac{1}{2D_{r}t},\varphi_{0}\right)=\int_{0}^{\pi }d\varphi \sin\varphi \exp\left[\frac{\cos\varphi \cos\varphi_{0}}{2D_{r}t}\right]I_{0}\left[\frac{\sin\varphi \sin\varphi_{0}}{2D_{r}t}\right] \end{array}$$

indicating that there is no dependence of *F*_0_ on θ_0_. Mean (*<p>*) With the above definitions, <*p*> is calculated similarly to the 2D case (Case 2), but now including φ rotations. The measured polarization of a single molecule after integrating for time *t* is given by:

(62) $$p\left(t,\theta_{0},\varphi_{0}\right)=\frac{A}{t}\int_{0}^{t}dt^{\prime }\sin^{2}\varphi \left(t^{\prime },\theta_{0},\varphi_{0}\right)\cos\left[2\theta \left(t^{\prime },\theta_{0},\varphi_{0}\right)\right]$$

Therefore, analogous to the derivation of Equation 45:

(63) $$\begin{array}{ll} <p>\;\;\; & \left(t,\theta_{0},\varphi_{0}\right)=\frac{A}{t}\int_{0}^{t}dt^{\prime }<\sin^{2}\varphi \left(t^{\prime },\theta_{0},\varphi_{0}\right)\cos\left[2\theta \left(t^{\prime },\theta_{0},\varphi_{0}\right)\right]>\\ & =\frac{A}{t}\int_{0}^{t}dt^{\prime }\int_{0}^{\pi }\sin^{2}\varphi \sin\varphi d\varphi \int_{-\pi }^{\pi }d\theta \cos2\theta \cdot q\left(\theta ,\varphi ,t^{\prime },\theta_{0},\varphi_{0}\right)\\ & =\frac{A}{t}\int_{0}^{t}dt^{\prime }\int_{0}^{\pi }\sin^{3}\varphi d\varphi \int_{-\pi }^{\pi }\frac{d\theta \cos(2\theta )}{2\pi F_{0}\left(\frac{1}{2D_{r}t^{\prime }},\varphi_{0}\right)}\exp\left[\frac{\cos\varphi \cos\varphi_{0}+\sin\varphi \sin\varphi_{0}\cos(\theta -\theta_{0})}{2D_{r}t^{\prime }}\right] \end{array}$$

Making the substitution *u* = θ - θ_0_:

$$\begin{array}{ll} <p>\left(t,\theta_{0},\varphi_{0}\right) & =\frac{A}{t}\int_{0}^{t}dt^{\prime }\int_{0}^{\pi }\sin^{3}\varphi d\varphi \int_{-\pi }^{\pi }\frac{du\cos\left(2u+2\theta_{0}\right)}{2\pi F_{0}\left(\frac{1}{2D_{r}t^{\prime }},\varphi_{0}\right)}\exp\left[\frac{\cos\varphi \cos\varphi_{0}+\sin\varphi \sin\varphi_{0}\cos\left(u\right)}{2D_{r}t^{\prime }}\right]\\ & =\frac{A\cos2\theta_{0}}{t}\int_{0}^{t}dt^{\prime }\int_{0}^{\pi }\sin^{3}\varphi d\varphi \int_{-\pi }^{\pi }\frac{du\cos2u}{2\pi F_{0}\left(\frac{1}{2D_{r}t^{\prime }},\varphi_{0}\right)}\exp\left[\frac{\cos\varphi \cos\varphi_{0}+\sin\varphi \sin\varphi_{0}\cos\left(u\right)}{2D_{r}t^{\prime }}\right]\\ & =\frac{A\cos2\theta_{0}}{t}\int_{0}^{t}dt^{\prime }\int_{0}^{\pi }\sin^{3}\varphi d\varphi \frac{\exp\left[\frac{\cos\varphi \cos\varphi_{0}}{2D_{r}t^{\prime }}\right]2\pi I_{2}\left(\frac{\sin\varphi \sin\varphi_{0}}{2D_{r}t^{\prime }}\right)}{2\pi F_{0}\left(\frac{1}{2D_{r}t^{\prime }},\varphi_{0}\right)}\\ & =\frac{A\cos2\theta_{0}}{t}\int_{0}^{t}dt^{\prime }\frac{F_{2}\left(\frac{1}{2D_{r}t^{\prime }},\varphi_{0}\right)}{F_{0}\left(\frac{1}{2D_{r}t^{\prime }},\varphi_{0}\right)} \end{array}$$

Where we have defined

(64) $$F_{2}\left(\frac{1}{2D_{r}t},\varphi_{0}\right)=\int_{0}^{\pi }\sin^{3}\varphi d\varphi \exp\left[\frac{\cos\varphi \cos\varphi_{0}}{2D_{r}t}\right]I_{2}\left(\frac{\sin\varphi \sin\varphi_{0}}{2D_{r}t}\right)$$

Thus,

(65) $$<p>\left(t,\theta_{0},\varphi_{0}\right)=\frac{A\cos2\theta_{0}}{D_{r}t}\int_{0}^{D_{r}t}dx\frac{\text{\textbackslash{} }F_{2}\left(\frac{1}{2x},\varphi_{0}\right)}{F_{0}\left(\frac{1}{2x},\varphi_{0}\right)}$$

As for Equation 45, when the expression in Equation 65 is integrated over θ_0_, we obtain <*p*>(*t*) = 0, for all *t*, as expected. Second moment (*<p^2^>*) An approach similar to that used to evaluate Equation 51 was used to obtain <*p*^2^>:

(66) $$\begin{array}{ll} <\;p^{2}\;>\left(t,\theta_{0},\varphi_{0}\right) & =\left\langle {\left(\frac{A}{t}\int_{0}^{t}dt^{\prime }\sin^{2}\varphi \left(t^{\prime },\theta_{0},\varphi_{0}\right)\cos\left[2\theta \left(t^{\prime },\theta_{0},\varphi_{0}\right)\right]\right)}^{2}\right\rangle \\ & =\langle \left[\frac{A}{t}\int_{0}^{t}dt^{\prime }\sin^{2}\varphi \left(t^{\prime },\theta_{0},\varphi_{0}\right)\cos\left[2\theta \left(t^{\prime },\theta_{0},\varphi_{0}\right)\right]\right]\\ & \left[\frac{A}{t}\int_{0}^{t}dt^{\prime \prime }\sin^{2}\varphi \left(t^{\prime \prime },\theta_{0},\varphi_{0}\right)\cos\left[2\theta \left(t^{\prime \prime },\theta_{0},\varphi_{0}\right)\right]\right]\rangle \\ & =\frac{A^{2}}{t^{2}}\int_{0}^{t}dt^{\prime }\int_{0}^{t^{\prime }}dt^{\prime \prime }\langle \sin^{2}\varphi^{\prime }(t^{\prime },\theta_{0},\varphi_{0})\cos\left[2\theta (t^{\prime },\theta_{0},\varphi_{0})\right]\cdot \\ & \sin^{2}\varphi^{\prime \prime }(t^{\prime \prime },\theta_{0},\varphi_{0})\cos\left[2\theta (t^{\prime \prime },\theta_{0},\varphi_{0})\right]\rangle \\ & =\frac{2A^{2}}{t^{2}}\int_{0}^{t}dt^{\prime }\int_{0}^{t^{\prime }}dt^{\prime \prime }\int_{0}^{\pi }\sin^{3}\varphi^{\prime }d\varphi^{\prime }\int_{0}^{\pi }\sin^{3}\varphi^{\prime \prime }d\varphi^{\prime \prime }\\ & \int_{-\pi }^{\pi }d\theta^{\prime \prime }\cos\left(2\theta^{\prime \prime }\right)q\left(\theta^{\prime \prime },\varphi^{\prime \prime },t^{\prime \prime },\theta_{0},\varphi_{0}\right)\\ & \cdot \int_{-\pi }^{\pi }d\theta^{\prime }\cos\left(2\theta^{\prime }\right)q\left(\theta^{\prime },\varphi^{\prime },t^{\prime }-t^{\prime \prime },\theta^{\prime \prime },\varphi^{\prime \prime }\right) \end{array}$$

and

(67) $$\begin{array}{c} <p^{2}>\left(t\right)=\frac{2A^{2}}{4\pi t^{2}}\int_{-\pi }^{\pi }d\theta_{0}\int_{0}^{\pi }\sin\varphi_{0}d\varphi_{0}\int_{0}^{t}dt^{\prime }\int_{0}^{t^{\prime }}dt^{\prime \prime }\int_{0}^{\pi }\sin^{3}\varphi^{\prime }d\varphi^{\prime }\int_{0}^{\pi }\sin^{3}\varphi "d\varphi "\\ \cdot \int_{-\pi }^{\pi }d\theta "\cos\left(2\theta^{\prime \prime }\right)q\left(\theta ",\varphi ",t^{\prime \prime },\theta_{0},\varphi_{0}\right)\int_{-\pi }^{\pi }d\theta^{\prime }\cos\left(2\theta^{\prime }\right)q\left(\theta^{\prime },\varphi^{\prime },t^{\prime }-t^{\prime \prime },\theta^{\prime \prime },\varphi^{\prime \prime }\right) \end{array}$$

where 1/4π normalizes the integrations over θ_0_ and φ_0_. Simplifying:

(68) $$\begin{array}{l} <p^{2}>(t)=\frac{2A^{2}}{4\pi t^{2}}\int_{0}^{t}dt^{\prime }\int_{0}^{t^{\prime }}dt^{\prime \prime }\int_{0}^{\pi }\sin^{3}\varphi^{\prime }d\varphi^{\prime }\int_{0}^{\pi }\sin^{3}\varphi^{\prime \prime }d\varphi^{\prime \prime }\int_{-\pi }^{\pi }d\theta^{\prime \prime }\cos(2\theta^{\prime \prime })\\ \cdot \int_{-\pi }^{\pi }d\theta^{\prime }\cos(2\theta^{\prime })q(\theta^{\prime },\varphi^{\prime },t^{\prime }-t^{\prime \prime },\theta^{\prime \prime },\varphi^{\prime \prime })\int_{-\pi }^{\pi }d\theta_{0}\int_{0}^{\pi }\sin\varphi_{0}d\varphi_{0}q(\theta^{\prime \prime },\varphi^{\prime \prime },t^{\prime \prime },\theta_{0},\varphi_{0}) \end{array}$$

The last two integrals in Equation 68 equate to 1 because (θ", φ", *t*", θ_0_, φ_0_) is symmetric in (θ", φ") and (θ_0_, φ_0_):

$$\begin{array}{ll} & \int_{-\pi }^{\pi }d\theta_{0}\int_{0}^{\pi }\sin\varphi_{0}d\varphi_{0}q(\theta^{\prime \prime },\varphi^{\prime \prime },t^{\prime \prime },\theta_{0},\varphi_{0})\\ & =\int_{-\pi }^{\pi }d\theta^{\prime \prime }\int_{0}^{\pi }\sin\varphi^{\prime \prime }d\varphi^{\prime \prime }q(\theta^{\prime \prime },\varphi^{\prime \prime },t^{\prime \prime },\theta_{0},\varphi_{0})=1 \end{array}$$

Thus, changing the order of integration in Equation 68, we get:

$$\begin{array}{c} <p^{2}>\left(t\right)=\frac{A^{2}}{2\pi t^{2}}\int_{0}^{t}dt"\int_{t"}^{t}dt^{\prime }\int_{0}^{\pi }\sin^{3}\varphi^{\prime }d\varphi^{\prime }\int_{0}^{\pi }\sin^{3}\varphi "d\varphi "\int_{-\pi }^{\pi }d\theta "\cos\left(2\theta^{\prime \prime }\right)\\ \cdot \int_{-\pi }^{\pi }d\theta^{\prime }\cos\left(2\theta^{\prime }\right)q\left(\theta^{\prime },\varphi^{\prime },t^{\prime }-t^{\prime \prime },\theta^{\prime \prime },\varphi^{\prime \prime }\right) \end{array}$$

Making the substitution *t' – t" = z,*

$$\begin{array}{ll} <p^{2}>(t)=\;\;\;\; & \frac{A^{2}}{2\pi t^{2}}\int_{0}^{t}dt"\int_{0}^{t-t"}dz\int_{0}^{\pi }\sin^{3}\varphi^{\prime }d\varphi^{\prime }\int_{0}^{\pi }\sin^{3}\varphi "d\varphi "\int_{-\pi }^{\pi }d\theta "\cos(2\theta^{\prime \prime })\\ & \cdot \int_{-\pi }^{\pi }d\theta^{\prime }\cos(2\theta^{\prime })q(\theta^{\prime },\varphi^{\prime },z,\theta^{\prime \prime },\varphi^{\prime \prime }) \end{array}$$

Recognizing from Equations 63–65 that

$$\int_{0}^{t-t"}dz\int_{0}^{\pi }\sin^{3}\varphi^{\prime }d\varphi^{\prime }\int_{-\pi }^{\pi }d\theta^{\prime }\cos\left(2\theta^{\prime }\right)q\left(\theta^{\prime },\varphi^{\prime },z,\theta^{\prime \prime },\varphi^{\prime \prime }\right)=\cos2\theta^{\prime \prime }\int_{0}^{t-t^{\prime \prime }}dz\frac{F_{2}\left(\frac{1}{2D_{r}z},\varphi^{\prime \prime }\right)}{F_{0}\left(\frac{1}{2D_{r}z},\varphi^{\prime \prime }\right)}$$

we obtain

$$<p^{2}>(t)=\frac{A^{2}}{2\pi t^{2}}\int_{0}^{t}dt"\int_{0}^{\pi }\sin^{3}\varphi "d\varphi "\int_{-\pi }^{\pi }d\theta "\cos^{2}(2\theta^{\prime \prime })\int_{0}^{t-t"}dz\frac{\;F_{2}\left(\frac{1}{2D_{r}z},\varphi^{\prime \prime }\right)}{F_{0}\left(\frac{1}{2D_{r}z},\varphi^{\prime \prime }\right)}$$

Making the variable substitutions *x* = *D_r_(t - t''*) and *y* = *D_r_z*, we obtain

$$<p^{2}>\left(t\right)=\frac{A^{2}}{2{\left(D_{r}t\right)}^{2}}\int_{0}^{\pi }\sin^{3}\varphi "d\varphi "\int_{0}^{D_{r}t}dx\int_{0}^{x}dy\frac{F_{2}\left(\frac{1}{2y},\varphi^{\prime \prime }\right)}{F_{0}\left(\frac{1}{2y},\varphi^{\prime \prime }\right)}$$

and finally,

(69) $$<p^{2}>\left(t\right)=\frac{A^{2}}{2{\left(D_{r}t\right)}^{2}}\int_{0}^{\pi }\sin^{3}\varphi "d\varphi "\int_{0}^{D_{r}t}xG\left(x,\varphi^{\prime \prime }\right)dx$$

where

(70) $$G\left(x,\varphi^{\prime \prime }\right)=\frac{1}{x}\int_{0}^{x}dy\frac{\;F_{2}\left(\frac{1}{2y},\varphi^{\prime \prime }\right)}{F_{0}\left(\frac{1}{2y},\varphi^{\prime \prime }\right)}$$

To numerically evaluate Equation 69, we first examine two limits. D_r_t → 0 For small values of *D_r_t*, *G* can be approximated by:

(71) $$\underset{x\to 0}{lim}\;G\left(x,\varphi^{\prime \prime }\right)\approx \sin^{2}\varphi "$$

and therefore,

(72) $$\begin{array}{ll} <p^{2}>{\left(t\right)}_{D_{r}t\to 0} & \approx \frac{A^{2}}{2{\left(D_{r}t\right)}^{2}}\int_{0}^{\pi }\sin^{3}\varphi "\sin^{2}\varphi "d\varphi "\int_{0}^{D_{r}t}xdx\\ <p^{2}>{\left(t\right)}_{D_{r}t\to 0} & =\frac{A^{2}}{4}\int_{0}^{\pi }\sin^{5}\varphi "d\varphi " \end{array}$$

Integration yields:

(73) $$<p^{2}>{\left(t\right)}_{D_{r}t\to 0}=\frac{A^{2}}{4}\left(\frac{16}{15}\right)=\frac{4A^{2}}{15}=\frac{1}{15}$$

since *A* = 0.5 under the constraint that θ*_obj_* = 180°. *D_r_t* → *∞* For large values of *D_r_t, G* can be approximated by:

(74) $$G\left(x,\varphi^{\prime \prime }\right)\approx \frac{M\left(\varphi^{\prime \prime }\right)}{x}\;where\;M\left(\varphi^{\prime \prime }\right)=\int_{0}^{\infty }dy\frac{F_{2}\left(\frac{1}{2y},\varphi^{\prime \prime }\right)}{F_{0}\left(\frac{1}{2y},\varphi^{\prime \prime }\right)}$$

Numerical evaluation reveals that *M* is well approximated by:

(75) $$M\left(\varphi^{\prime \prime }\right)\approx \frac{\sin^{2}\varphi^{\prime \prime }}{5.2}$$

Using Equations 71, 74 and 75, the function *G*(*x*, φ") can be well approximated with a Pade-like expression over the entire range of possible *D_r_t* values as:

(76) $$G\left(x,\varphi^{\prime \prime }\right)\approx \frac{M\left(\varphi^{\prime \prime }\right)}{(1/5.2)+x}=\frac{M\left(\varphi^{\prime \prime }\right)}{0.192+x}$$

Therefore,

(77) $$\begin{array}{ll} <p^{2}>{\left(t\right)}_{D_{r}t\to \infty } & \approx \frac{A^{2}}{2{\left(D_{r}t\right)}^{2}}\int_{0}^{\pi }\sin^{3}\varphi "d\varphi "\int_{0}^{D_{r}t}\frac{M\left(\varphi^{\prime \prime }\right)xdx}{0.192+x}\\ & =\frac{A^{2}}{2{\left(D_{r}t\right)}^{2}}\int_{0}^{\pi }\sin^{3}\varphi "d\varphi "M\left(\varphi^{\prime \prime }\right)\int_{0}^{D_{r}t}\left(1-\frac{0.192}{0.192+x}\right)dx\\ & =\frac{A^{2}}{2{\left(D_{r}t\right)}^{2}}\int_{0}^{\pi }\sin^{3}\varphi "d\varphi "M\left(\varphi^{\prime \prime }\right)\left(D_{r}t-0.192\ln\left(0.192+D_{r}t\right)\right) \end{array}$$

Since *D_r_t >>* 0.192ln(0.192 *+ D_r_t*) for large values of *D_r_t,* using Equation 75,

(78) $$<p^{2}>{\left(t\right)}_{D_{r}t\to \infty }\approx \frac{A^{2}}{5.2\left(2D_{r}t\right)}\int_{0}^{\pi }\sin^{5}\varphi "d\varphi "$$

which, for *A* = 0.5 (θ*_obj_* = 180°), reduces to

$$<p^{2}>{\left(t\right)}_{D_{r}t\to \infty }=\frac{A^{2}}{5.2\left(2D_{r}t\right)}\left(\frac{16}{15}\right)=\frac{1}{39D_{r}t}$$

General expression Combining the results from the two limits, and assuming a Pade-like expression for <*p^2^*>, we obtain:

(79) $$V\text{ar}(p)\;(t)=\;<p^{2}>(t)\approx \frac{1}{15+39D_{r}t}$$

Appendix 2—figure 3 demonstrates good agreement between Equation 79 and the simulation algorithm.

## References

[bib1] Abu-Arish A, Kalab P, Ng-Kamstra J, Weis K, Fradin C (2009). Spatial distribution and mobility of the Ran GTPase in live interphase cells. Biophysical Journal.

[bib2] Adam SA, Marr RS, Gerace L (1990). Nuclear protein import in permeabilized mammalian cells requires soluble cytoplasmic factors. The Journal of Cell Biology.

[bib3] Adam V, Moeyaert B, David CC, Mizuno H, Lelimousin M, Dedecker P, Ando R, Miyawaki A, Michiels J, Engelborghs Y, Hofkens J (2011). Rational design of photoconvertible and biphotochromic fluorescent proteins for advanced microscopy applications. Chemistry & Biology.

[bib4] Akey CW, Goldfarb DS (1989). Protein import through the nuclear pore complex is a multistep process. The Journal of Cell Biology.

[bib5] Antonin W, Franz C, Haselmann U, Antony C, Mattaj IW (2005). The integral membrane nucleoporin pom121 functionally links nuclear pore complex assembly and nuclear envelope formation. Molecular Cell.

[bib6] Atkinson CE, Mattheyses AL, Kampmann M, Simon SM (2013). Conserved spatial organization of FG domains in the nuclear pore complex. Biophysical Journal.

[bib7] Aumiller WM, Davis BW, Keating CD (2014). Phase separation as a possible means of nuclear compartmentalization. International Review of Cell and Molecular Biology.

[bib8] Axelrod D (1979). Carbocyanine dye orientation in red cell membrane studied by microscopic fluorescence polarization. Biophysical Journal.

[bib9] Axelrod D (1989). Fluorescence polarization microscopy. Methods in Cell Biology.

[bib10] Bajar B, Wang E, Zhang S, Lin M, Chu J (2016). A guide to fluorescent protein FRET Pairs. Sensors.

[bib11] Betzig E, Patterson GH, Sougrat R, Lindwasser OW, Olenych S, Bonifacino JS, Davidson MW, Lippincott-Schwartz J, Hess HF (2006). Imaging intracellular fluorescent proteins at nanometer resolution. Science.

[bib12] Bischoff FR, Görlich D (1997). RanBP1 is crucial for the release of RanGTP from importin beta-related nuclear transport factors. FEBS Letters.

[bib13] Bischoff FR, Klebe C, Kretschmer J, Wittinghofer A, Ponstingl H (1994). RanGAP1 induces GTPase activity of nuclear Ras-related Ran. PNAS.

[bib14] Chao J, Ram S, Ward ES, Ober RJ (2013). Ultrahigh accuracy imaging modality for super-localization microscopy. Nature Methods.

[bib15] Chatel G, Desai SH, Mattheyses AL, Powers MA, Fahrenkrog B (2012). Domain topology of nucleoporin Nup98 within the nuclear pore complex. Journal of Structural Biology.

[bib16] Chook YM, Blobel G (2001). Karyopherins and nuclear import. Current Opinion in Structural Biology.

[bib17] Chook YM, Süel KE (2011). Nuclear import by karyopherin-βs: Recognition and inhibition. Biochimica Et Biophysica Acta (BBA) - Molecular Cell Research.

[bib18] Coalson RD, Eskandari Nasrabad A, Jasnow D, Zilman A (2015). A polymer-brush-based nanovalve controlled by nanoparticle additives: Design principles. The Journal of Physical Chemistry B.

[bib19] Cronshaw JM, Krutchinsky AN, Zhang W, Chait BT, Matunis MJ (2002). Proteomic analysis of the mammalian nuclear pore complex. The Journal of Cell Biology.

[bib20] Dabauvalle MC, Schulz B, Scheer U, Peters R (1988). Inhibition of nuclear accumulation of karyophilic proteins in living cells by microinjection of the lectin wheat germ agglutinin. Experimental Cell Research.

[bib21] Dange T, Grünwald D, Grünwald A, Peters R, Kubitscheck U (2008). Autonomy and robustness of translocation through the nuclear pore complex: a single-molecule study. The Journal of Cell Biology.

[bib22] Denning DP, Patel SS, Uversky V, Fink AL, Rexach M (2003). Disorder in the nuclear pore complex: the FG repeat regions of nucleoporins are natively unfolded. PNAS.

[bib23] Denning DP, Rexach MF (2007). Rapid evolution exposes the boundaries of domain structure and function in natively unfolded FG nucleoporins. Molecular & Cellular Proteomics.

[bib24] Dix JA, Verkman AS (2008). Crowding effects on diffusion in solutions and cells. Annual Review of Biophysics.

[bib25] Durisic N, Laparra-Cuervo L, Sandoval-Álvarez Ángel, Borbely JS, Lakadamyali M (2014). Single-molecule evaluation of fluorescent protein photoactivation efficiency using an in vivo nanotemplate. Nature Methods.

[bib26] Eisele NB, Labokha AA, Frey S, Görlich D, Richter RP (2013). Cohesiveness tunes assembly and morphology of FG nucleoporin domain meshworks - Implications for nuclear pore permeability. Biophysical Journal.

[bib27] Eriksson C, Rustum C, Hallberg E (2004). Dynamic properties of nuclear pore complex proteins in gp210 deficient cells. FEBS Letters.

[bib28] Eskandari Nasrabad A, Jasnow D, Zilman A, Coalson RD (2016). Precise control of polymer coated nanopores by nanoparticle additives: Insights from computational modeling. The Journal of Chemical Physics.

[bib29] Fahrenkrog B, Aebi U (2003). The nuclear pore complex: nucleocytoplasmic transport and beyond. Nature Reviews Molecular Cell Biology.

[bib30] Fahrenkrog B, Maco B, Fager AM, Köser J, Sauder U, Ullman KS, Aebi U (2002). Domain-specific antibodies reveal multiple-site topology of Nup153 within the nuclear pore complex. Journal of Structural Biology.

[bib31] Feng W, Benko AL, Lee JH, Stanford DR, Hopper AK (1999). Antagonistic effects of NES and NLS motifs determine *S. cerevisiae* Rna1p subcellular distribution. Journal of Cell Science.

[bib32] Finlay DR, Forbes DJ (1990). Reconstitution of biochemically altered nuclear pores: transport can be eliminated and restored. Cell.

[bib33] Forkey JN, Quinlan ME, Goldman YE (2000). Protein structural dynamics by single-molecule fluorescence polarization. Progress in Biophysics and Molecular Biology.

[bib34] Forkey JN, Quinlan ME, Goldman YE (2005). Measurement of single macromolecule orientation by total internal reflection fluorescence polarization microscopy. Biophysical Journal.

[bib35] Frey S, Görlich D (2007). A saturated FG-repeat hydrogel can reproduce the permeability properties of nuclear pore complexes. Cell.

[bib36] Frey S, Görlich D (2009). FG/FxFG as well as GLFG repeats form a selective permeability barrier with self-healing properties. The EMBO Journal.

[bib37] Frey S, Richter RP, Görlich D (2006). FG-rich repeats of nuclear pore proteins form a three-dimensional meshwork with hydrogel-like properties. Science.

[bib38] Frosst P, Guan T, Subauste C, Hahn K, Gerace L (2002). Tpr is localized within the nuclear basket of the pore complex and has a role in nuclear protein export. The Journal of Cell Biology.

[bib39] Funakoshi T, Clever M, Watanabe A, Imamoto N (2011). Localization of Pom121 to the inner nuclear membrane is required for an early step of interphase nuclear pore complex assembly. Molecular Biology of the Cell.

[bib40] Ghavami A, Veenhoff LM, van der Giessen E, Onck PR (2014). Probing the disordered domain of the nuclear pore complex through coarse-grained molecular dynamics simulations. Biophysical Journal.

[bib41] Gould TJ, Gunewardene MS, Gudheti MV, Verkhusha VV, Yin SR, Gosse JA, Hess ST (2008). Nanoscale imaging of molecular positions and anisotropies. Nature Methods.

[bib42] Griffis ER, Xu S, Powers MA (2003). Nup98 localizes to both nuclear and cytoplasmic sides of the nuclear pore and binds to two distinct nucleoporin subcomplexes. Molecular Biology of the Cell.

[bib43] Grünwald D, Singer RH, Rout M (2011). Nuclear export dynamics of RNA-protein complexes. Nature.

[bib44] Grünwald D, Singer RH (2010). In vivo imaging of labelled endogenous β-actin mRNA during nucleocytoplasmic transport. Nature.

[bib45] Güttler T, Görlich D (2011). Ran-dependent nuclear export mediators: a structural perspective. The EMBO Journal.

[bib46] Ha T, Laurence TA, Chemla DS, Weiss S (1999). Polarization spectroscopy of single fluorescent molecules. The Journal of Physical Chemistry B.

[bib47] Hallberg E, Wozniak RW, Blobel G (1993). An integral membrane protein of the pore membrane domain of the nuclear envelope contains a nucleoporin-like region. The Journal of Cell Biology.

[bib48] Harms GS, Sonnleitner M, Schütz GJ, Gruber HJ, Schmidt T (1999). Single-molecule anisotropy imaging. Biophysical Journal.

[bib49] Hase ME, Cordes VC (2003). Direct interaction with nup153 mediates binding of Tpr to the periphery of the nuclear pore complex. Molecular Biology of the Cell.

[bib50] Hodel AE, Hodel MR, Griffis ER, Hennig KA, Ratner GA, Xu S, Powers MA (2002). The three-dimensional structure of the autoproteolytic, nuclear pore-targeting domain of the human nucleoporin Nup98. Molecular Cell.

[bib51] Hoi H, Shaner NC, Davidson MW, Cairo CW, Wang J, Campbell RE (2010). A monomeric photoconvertible fluorescent protein for imaging of dynamic protein localization. Journal of Molecular Biology.

[bib52] Hough LE, Dutta K, Sparks S, Temel DB, Kamal A, Tetenbaum-Novatt J, Rout MP, Cowburn D (2015). The molecular mechanism of nuclear transport revealed by atomic-scale measurements. eLife.

[bib53] Huang B, Babcock H, Zhuang X (2010). Breaking the diffraction barrier: super-resolution imaging of cells. Cell.

[bib54] Huang B, Bates M, Zhuang X (2009). Super-resolution fluorescence microscopy. Annual Review of Biochemistry.

[bib55] Hutten S, Flotho A, Melchior F, Kehlenbach RH (2008). The Nup358-RanGAP complex is required for efficient importin alpha/beta-dependent nuclear import. Molecular Biology of the Cell.

[bib56] Hülsmann BB, Labokha AA, Görlich D (2012). The permeability of reconstituted nuclear pores provides direct evidence for the selective phase model. Cell.

[bib57] Inoué S, Shimomura O, Goda M, Shribak M, Tran PT (2002). Fluorescence polarization of green fluorescence protein. PNAS.

[bib58] Izaurralde E, Kutay U, von Kobbe C, Mattaj IW, Görlich D (1997). The asymmetric distribution of the constituents of the Ran system is essential for transport into and out of the nucleus. The EMBO Journal.

[bib59] Jamali T, Jamali Y, Mehrbod M, Mofrad MR (2011). Nuclear pore complex: biochemistry and biophysics of nucleocytoplasmic transport in health and disease. International review of cell and molecular biology.

[bib60] Kampmann M, Atkinson CE, Mattheyses AL, Simon SM (2011). Mapping the orientation of nuclear pore proteins in living cells with polarized fluorescence microscopy. Nature Structural & Molecular Biology.

[bib61] Kapinos LE, Schoch RL, Wagner RS, Schleicher KD, Lim RY (2014). Karyopherin-centric control of nuclear pores based on molecular occupancy and kinetic analysis of multivalent binding with FG nucleoporins. Biophysical Journal.

[bib62] Kosinski J, Mosalaganti S, von Appen A, Teimer R, DiGuilio AL, Wan W, Bui KH, Hagen WJ, Briggs JA, Glavy JS, Hurt E, Beck M (2016). Molecular architecture of the inner ring scaffold of the human nuclear pore complex. Science.

[bib63] Krull S, Thyberg J, Björkroth B, Rackwitz HR, Cordes VC (2004). Nucleoporins as components of the nuclear pore complex core structure and Tpr as the architectural element of the nuclear basket. Molecular Biology of the Cell.

[bib64] Kubitscheck U, Grünwald D, Hoekstra A, Rohleder D, Kues T, Siebrasse JP, Peters R (2005). Nuclear transport of single molecules: dwell times at the nuclear pore complex. The Journal of Cell Biology.

[bib65] Kutay U, Bischoff FR, Kostka S, Kraft R, Görlich D (1997a). Export of importin alpha from the nucleus is mediated by a specific nuclear transport factor. Cell.

[bib66] Kutay U, Izaurralde E, Bischoff FR, Mattaj IW, Görlich D (1997b). Dominant-negative mutants of importin-beta block multiple pathways of import and export through the nuclear pore complex. The EMBO Journal.

[bib67] Kuznetsova IM, Turoverov KK, Uversky VN (2014). What macromolecular crowding can do to a protein. International Journal of Molecular Sciences.

[bib68] Lakowicz JR (2006). Principles of Fluorescence Spectroscopy.

[bib69] Leterrier JF (2001). Water and the cytoskeleton. Cellular and Molecular Biology.

[bib70] Lide D (1998). CRC Handbook of Chemistry and Physics.

[bib71] Lieleg O, Ribbeck K (2011). Biological hydrogels as selective diffusion barriers. Trends in Cell Biology.

[bib72] Lim RY, Fahrenkrog B, Köser J, Schwarz-Herion K, Deng J, Aebi U (2007). Nanomechanical basis of selective gating by the nuclear pore complex. Science.

[bib73] Lim RY, Huang NP, Köser J, Deng J, Lau KH, Schwarz-Herion K, Fahrenkrog B, Aebi U (2006). Flexible phenylalanine-glycine nucleoporins as entropic barriers to nucleocytoplasmic transport. PNAS.

[bib74] Lim RY, Ullman KS, Fahrenkrog B (2008). Biology and biophysics of the nuclear pore complex and its components. International Review of Cell and Molecular Biology.

[bib75] Lin DH, Stuwe T, Schilbach S, Rundlet EJ, Perriches T, Mobbs G, Fan Y, Thierbach K, Huber FM, Collins LN, Davenport AM, Jeon YE, Hoelz A (2016). Architecture of the symmetric core of the nuclear pore. Science.

[bib76] Loman A, Gregor I, Stutz C, Mund M, Enderlein J (2010). Measuring rotational diffusion of macromolecules by fluorescence correlation spectroscopy. Photochem. Photobiol. Sci..

[bib77] Lowe AR, Tang JH, Yassif J, Graf M, Huang WYC, Groves JT, Weis K, Liphardt JT (2015). Importin-β modulates the permeability of the nuclear pore complex in a Ran-dependent manner. eLife.

[bib78] Lyman SK, Guan T, Bednenko J, Wodrich H, Gerace L (2002). Influence of cargo size on Ran and energy requirements for nuclear protein import. The Journal of Cell Biology.

[bib79] Löschberger A, Franke C, Krohne G, van de Linde S, Sauer M (2014). Correlative super-resolution fluorescence and electron microscopy of the nuclear pore complex with molecular resolution. Journal of Cell Science.

[bib80] Löschberger A, van de Linde S, Dabauvalle MC, Rieger B, Heilemann M, Krohne G, Sauer M (2012). Super-resolution imaging visualizes the eightfold symmetry of gp210 proteins around the nuclear pore complex and resolves the central channel with nanometer resolution. Journal of Cell Science.

[bib81] Mahajan R, Delphin C, Guan T, Gerace L, Melchior F (1997). A small ubiquitin-related polypeptide involved in targeting RanGAP1 to nuclear pore complex protein RanBP2. Cell.

[bib82] Maimon T, Elad N, Dahan I, Medalia O (2012). The human nuclear pore complex as revealed by cryo-electron tomography. Structure.

[bib83] Makise M, Mackay DR, Elgort S, Shankaran SS, Adam SA, Ullman KS (2012). The Nup153-Nup50 protein interface and its role in nuclear import. Journal of Biological Chemistry.

[bib84] Mattheyses AL, Kampmann M, Atkinson CE, Simon SM (2010). Fluorescence anisotropy reveals order and disorder of protein domains in the nuclear pore complex. Biophysical Journal.

[bib85] Matunis MJ, Wu J, Blobel G (1998). SUMO-1 modification and its role in targeting the Ran GTPase-activating protein, RanGAP1, to the nuclear pore complex. The Journal of Cell Biology.

[bib86] McGuffee SR, Elcock AH (2010). Diffusion, crowding & protein stability in a dynamic molecular model of the bacterial cytoplasm. PLoS Computational Biology.

[bib87] McKinney SA, Murphy CS, Hazelwood KL, Davidson MW, Looger LL (2009). A bright and photostable photoconvertible fluorescent protein. Nature Methods.

[bib88] Mi L, Goryaynov A, Lindquist A, Rexach M, Yang W (2015). Quantifying nucleoporin stoichiometry inside single nuclear pore complexes in vivo. Scientific Reports.

[bib89] Milles S, Mercadante D, Aramburu IV, Jensen MR, Banterle N, Koehler C, Tyagi S, Clarke J, Shammas SL, Blackledge M, Gräter F, Lemke EA (2015). Plasticity of an ultrafast interaction between nucleoporins and nuclear transport receptors. Cell.

[bib90] Mitrea DM, Kriwacki RW (2016). Phase separation in biology; functional organization of a higher order. Cell Communication and Signaling.

[bib91] Moeyaert B, Nguyen Bich N, De Zitter E, Rocha S, Clays K, Mizuno H, van Meervelt L, Hofkens J, Dedecker P (2014). Green-to-red photoconvertible Dronpa mutant for multimodal super-resolution fluorescence microscopy. ACS Nano.

[bib92] Mohr D, Frey S, Fischer T, Güttler T, Görlich D (2009). Characterisation of the passive permeability barrier of nuclear pore complexes. The EMBO Journal.

[bib93] Mondal PP, Disaspro A (2014). Fundamentals of Fluorescence Microscopy: Exploring Life with Light.

[bib94] Mortensen KI, Churchman LS, Spudich JA, Flyvbjerg H (2010). Optimized localization analysis for single-molecule tracking and super-resolution microscopy. Nature Methods.

[bib95] Musser SM, Grünwald D (2016). Deciphering the structure and function of nuclear pores using single-molecule fluorescence approaches. Journal of Molecular Biology.

[bib96] Mutch SA, Fujimoto BS, Kuyper CL, Kuo JS, Bajjalieh SM, Chiu DT (2007). Deconvolving single-molecule intensity distributions for quantitative microscopy measurements. Biophysical Journal.

[bib97] Nakielny S, Shaikh S, Burke B, Dreyfuss G (1999). Nup153 is an M9-containing mobile nucleoporin with a novel Ran-binding domain. The EMBO Journal.

[bib98] Nishijima H, Nakayama J, Yoshioka T, Kusano A, Nishitani H, Shibahara K, Nishimoto T (2006). Nuclear RanGAP is required for the heterochromatin assembly and is reciprocally regulated by histone H3 and Clr4 histone methyltransferase in *Schizosaccharomyces pombe*. Molecular Biology of the Cell.

[bib99] Okamura M, Inose H, Masuda S (2015). RNA Export through the NPC in Eukaryotes. Genes.

[bib100] Olivini F, Beretta S, Chirico G (2001). Two-photon fluorescence polarization anisotropy decay on highly diluted solutions by phase fluorometry. Applied Spectroscopy.

[bib101] Ori A, Banterle N, Iskar M, Andrés-Pons A, Escher C, Khanh Bui H, Sparks L, Solis-Mezarino V, Rinner O, Bork P, Lemke EA, Beck M (2013). Cell type-specific nuclear pores: a case in point for context-dependent stoichiometry of molecular machines. Molecular Systems Biology.

[bib102] Osmanović D, Ford IJ, Hoogenboom BW (2013). Model inspired by nuclear pore complex suggests possible roles for nuclear transport receptors in determining its structure. Biophysical Journal.

[bib103] Otsuka S, Bui KH, Schorb M, Hossain MJ, Politi AZ, Koch B, Eltsov M, Beck M, Ellenberg J (2016). Nuclear pore assembly proceeds by an inside-out extrusion of the nuclear envelope. eLife.

[bib104] Panté N, Kann M (2002). Nuclear pore complex is able to transport macromolecules with diameters of about 39 nm. Molecular Biology of the Cell.

[bib105] Paradise A, Levin MK, Korza G, Carson JH (2007). Significant proportions of nuclear transport proteins with reduced intracellular mobilities resolved by fluorescence correlation spectroscopy. Journal of Molecular Biology.

[bib106] Patel SS, Belmont BJ, Sante JM, Rexach MF (2007). Natively unfolded nucleoporins gate protein diffusion across the nuclear pore complex. Cell.

[bib107] Peleg O, Lim RY (2010). Converging on the function of intrinsically disordered nucleoporins in the nuclear pore complex. Biological Chemistry.

[bib108] Pleiner T, Bates M, Trakhanov S, Lee CT, Schliep JE, Chug H, Böhning M, Stark H, Urlaub H, Görlich D (2016). Correction: Nanobodies: site-specific labeling for super-resolution imaging, rapid epitope-mapping and native protein complex isolation. eLife.

[bib109] Popken P, Ghavami A, Onck PR, Poolman B, Veenhoff LM (2015). Size-dependent leak of soluble and membrane proteins through the yeast nuclear pore complex. Molecular Biology of the Cell.

[bib110] Radu A, Moore MS, Blobel G (1995). The peptide repeat domain of nucleoporin Nup98 functions as a docking site in transport across the nuclear pore complex. Cell.

[bib111] Ren Y, Seo HS, Blobel G, Hoelz A (2010). Structural and functional analysis of the interaction between the nucleoporin Nup98 and the mRNA export factor Rae1. PNAS.

[bib112] Reverter D, Lima CD (2005). Insights into E3 ligase activity revealed by a SUMO-RanGAP1-Ubc9-Nup358 complex. Nature.

[bib113] Rexach M, Blobel G (1995). Protein import into nuclei: association and dissociation reactions involving transport substrate, transport factors, and nucleoporins. Cell.

[bib114] Ribbeck K, Görlich D (2001). Kinetic analysis of translocation through nuclear pore complexes. The EMBO Journal.

[bib115] Rosenblum JS, Blobel G (1999). Autoproteolysis in nucleoporin biogenesis. PNAS.

[bib116] Rout MP, Aitchison JD, Suprapto A, Hjertaas K, Zhao Y, Chait BT (2000). The yeast nuclear pore complex: composition, architecture, and transport mechanism. The Journal of Cell Biology.

[bib117] Rout MP, Aitchison JD (2001). The nuclear pore complex as a transport machine. Journal of Biological Chemistry.

[bib118] Schleicher KD, Dettmer SL, Kapinos LE, Pagliara S, Keyser UF, Jeney S, Lim RY (2014). Selective transport control on molecular velcro made from intrinsically disordered proteins. Nature Nanotechnology.

[bib119] Schwefel D, Maierhofer C, Beck JG, Seeberger S, Diederichs K, Möller HM, Welte W, Wittmann V (2010). Structural basis of multivalent binding to wheat germ agglutinin. Journal of the American Chemical Society.

[bib120] Shah S, Forbes DJ (1998). Separate nuclear import pathways converge on the nucleoporin Nup153 and can be dissected with dominant-negative inhibitors. Current Biology.

[bib121] Siomi MC, Eder PS, Kataoka N, Wan L, Liu Q, Dreyfuss G (1997). Transportin-mediated nuclear import of heterogeneous nuclear RNP proteins. The Journal of Cell Biology.

[bib122] Stavru F, Hülsmann BB, Spang A, Hartmann E, Cordes VC, Görlich D (2006). NDC1: a crucial membrane-integral nucleoporin of metazoan nuclear pore complexes. The Journal of Cell Biology.

[bib123] Stewart M (2007). Molecular mechanism of the nuclear protein import cycle. Nature Reviews Molecular Cell Biology.

[bib124] Stoffler D, Fahrenkrog B, Aebi U (1999). The nuclear pore complex: from molecular architecture to functional dynamics. Current Opinion in Cell Biology.

[bib125] Stoffler D, Feja B, Fahrenkrog B, Walz J, Typke D, Aebi U (2003). Cryo-electron tomography provides novel insights into nuclear pore architecture: implications for nucleocytoplasmic transport. Journal of Molecular Biology.

[bib126] Strawn LA, Shen T, Shulga N, Goldfarb DS, Wente SR (2004). Minimal nuclear pore complexes define FG repeat domains essential for transport. Nature Cell Biology.

[bib127] Stuwe T, von Borzyskowski LS, Davenport AM, Hoelz A (2012). Molecular basis for the anchoring of proto-oncoprotein Nup98 to the cytoplasmic face of the nuclear pore complex. Journal of Molecular Biology.

[bib128] Sun C, Fu G, Ciziene D, Stewart M, Musser SM (2013). Choreography of importin-α/CAS complex assembly and disassembly at nuclear pores. PNAS.

[bib129] Sun C, Yang W, Tu LC, Musser SM (2008). Single-molecule measurements of importin alpha/cargo complex dissociation at the nuclear pore. PNAS.

[bib130] Suntharalingam M, Wente SR (2003). Peering through the pore: nuclear pore complex structure, assembly, and function. Developmental Cell.

[bib131] Szymborska A, de Marco A, Daigle N, Cordes VC, Briggs JA, Ellenberg J (2013). Nuclear pore scaffold structure analyzed by super-resolution microscopy and particle averaging. Science.

[bib132] Söderqvist H, Hallberg E (1994). The large C-terminal region of the integral pore membrane protein, POM121, is facing the nuclear pore complex. European journal of cell biology.

[bib133] Söderqvist H, Imreh G, Kihlmark M, Linnman C, Ringertz N, Hallberg E (1997). Intracellular distribution of an integral nuclear pore membrane protein fused to green fluorescent protein--localization of a targeting domain. European Journal of Biochemistry.

[bib134] Tagliazucchi M, Peleg O, Kröger M, Rabin Y, Szleifer I (2013). Effect of charge, hydrophobicity, and sequence of nucleoporins on the translocation of model particles through the nuclear pore complex. PNAS.

[bib135] Talamas JA, Hetzer MW (2011). POM121 and Sun1 play a role in early steps of interphase NPC assembly. The Journal of Cell Biology.

[bib136] Testa I, Schönle A, von Middendorff C, Geisler C, Medda R, Wurm CA, Stiel AC, Jakobs S, Bossi M, Eggeling C, Hell SW, Egner A (2008). Nanoscale separation of molecular species based on their rotational mobility. Optics Express.

[bib137] Tetenbaum-Novatt J, Hough LE, Mironska R, McKenney AS, Rout MP (2012). Nucleocytoplasmic transport: a role for nonspecific competition in karyopherin-nucleoporin interactions. Molecular & Cellular Proteomics.

[bib138] Theer P, Denk W (2006). On the fundamental imaging-depth limit in two-photon microscopy. Journal of the Optical Society of America A.

[bib139] Timney BL, Raveh B, Mironska R, Trivedi JM, Kim SJ, Russel D, Wente SR, Sali A, Rout MP (2016). Simple rules for passive diffusion through the nuclear pore complex. The Journal of Cell Biology.

[bib140] Tokunaga M, Imamoto N, Sakata-Sogawa K (2008). Highly inclined thin illumination enables clear single-molecule imaging in cells. Nature Methods.

[bib141] Toretsky JA, Wright PE (2014). Assemblages: functional units formed by cellular phase separation. The Journal of Cell Biology.

[bib142] Tran EJ, Wente SR (2006). Dynamic nuclear pore complexes: life on the edge. Cell.

[bib143] Tu LC, Fu G, Zilman A, Musser SM (2013). Large cargo transport by nuclear pores: implications for the spatial organization of FG-nucleoporins. The EMBO Journal.

[bib144] Tu LC, Musser SM (2011). Single molecule studies of nucleocytoplasmic transport. Biochimica et Biophysica Acta (BBA) - Molecular Cell Research.

[bib145] Ullman KS, Shah S, Powers MA, Forbes DJ (1999). The nucleoporin nup153 plays a critical role in multiple types of nuclear export. Molecular Biology of the Cell.

[bib146] Vovk A, Gu C, Opferman MG, Kapinos LE, Lim RY, Coalson RD, Jasnow D, Zilman A (2016). Simple biophysics underpins collective conformations of the intrinsically disordered proteins of the Nuclear Pore Complex. eLife.

[bib147] Wagner RS, Kapinos LE, Marshall NJ, Stewart M, Lim RY (2015). Promiscuous binding of Karyopherinβ1 modulates FG nucleoporin barrier function and expedites NTF2 transport kinetics. Biophysical Journal.

[bib148] Walther TC, Pickersgill HS, Cordes VC, Goldberg MW, Allen TD, Mattaj IW, Fornerod M (2002). The cytoplasmic filaments of the nuclear pore complex are dispensable for selective nuclear protein import. The Journal of Cell Biology.

[bib149] Watson GS (1982). Distributions on the circle and sphere. Journal of Applied Probability.

[bib150] Wente SR, Rout MP (2010). The nuclear pore complex and nuclear transport. Cold Spring Harbor Perspectives in Biology.

[bib151] Wiedenmann J, Ivanchenko S, Oswald F, Schmitt F, Röcker C, Salih A, Spindler KD, Nienhaus GU (2004). EosFP, a fluorescent marker protein with UV-inducible green-to-red fluorescence conversion. PNAS.

[bib152] Winterflood CM, Ewers H (2014). Single-molecule localization microscopy using mCherry. ChemPhysChem.

[bib153] Wolff B, Willingham MC, Hanover JA (1988). Nuclear protein import: specificity for transport across the nuclear pore. Experimental Cell Research.

[bib154] Wu J, Matunis MJ, Kraemer D, Blobel G, Coutavas E (1995). Nup358, a cytoplasmically exposed nucleoporin with peptide repeats, Ran-GTP binding sites, zinc fingers, a cyclophilin A homologous domain, and a leucine-rich region. Journal of Biological Chemistry.

[bib155] Wälde S, Thakar K, Hutten S, Spillner C, Nath A, Rothbauer U, Wiemann S, Kehlenbach RH (2012). The nucleoporin Nup358/RanBP2 promotes nuclear import in a cargo- and transport receptor-specific manner. Traffic.

[bib156] Yamada J, Phillips JL, Patel S, Goldfien G, Calestagne-Morelli A, Huang H, Reza R, Acheson J, Krishnan VV, Newsam S, Gopinathan A, Lau EY, Colvin ME, Uversky VN, Rexach MF (2010). A bimodal distribution of two distinct categories of intrinsically disordered structures with separate functions in FG nucleoporins. Molecular & Cellular Proteomics.

[bib157] Yang W, Gelles J, Musser SM (2004). Imaging of single-molecule translocation through nuclear pore complexes. PNAS.

[bib158] Yang W, Musser SM (2006a). Nuclear import time and transport efficiency depend on importin beta concentration. The Journal of Cell Biology.

[bib159] Yang W, Musser SM (2006b). Visualizing single molecules interacting with nuclear pore complexes by narrow-field epifluorescence microscopy. Methods.

[bib160] Yokoyama N, Hayashi N, Seki T, Panté N, Ohba T, Nishii K, Kuma K, Hayashida T, Miyata T, Aebi U, Fukuil M, Nishimoto T (1995). A giant nucleopore protein that binds Ran/TC4. Nature.

[bib161] Yoneda Y, Imamoto-Sonobe N, Yamaizumi M, Uchida T (1987). Reversible inhibition of protein import into the nucleus by wheat germ agglutinin injected into cultured cells. Experimental Cell Research.

[bib162] Zhang M, Chang H, Zhang Y, Yu J, Wu L, Ji W, Chen J, Liu B, Lu J, Liu Y, Zhang J, Xu P, Xu T (2012). Rational design of true monomeric and bright photoactivatable fluorescent proteins. Nature Methods.

[bib163] Zhou HX, Rivas G, Minton AP (2008). Macromolecular crowding and confinement: biochemical, biophysical, and potential physiological consequences. Annual Review of Biophysics.

