# Supplementary material for: Investigating molecular crowding within nuclear pores using polarization-PALM
Source: eLife. 2017 Sep 26;6:e28716. doi: 10.7554/eLife.28716 (PMC5693140; doi:10.7554/eLife.28716)
Supplement: Supplementary file 1. [file elife-28716-supp1.pdf]

Table S1: $<p>_{cir}$ and $<p^2>_{cir}$ Values[a]

Sample	$<p>_{cir} \pm SD$	$<p^2>_{cir} \pm SD$	$N_{photons}$	N
Pom121-mEos3:				
Wildtype	0.014 ± 0.010	0.042 ± 0.016	376	3464
+ 10 μM Imp β1	0.022 ± 0.008	0.062 ± 0.017	349	2197
+ 10 μM Imp β2	0.021 ± 0.010	0.052 ± 0.006	348	2622
+ 1 mg/mL WGA	0.024 ± 0.019	0.17 ± 0.03	372	5374
+ 10% Hexanediol	0.043 ± 0.015	0.062 ± 0.01	460	1679
mEos3-Pom121:				
Wildtype	0.021 ± 0.015	0.060 ± 0.019	372	3561
+ 10 μM Imp β1	0.021 ± 0.017	0.066 ± 0.013	371	2036
+ 10 μM Imp β2	0.023 ± 0.013	0.077 ± 0.014	339	2240
+ 1 mg/mL WGA	0.037 ± 0.011	0.11 ± 0.01	375	2121
Nup153-mEos3:				
Wildtype	0.002 ± 0.004	0.037 ± 0.006	332	2526
+ 10 μM Imp β1	0.017 ± 0.003	0.044 ± 0.012	359	2876
+ 10 μM Imp β2	0.016 ± 0.008	0.058 ± 0.010	338	2548
+ 1 mg/mL WGA	0.042 ± 0.018	0.13 ± 0.04	356	2210
mEos3-Nup153:				
Wildtype	0.019 ± 0.009	0.052 ± 0.010	371	3689
+ 10 μM Imp β1	0.016 ± 0.015	0.061 ± 0.013	357	2634
+ 10 μM Imp β2	0.026 ± 0.013	0.087 ± 0.018	353	2022
+ 1 mg/mL WGA	0.020 ± 0.023	0.13 ± 0.01	380	3074
RanGAP-mEos3:				
Wildtype	-0.009 ± 0.006	0.056 ± 0.020	351	3010
+ 10 μM Imp β1	0.002 ± 0.011	0.059 ± 0.028	356	2178
+ 10 μM Imp β2	0.002 ± 0.009	0.12 ± 0.01	334	2410
+ 1 mg/mL WGA	0.045 ± 0.005	0.20 ± 0.02	356	3040
+ 4% Formaldehyde	0.087 ± 0.019	0.27 ± 0.03	322	2548
mEos3-Nup98:				
Wildtype	0.015 ± 0.007	0.037 ± 0.010	411	2243
+ 10 μM Imp β1	0.014 ± 0.012	0.048 ± 0.014	337	2181
+ 10 μM Imp β2	0.019 ± 0.008	0.044 ± 0.004	365	2051
+ 1 mg/mL WGA	0.060 ± 0.011	0.19 ± 0.02	363	2739
mEos3:				
In 92% glycerol (all points)[b]	0.007 ± 0.001	0.034 ± 0.002	314	38380
In 92% glycerol (2nd points)[c]	0.007 ± 0.005	0.027 ± 0.008	378	458
Adsorbed on a coverslip[d]	0.12 ± 0.014	0.28 ± 0.01	342	2604

mEos3-Nup98 Middle Mutants[e]:				
N-terminus	0.015 ± 0.007	0.037 ± 0.010	411	2243
110mid	0.013 ± 0.003	0.056 ± 0.012	397	3730
400mid	0.012 ± 0.007	0.039 ± 0.003	397	2525
500mid	0.039 ± 0.011	0.082 ± 0.007	366	3131
700mid	0.012 ± 0.016	0.12 ± 0.02	351	2841
mEos3-Nup98 Middle Mutants[e] (+ 10 µM Imp β1):				
N-terminus	0.014 ± 0.012	0.048 ± 0.014	337	2181
110mid	0.018 ± 0.005	0.044 ± 0.012	358	2412
400mid	0.019 ± 0.001	0.052 ± 0.006	357	2769
500mid	0.019 ± 0.009	0.073 ± 0.021	357	2577
700mid	0.027 ± 0.006	0.11 ± 0.02	353	2243
mEos3-Nup98 Middle Mutants[e] (+ 1 mg/mL WGA):				
N-terminus	0.060 ± 0.011	0.19 ± 0.02	363	2739
110mid	0.040 ± 0.013	0.24 ± 0.02	367	4563
400mid	0.047 ± 0.018	0.29 ± 0.04	329	3107
500mid	0.053 ± 0.014	0.21 ± 0.02	382	2617
700mid	0.033 ± 0.024	0.18 ± 0.04	371	2391
mEos3-Nup98 Tip Mutants[e]:				
N-terminus	0.015 ± 0.007	0.037 ± 0.010	411	2243
110tip	-0.006 ± 0.014	0.034 ± 0.014	401	1789
110tip + 1 mg/mL WGA	0.027 ± 0.028	0.19 ± 0.009	459	1220
400tip	-0.002 ± 0.006	0.031 ± 0.007	382	2212
mEos2-Nup98:				
Wildtype	0.004 ± 0.012	0.097 ± 0.011	353	3660

[a]*N* individual measurements were pooled into 4 datasets of equal size. The standard deviation (*SD*) was calculated from the 4 independently determined average values. *SD*s were carried to the same decimal value as the mean, which was assumed to be accurate to 2 significant figures, or the nearest thousandth. The mean ± SD approximately represents the 85% confidence interval (see Figure 2 – Supplement 6). The effect of the average number of photons collected ($N_{photons}$) on $\langle p \rangle_{cir}$ and $\mathrm{Var}(p)_{cir}$ values is illustrated in Figure 2 – Supplement 5. Theoretically, for circular excitation, $\langle p^2 \rangle_{cir} = \mathrm{Var}(p)_{cir}$ since $\langle p \rangle_{cir} = 0$ (see Methods), which was approximately true in most cases.
[b]Data collected by using intensities from all points in the trajectories for every spot observed. Most trajectories were observed as a single spot.
[c]Data collected by using intensities from the second point in the trajectory for trajectories consisting of at least three frames. This approach eliminates the possibility of photoactivation or photobleaching during data acquisition, and was used for all p-PALM data (unless otherwise indicated). Due to translational diffusion, trajectories with ≥ 3 points were rare occurrences in 92% glycerol.
[d]Coverslip adsorbed mEos3 yielded results inconsistent with isotropic orientations, likely due to non-random adsorbed orientations and bias (due to flow or coverslip defects). Nonetheless, p-PALM measurements indicate low rotational mobility.
[e]Nup98 middle mutants were generated by inserting mEos3 at the indicated amino acid position from the N-terminus. Tip mutants were generated by deleting the indicated number of amino acids from the N-terminus and then attaching mEos3 (see Figure 1 – Supplement 2).

Table S2: $\langle p \rangle_{lin}$ and $\mathrm{Var}(p)_{lin}$ Values[a]

Sample	$\langle p \rangle_{lin} \pm SD$	$\mathrm{Var}(p)_{lin} \pm SD$	$N_{photons}$	N
Pom121-mEos3:				
Wildtype	0.42 ± 0.03	0.024 ± 0.044	394	558
+ 10 µM Imp β1	0.45 ± 0.01	0.028 ± 0.024	298	855
+ 1 mg/mL WGA	0.42 ± 0.08	0.074 ± 0.115	395	922
10% Hexanediol	0.45 ± 0.02	0.0375 ± 0.030	392	325
Nup153-mEos3:				
Wildtype	0.41 ± 0.01	0.012 ± 0.017	469	757
mEos3-Nup153:				
Wildtype	0.46 ± 0.01	0.018 ± 0.017	305	959
mEos3:				
In 92% glycerol (all points)[b]	0.50 ± 0.01	0.030 ± 0.017	240	16720
In 92% glycerol (2nd points)[c]	0.47 ± 0.01	0.019 ± 0.017	306	246
Adsorbed on a coverslip[d]	0.44 ± 0.02	0.12 ± 0.04	390	844
mEos3-Nup98 Middle Mutants[e]:				
N-terminus	0.41 ± 0.02	0.022 ± 0.030	371	1100
110mid	0.43 ± 0.02	0.025 ± 0.030	272	675
400mid	0.44 ± 0.02	0.016 ± 0.030	413	296
500mid	0.44 ± 0.05	0.036 ± 0.077	415	454
700mid	0.42 ± 0.02	0.024 ± 0.035	384	1010
mEos3-Nup98 Tip Mutants[e]:				
N-terminus	0.41 ± 0.02	0.022 ± 0.030	371	1100
110tip	0.42 ± 0.03	0.014 ± 0.044	313	565

[a] *N* individual measurements were pooled into 4 datasets of equal size. The standard deviation (*SD*) was calculated from the 4 independently determined average values. *SD*s were carried to the same decimal value as the mean, which was assumed to be accurate to 2 significant figures, or the nearest thousandth. The mean ± SD approximately represents the 85% confidence interval (see Figure 2 – Supplement 6). The effect of the average number of photons collected ($N_{photons}$) on $\langle p \rangle_{cir}$ and $\mathrm{Var}(p)_{cir}$ values is illustrated in Figure 2 – Supplement 5. As discussed in Methods, $\mathrm{Var}(p) = \langle p^2 \rangle - \langle p \rangle$.

[b] Data collected by using intensities from all points in the trajectories for every spot observed. Most trajectories were observed as a single spot.

[c] Data collected by using intensities from the second point in the trajectory for trajectories consisting of at least three frames. This approach eliminates the possibility of photoactivation or photobleaching during data acquisition, and was used for all p-PALM data (unless otherwise indicated). Due to translational diffusion, trajectories with ≥ 3 points were rare occurrences in 92% glycerol.

[d] Coverslip adsorbed mEos3 yielded results inconsistent with isotropic orientations, likely due to non-random adsorbed orientations and bias (due to flow or coverslip defects). Nonetheless, p-PALM measurements indicate low rotational mobility.

[e] Nup98 middle mutants were generated by inserting mEos3 at the indicated amino acid position from the N-terminus. Tip mutants were generated by deleting the indicated number of amino acids from the N-terminus and then attaching mEos3 (see Figure 1 – Supplement 2).
